# Rotaxane synthesis *via* a dynamic [2]catenane-ring-opening, axle-cleaving double cross metathesis

**DOI:** 10.1039/d5ra07142a

**Published:** 2025-12-01

**Authors:** M. Mustafa Cetin, Arindam Mazumdar, David B. Cordes, Vahap Gazi Fidan, Zhen Yang, Michael F. Mayer

**Affiliations:** a Department of Chemistry, İstinye University Sarıyer İstanbul 34396 Türkiye mustafa.cetin@istinye.edu.tr mustafamcetin@yahoo.com; b Department of Chemistry and Biochemistry, Texas Tech University MS-41062 Lubbock TX 79409 USA mf.mayer@ttu.edu; c EaStCHEM School of Chemistry, University of St. Andrews St. Andrews KY16 9ST UK; d Department of Chemical and Biological Engineering, Koç University Sarıyer İstanbul 34450 Türkiye; e Strait Institute of Flexible Electronics (SIFE, Future Technologies), Fujian Key Laboratory of Flexible Electronics, Fujian Normal University and Strait Laboratory of Flexible Electronics (SLoFE) Fuzhou 350117 China; f Synthetic Organic and Polymer Research (SOPR) Laboratory, İstinye University Sarıyer İstanbul 34396 Türkiye; g Clean Energy Research Center (TEAM), İstinye University Sarıyer İstanbul 34396 Türkiye

## Abstract

Efficient routes to [2]rotaxanes are often compromised by formation of irrecoverable, non-interlocked byproducts. Herein, we report a thermodynamically steered, atom-economical strategy that couples a Cu(i)-templated, low-strain Sauvage-type [2]catenane with di-stoppered olefin *via* ring-opening double cross-metathesis (RO-DCM), implementing dynamic covalent chemistry to bias the system toward the most stable interlocked architecture. The transformation proceeds through ring opening of the metalated [2]catenane and its *in situ* “insertion” into the axle, engaging internal olefins on both partners. Optimization of metathesis parameters (Grubbs II, DCM, 40 °C) identified the stoichiometry of the di-stoppered olefin as the key lever; using ten equivalents furnished the metalated [2]rotaxane 6 in up to 88% isolated yield while suppressing mono-stoppered byproducts. Subsequent demetalation cleanly delivered [2]rotaxane 9. Analytical size-exclusion chromatography across the full component set provided diagnostic retention times, confirming product identity and the absence of catenane contamination. No dethreading of macrocycle 1 from 9 was detected under conventional heating in DCM or DMSO over 12–48 hours, underscoring kinetic persistence of the mechanical bond. Overall, this RO-DCM platform minimizes non-interlocked waste streams while providing a concise, high-yield entry to [2]rotaxanes from metathesis-addressable, copper-templated interlocks. Beyond the single-molecule level, the approach establishes a general ring-chain equilibration blueprint that should translate to sequence-defined, mechanically interlocked oligomers and polymers.

## Abstract 2

Efficient routes to [2]rotaxanes are often compromised by formation of irrecoverable, non-interlocked byproducts.

## Graphical abstract

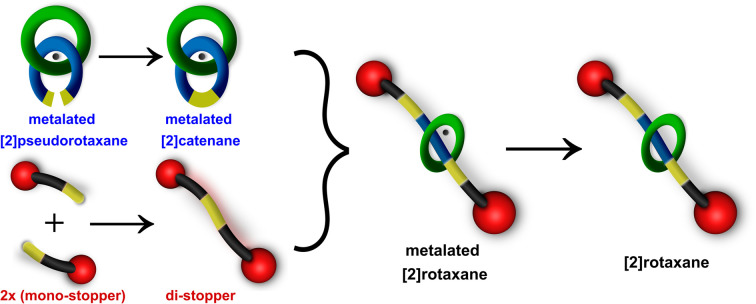

## Introduction

Apart from the aesthetic appeal, the intrinsically dynamic nature of mechanically-interlocked molecules (MIMs) makes them even more interesting. The nature of the mechanical bond between the two interlocked components such as rotaxanes and catenanes encodes programmable, stimulus-responsive motion in a mechanically-bonded architecture, making them archetypal building blocks for molecular machines. In these systems, a mechanical bond between components permits controlled, often reversible, relative motion in response to external stimuli such as redox conditions, pressure, pH, thermal changes, or applied mechanical force. Light can also trigger topology interconversion in MIMs as seen in the example of photodriven rotaxane to catenane transformation *via* reversible anthracene dimerization.^1^ This responsive mobility forms the foundation for molecular machines, which over the past four decades have evolved into sophisticated constructs including molecular shuttles,^2–6^ muscles,^7–12^ switches,^13–15^ and drug carriers.^16–18^

The underlying theme of chemical systems classifiable under the rubric of dynamic covalent chemistry (DCC) is that such systems allow a redistribution of covalent bonds between substructural components where net redistribution is tantamount to a thermodynamic re-equilibration under altered conditions. DCC involves reversible covalent bond formation and exchange, enabling adaptive, self-healing, and recyclable materials. The mechanistic rationale centers on the interplay between thermodynamics and kinetics, with bond exchange processes classified as associative or dissociative, and their behavior modulated by catalysts, temperature, and network structure. DCC mechanisms are typically divided into four mechanistic categories and molecular basis. (i) Associative (exchange without bond dissociation): the network maintains constant crosslinking density, as seen in vitrimers, where bond exchange occurs *via* a concerted pathway. This is rare; most systems involve some degree of bond dissociation.^19,20^ (ii) Dissociative (bond breaking precedes new bond formation): the network temporarily loses crosslinks, leading to changes in material properties.^19–21^ (iii) Thermodynamic control: product distribution is determined by the relative stability of products, not just reaction rates, and external stimuli (temperature, pH, catalysts) shift equilibrium, enabling reversible assembly/disassembly.^21–23^ (iv) Kinetic considerations: the rate of bond exchange, influenced by catalysts or additives, directly affects viscoelastic and mechanical properties.^19,20,24^ Thus, DCC plays a pivotal role in constructing such superior versatile molecular architectures through reversible covalent bond formation. Under thermodynamic control, DCC allows the reorganization of molecular subcomponents, enabling error correction and amplification of desired products, especially in the presence of guiding templates. This strategy in an unprecedented molecular precision manner offers significant advantages for generating functional systems. When thermodynamics and kinetics are compared, kinetics determines how quickly materials respond to stimuli, crucial for applications like 3D printing or self-healing while thermodynamics governs equilibrium.^20–22^ Dynamic covalent processes are governed by well-defined mechanistic categories—associative and dissociative exchange—modulated by thermodynamic and kinetic factors. Catalysts, network design, and external stimuli enable precise control over material properties,^19,20,24–26^ supporting the development of adaptive, recyclable, and self-healing polymers. Building on these DCC foundations, we harness thermodynamic control to orchestrate a reversible ring-chain equilibration in which a Cu(i)-templated, metathesis-addressable [2]catenane undergoes ring-opening double cross-metathesis (RO-DCM) with a di-stoppered axle, effecting *in situ* “insertion” and channeling the system toward the most stable [2]rotaxane while suppressing non-interlocked byproducts. In this way, the abstract DCC principles of error-correction and product amplification translate into a concise, atom-economical route to [2]rotaxanes and a general blueprint for sequence-defined interlocked polymers.

Among MIMs, rotaxanes^27^—molecules where a macrocycle is threaded onto a dumbbell-shaped axle and trapped by bulky stoppers—are central to the field of mechanically interlocked molecules and they occupy a unique position due to their modular architecture and wide functional versatility. Traditionally, their synthesis has relied on four main strategies: slipping, clipping, threading–capping, and the active template approach.^28^ While the thermodynamically-driven slipping method laid early groundwork, the kinetically-controlled clipping and threading–capping techniques have offered more reliable access to rotaxanes. However, these methods often suffer from the formation of irrecoverable non-interlocked byproducts, such as free macrocycles or stopper-functionalized axles, significantly limiting their efficiency and scalability. Rotaxane synthesis—active template synthesis,^27,29–32^ template-directed clipping,^33^ end-capping and swelling,^34,35^ stereoselective and enantioselective synthesis,^29,36–41^ functional and sequence-controlled rotaxanes,^42–46^ and many more—has evolved rapidly, with new strategies enabling greater control over structure, chirality, and function, opening doors to applications in molecular machines, catalysis, and materials science.^27^ Recent research has delivered a diverse toolkit for rotaxane synthesis, including highly selective, efficient, and versatile methods. Advances in chiral and sequence-controlled synthesis are enabling new applications in catalysis, materials, and molecular machinery, with ongoing innovation in both methodology and functional design.^27^

Rotaxanes as mechanically interlocked molecules with a macrocycle threaded onto an axle are foundational in supramolecular chemistry^47^ and have rapidly expanded into polymer science, materials chemistry, catalysis, sensing, and more. Their unique dynamic and switchable properties enable advanced functions in smart materials, electronics, catalysis, and molecular machines. They also have various key applications in the areas of supramolecular and polymer chemistry^47–54^ as smart and tough materials,^47–49,54^ stimuli-responsive systems,^50–52^ and preprogrammed assembly,^53^ sensing and optoelectronics^47,55–57^ as chemo-/biosensors^47,55,56^ and mechanoluminescent materials,^57^ catalysis and synthetic chemistry^47,58,59^ as catalytic platforms and superbases,^59^ molecular machines and advanced functions^47,51,60,61^ as molecular switches and machines,^51,60,61^ and many more. Thus, rotaxanes are enabling breakthroughs in supramolecular and polymer chemistry, with expanding roles in smart materials, sensing, catalysis, and molecular machines. Their dynamic, tunable properties continue to drive innovation across multiple scientific and technological domains.^47^

**Scheme 1:**
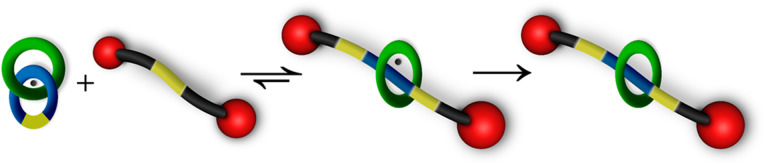
A novel, viable thermodynamically-controlled synthesis of a [2]rotaxane from metalated [2]rotaxane *via* dynamic ring-chain equilibration and then demetalation.

Recently, our approach to rotaxanes synthesis has utilized the concept of DCC which involves cleaving of the axle to allow pseudorotaxane formation followed by a subsequent thermodynamically-controlled transformation into a rotaxane. Aiming to achieve versatile MIMs, we unveiled a novel, viable thermodynamically-controlled synthesis of a metalated [2]rotaxane *via* the *in situ* “insertion” of a metalated [2]catenane into a di-stoppered axle (Scheme 1). In contrast to previous methods, this route occurs *via* a dynamic ring-chain equilibration of copper-chelated [2]catenane and an acyclic chain transfer agent by avoiding undesired dead-end byproducts, opening a new avenue to [2]rotaxanes. This newly developed strategy thus not only extends the synthetic methods for [2]rotaxanes but also brings a new insights into constructing previously inaccessible mechanically-interlocked polymers.

## Results and discussion

Launching from the design logic of dynamic covalent chemistry, we conceived a thermodynamically governed ring-opening double cross-metathesis that inserts a metalated [2]catenane into a di-stoppered axle, thereby forging the target [2]rotaxane with minimal dead-end byproducts. Under equilibrium control, DCC enables error-correction and amplifies the most stable interlocked product, providing an ideal platform for such reversible bond choreography.^62^ Mechanistically, our strategy harnesses established ring-opening (cross) metathesis/cross metathesis (RO(C)M/CM) manifolds with Ru-carbene catalysts (Grubbs II/Hoveyda–Grubbs),^63,64^ which excel in ring opening and cross metathesis of internal olefins under mild conditions.^65,66^ The reverse transformation—conversion of a crown-ether-based active-template synthesis (CEATS) [2]rotaxane into a [2]catenane by ring-closing metathesis (RCM) under high-dilution conditions using Grubbs I—has also been studied by Barlow and Evans.^67^ A Sauvage-type Cu(i) catenane serves as a low-strain yet metathesis-addressable precursor, aligning with precedents on copper-templated interlocks and their reactivity.^63,68–70^ Together, these elements converge to deliver an atom-economic route to [2]rotaxanes while foreshadowing access to mechanically interlocked polymers that were previously out of reach.^71^

**Scheme 2:**
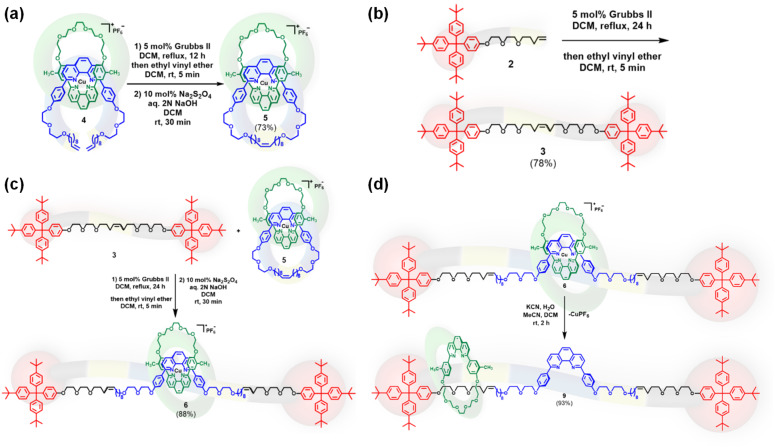
Synthesis of (a) metalated [2]catenane 5, (b) di-stoppered olefin 3, (c) ring-opening double cross-metathesis of the di-stoppered olefin 3 and a metalated [2]catenane 5 to form metalated [2]rotaxane 6, and (d) [2]rotaxane 9 from metalated [2]rotaxane 6.

The transformation (Scheme 2) requires the simultaneous ring-opening of metalated [2]catenane 5, followed by its *in situ* “insertion” into di-stoppered axle 3. Notably, ring-opening of small to medium-sized olefinic rings, coupled *in situ* with terminal or internal olefinic substrates *via* ring-opening cross-metathesis, has been successfully demonstrated in prior studies.^72–77^ Building on this precedent, we envisioned that the internal olefin functionalities present on both 5 and 3 would enable a ring-opening double cross-metathesis to afford the desired [2]rotaxane 9 upon demetalation of 6.

To this end, we selected a low-strain Sauvage-type [2]catenane^63,69,70^ as the substrate, which has been successfully demonstrated its ring-opening reactivity.^78^ The synthesis of 5 (Scheme 2a) was constructed from metalated [2]pseudorotaxane 4*via* ring-closing metathesis in the presence of 5 mol% Grubbs' second-generation catalyst,^64^ furnishing the product in 73% yield. This catenane was tactfully designed with an internal olefin on the larger macrocycle to enable further transformations *via* metathesis. In addition, two methyl groups were introduced on the phenyl rings of the smaller macrocycle 1 as spectroscopic markers to confirm its presence in the final [2]rotaxane 6.

**Fig. 1:**
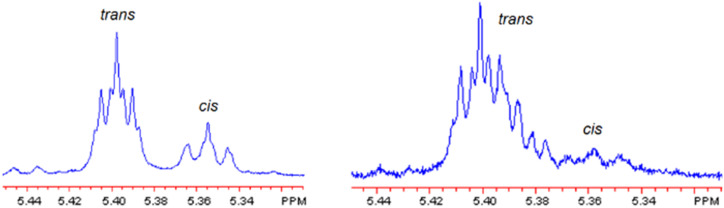
Reaction depicted and diagnostic *E*/*Z* ratio for the optimized RO-DCM that converts metalated [2]catenane 5 and di-stoppered olefin 3 into metalated [2]rotaxane 6 (Table 1, entry 10). Shown are the ^1^H NMR spectra of 3 recorded before the reaction (*E*/*Z* = 4.51 : 1.00, left) and of recovered, unreacted 3 after the reaction (*E*/*Z* = 4.98 : 1.00, right). The modest increase in the *E*/*Z* ratio indicates preferential consumption of the *Z* isomer under the optimized metathesis conditions, consistent with the high yield of 6 obtained in this trial. Integration of the diagnostic vinyl resonances was used to determine *E*/*Z* (highlighted as *trans* and *cis*).

The di-stoppered olefin 3 was synthesized (Scheme 2b) in 78% yield *via* cross-metathesis of mono-stoppered olefin 2, using the same Grubbs catalyst,^64^ ethereal linkages were introduced adjacent to the internal olefin to enhance solubility. The resulting olefinic product was obtained as a mixture of *E*/*Z* isomers in an approximate 4 : 1 ratio across four experiments, and separation of these isomers by column chromatography proved unsuccessful. During optimization studies, we found that the *cis*-isomer was preferentially consumed in the metathesis reaction, as evidenced by comparative ^1^H NMR analysis of the starting and recovered 3 (Fig. 1). This observation suggested that enriching the *cis*-isomer content by increasing the equivalents of 3 could enhance the overall yield of the target metalated [2]rotaxane 6, as proved by increasing to ten equivalents of 3 leading to a yield of 88% (Table 1, entry 10).

**Table 1:** Optimization studies of reaction condition^a^

Entry	Equiv. of 5	Equiv. of 3	Catalyst	Temp. (°C)	Time (h)	Conc. (mM)	Yield (6)^b^
1	1	1	G-II^c^	Rt	12	10	7
2	1	1	G-II	Rt	24	10	13
3	1	1	G-II	40	24	10	23
4	1	1	GH-II^d^	40	24	10	24
5	1	1	G-II	58^e^	24	10	25
6	1	1	G-II	40	24	1	11
7	1	1	G-II	40	24	50	26
8	1	2.5	G-II	40	24	10	73
9	1	5	G-II	40	24	10	81
10	1	10	G-II	40	24	10	88

a All reactions were carried out with one equiv. of 5 and 5 mol% of catalyst in dichloromethane except where noted. Equiv. of 3 was relative to the equiv. of 5.

b Isolated yield.

c G-II stands for Grubbs' second generation catalyst.

d GH-II stands for Blechert/Hoveyda–Grubbs' second generation catalyst.^63^

e Dichloroethane was used as solvent.

Initial attempts to synthesize 6 (Scheme 2c) were carried out using equimolar amounts of 5 and 3 in 10 mM dichloromethane solution with 5 mol% Grubbs II^64^ under nitrogen atmosphere at room temperature for 12 hours. The product was isolated as a dark brown solid in 7% yield (Table 1, entry 1). No mono-stoppered byproducts were detected, although both unreacted starting materials (5 and 3) were recovered. Encouraged by this result, we optimized several parameters to improve the yield of the metalated 6 (Table 1).^78–82^

Extending the reaction time to 24 hours resulted in a nearly two-fold increase in yield (13%, Table 1, entry 2), but further extension to 48 hours provided no significant additional benefit. Investigating the effect of temperature revealed that increasing the reaction temperature improved the yield substantially (Table 1, entry 3). We also explored the use of Blechert/Hoveyda–Grubbs' second-generation catalyst^63^ due to its known efficacy in cross-metathesis. While it afforded the desired product 6, the yield (24%) did not surpass that achieved under optimal conditions with Grubbs' second-generation catalyst^64^ (Table 1, entry 4), which was thus selected for all subsequent reactions.

Solvent screening demonstrated that dichloromethane at reflux temperature was optimal for this transformation. Switching to dichloroethane (DCE) and raising the temperature further did not improve the yield (Table 1, entry 5). We then examined the effect of substrate concentration. Reducing the concentration of 5 to 1 mM (Table 1, entry 6) or increasing it to 50 mM (Table 1, entry 7) both led to suboptimal yields, establishing 10 mM as the ideal concentration. As anticipated, increasing the equivalents of 3 led to the enhanced yields (Table 1, entries 8–10), culminating in an 88% yield under optimized conditions.

The identity of 6 was confirmed *via*^1^H NMR spectroscopy. Diagnostic signals included sharp aromatic peaks corresponding to the copper-complexed phenanthroline moieties and a singlet at *δ* 1.29 ppm, attributable to the *tert*-butyl groups of the stoppers. A singlet at *δ* 1.50 ppm for six methyl protons confirmed the incorporation of macrocycle 1 into the product. The olefinic region also displayed signals consistent with successful metathesis and stoppering. These spectroscopic features indicate that 6 was indeed formed through a ring-opening double cross-metathesis between 5 and 3, resulting in the symmetrical incorporation of the macrocycle within the di-stoppered axle.

**Scheme 3:**
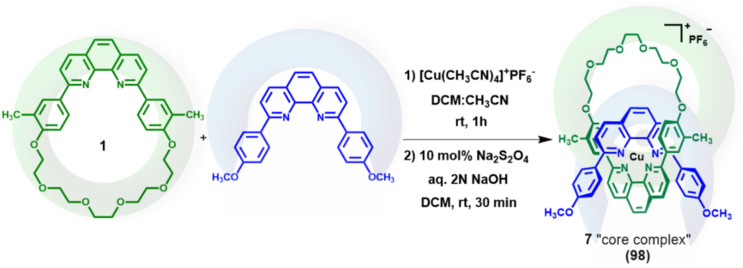
Synthesis of core complex 7.

**Fig. 2:**
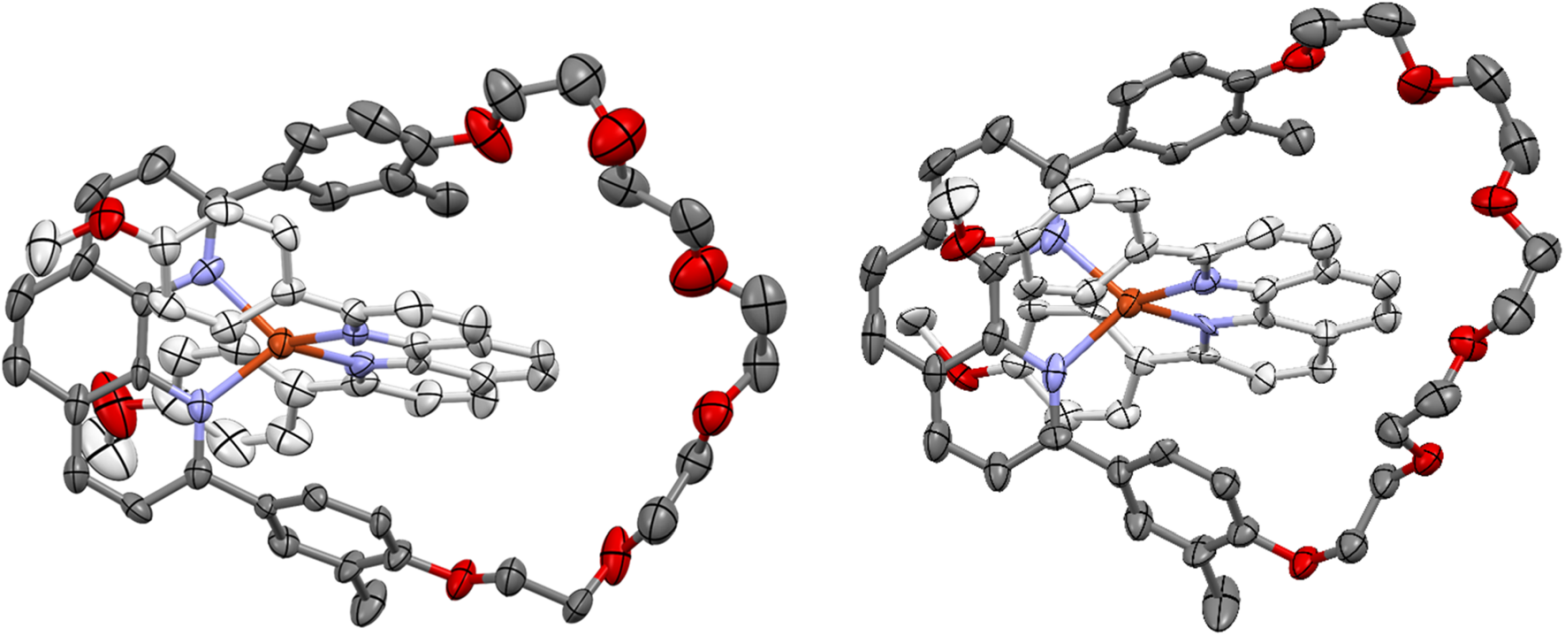
View of the solid state structure of polymorphs 1 (left) and 2 (right) of the core complex 7, hydrogen atoms, anions, and minor component of disorder were omitted for clarity, ellipsoids are drawn at the 50% probability level (Cu: orange, O: red, N: blue, C: light or dark gray).†

Although the signal at *δ* 1.50 ppm indicated the presence of the methyl groups of 1 in the product, it was not clear if they were *cis*-, *trans*-, or mixture of both *cis*- and *trans*- to each other. In order to observe the relative positions of methyl groups, the core complex 7, which is a copper(i) complex of the 2,9-di(4-methoxyphenyl)-1,10-phenanthroline ligand and macrocycle 1, was synthesized^81,82^ (Scheme 3), purified, and crystallized. Dark red prismatic and plate-shaped single crystals of the core complex were grown by slow cooling and evaporation of a methanol solution. Single-crystal X-ray diffraction showed these to be two different polymorphs of the core complex. The solid-state structures of both polymorphs (Fig. 2, S33 and S34) comprise a single [2]pseudocatenane as well as a PF_6_^−^ anion, they differ slightly in the coordination arrangement of the ligands about the Cu(i) center, and more so in the arrangement of the glycol chains (Fig. S35).††Crystal data for polymorph 1 of complex 7: C_62_H_58_CuF_6_N_4_O_8_P, *M* = 1195.63, triclinic, *P*1̄, *a* = 14.7809(14), *b* = 14.8375(16), *c* = 15.4905(18) Å, *α* = 115.129(11), *β* = 114.546(10), *γ* = 92.324(8)°, *V* = 2698.9(6) Å^3^, *Z* = 2, *T* = 173(2) K, 6604 unique reflections. Refinement of 801 parameters converged at final *R*_1_ [*I* > 2*σ*(*I*)] of 0.0754, *wR*_2_ (all data) of 0.1548. Crystal data for polymorph 2: C_62_H_58_CuF_6_N_4_O_8_P, *M* = 1195.63, monoclinic, *P*2_1_/*c*, *a* = 16.7265(8), *b* = 7.8498(4), *c* = 43.035(2) Å, *β* = 96.345(5)°, *V* = 5615.8(5) Å^3^, *Z* = 4, *T* = 173(2) K, 6867 unique reflections. Refinement of 834 parameters converged at final *R*_1_ [*I* > 2*σ*(*I*)] of 0.1472, *wR*_2_ (all data) of 0.3205. See Table S1 in the SI for more details. The Cu–N bond lengths of the first polymorph are typical [2.018(9) to 2.059(8) Å] of such complexes, those of the second polymorph slightly longer [2.034(10) to 2.094 Å], and, although the N–Cu–N angles show significant distortion from ideal tetrahedral geometry [82.0(3) to 134.7(3)° and 83.5(4) to 135.9(4)° for polymorphs 1 and 2 (Fig. S33 and S34), respectively], this is in agreement with previously reported examples.^83,84^ These distortions enable a more compact geometric arrangement of the individual complexes, with a number of intramolecular π⋯π interaction taking place [centroid⋯centroid distances 3.680(6) to 3.919(5) Å and 3.707(8) to 3.968(8) Å for the two polymorphs]. Efficient packing of the complexes in both polymorphs is aided by weak hydrogen bonds between both aliphatic and aromatic hydrogens and methoxy and glycol oxygens, as well as fluorides of the PF_6_^−^ anions (CH⋯acceptor distances 2.05 to 2.53, and 2.34 to 2.52 Å). Neither polymorph displayed intermolecular CH⋯π interactions, and only polymorph 2 showed any intermolecular π⋯π interactions, occurring between benzene rings of the two different ligands on adjacent molecules, at a centroid⋯centroid distance of 3.600(5) Å. It is perhaps surprising that only one methyl group, in polymorph 2, forms an intermolecular interaction with nearby aromatic systems, however, this is likely due to the benzene-ring systems they are bound to being oriented to take part in intramolecular π⋯π interactions.

The two methyl groups on macrocycle 1 allow for a few limiting stereoisomeric structures^82^ for the core complex 7. These groups may be oriented in the same(*cis*)/opposite(*trans*) or *cis* and *trans* directions, which allow a *cis*-, or *trans*-isomer or an isomer with methyl groups in *cis* and *trans* directions. However, the methyl groups in both polymorphic structures of 7 reveal a solid-state preference for a *cis*-isomer (Fig. 2, S33 and S34). Similarly, as it is mentioned in the literature,^80^ the presence of the methyl groups on the ligands of homoleptic copper(i) complexes of 2,9-di(4-methoxy-3-methylphenyl)-1,10-phenanthroline and 2,9-di(4-hydroxy-3-methylphenyl)-1,10-phenanthroline ligands also allows for numerous limiting stereoisomeric structures for the respective complexes.^80,82^ However, in the X-ray crystal structure of the copper(i) complex of 2,9-di(4-methoxy-3-methylphenyl)-1,10-phenanthroline ligand, the methyl groups were found to be a pair of enantiomeric *trans*, *trans*-atropisomers. Comparing ^1^H NMR spectra of the copper(i) complex of 2,9-di(4-methoxy-3-methylphenyl)-1,10-phenanthroline ligand and that of complex 7 (Fig. S21 and S22, S25–S28), neither complex showed splitting or multiple signals for the *trans* or *cis* methyls. Even in variable temperature (VT) ^1^H NMR studies (Fig. S23 and S24), no additional signals or even signal broadening resulting from the methyl protons in 7 were observed.

**Fig. 3:**
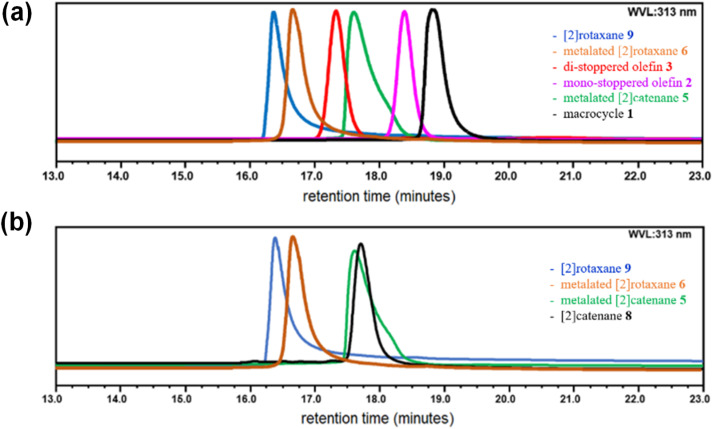
Combined size-exclusion chromatograms of (a) macrocycle 1 (black), mono-stoppered olefin 2 (purple), di-stoppered olefin 3 (red), metalated [2]catenane 5 (green), metalated [2]rotaxane 6 (brown), and [2]rotaxane 9 (blue), and (b) metalated [2]catenane 5 (green), [2]catenane 8 (black) metalated [2]rotaxane 6 (brown), and [2]rotaxane 9 (blue).

Following synthesis of the metalated [2]rotaxane 6 (Scheme 2c), demetalation (Scheme 2d) yielded [2]rotaxane 9. Its interlocked structure was confirmed *via*^1^H NMR (Fig. S31) and analytical size-exclusion chromatography (SEC) (Fig. S44). Demetalation was achieved by treating 6 with aqueous KCN in acetonitrile–dichloromethane. After completion, ethyl vinyl ether (2 drops in 0.1–0.2 mL dichloromethane) was added, stirred for 5 min at room temperature. The disappearance of the dark brown color indicated demetalation. Solvents were evaporated to afford crude 9 (Scheme 2d), which was purified by preparative SEC and analyzed *via* analytical SEC (Fig. S44), which confirmed formation of [2]rotaxane 9. Retention times were 18.8, 18.3, 17.3, 17.6, 16.4, 17.8, and 16.6 min for macrocycle 1, mono-stoppered olefin 2, di-stoppered olefin 3, metalated [2]catenane 5, metalated [2]rotaxane 6, [2]catenane 8, and [2]rotaxane 9, respectively (Fig. 3a and b). [2]Catenane 8 exhibited a sharp peak with tailing at 17.8 min (Fig. 3b, black), while 5 showed a sharp peak at 17.6 min (Fig. 3b, green). In contrast, metalated [2]rotaxane 6 (Fig. 3b, brown) and [2]rotaxane 9 (Fig. 3b, blue) exhibited sharp peaks at 16.4 and 16.6 min, respectively. The absence of a 17.8 min peak confirmed no contamination of 9 with 8. To examine potential dethreading of macrocycle 1 from 9 upon demetalation, the conventional heating experiments^42^ were conducted in dichloromethane (30–40 °C) and dimethyl-sulfoxide (80–120 °C) for 12–48 h. No dethreading was detected.

## Conclusion

In conclusion, we demonstrated a novel synthesis in which a thermodynamically steered ring-opening double cross-metathesis between a Cu(i)-templated [2]catenane 5 and a di-stoppered axle 3 delivered the target metalated [2]rotaxane 6 in high yield of 88% which was quantitatively converted to [2]rotaxane 9*via* demetalation, while suppressing irrecoverable, non-interlocked byproducts. By exploiting dynamic covalent chemistry under equilibrium control, the system self-edits to the most stable interlocked architecture—circumventing inefficiencies that commonly afflict clipping/threading/slipping and related active-template routes.^62,85^ Mechanistically, the approach capitalizes on well-established Ru-carbene metathesis manifolds (RO(C)M/CM) that engage internal olefins under mild conditions,^65,66^ providing a robust and atom-economical bond-reorganization platform. The choice of a low-strain Sauvage-type catenane^68,86^ ensures metathesis addressability without sacrificing mechanical integrity, consistent with the copper-templated foundations of catenane chemistry. Spectroscopic and SEC analyses authenticate the interlocked product, and demetalation preserves the mechanical bond with no detectable dethreading under thermal stress—underscoring the kinetic persistence of the architecture. By contrast, coordination-driven, ligand-bound catenanes can be formed reversibly from rotaxanes (bis-Zn-porphyrin ‘tweezers’ + DABCO or 4,4′-bipyridine).^87^ Beyond furnishing a concise entry to [2]rotaxanes, the strategy establishes a general, dynamic ring-chain equilibration blueprint that is readily extendable to sequence-defined mechanically interlocked polymers and topologies previously deemed synthetically inaccessible.^88,89^ Thus, this methodology highlights the potential of metalated [2]rotaxane 6 as a versatile intermediate for constructing oligorotaxanes *via* partial depolymerization of polypseudorotaxanes derived from 5. Future efforts will explore the application of 6 in accessing oligo- and polyrotaxanes, expanding its utility in supramolecular polymer synthesis.

## Experimental section

### Experimental: general methods

#### Material and instrumentation

Anhydrous dichloromethane (DCM) and acetonitrile were separately distilled over CaH_2_ under nitrogen. Diethyl ether and tetrahydrofuran (THF) were separately distilled over Na/benzophenone under nitrogen. *N*,*N*-Dimethylformamide (DMF) was passed through FC 15 packed columns within a manual solvent purification system from MBraun. 2,9-Di(4-hydroxyphenyl)-1,10-phenanthroline and 2,9-di(4-methoxyphenyl)-1,10-phenanthroline,^63^ dialcohol,^70^ α,ω-diolefin,^78^ 2,9-di(4-methoxy-3-methylphenyl)-1,10-phenanthroline and 2,9-di(4-hydroxy-3-methylphenyl)-1,10-phenanthroline,^90^ tris(*p-tert*-butylphenyl)methanol, 4-tris(*p-tert*-butylphenyl)phenol and mono-stoppered linear alcohol,^91,92^ 10-bromo-1-decene,^93,94^ 2-methylanisole,^95^ 4-bromo-2-methylanisole,^96^ Grubbs' first generation catalyst for the synthesis of II,^97,98^ and Grubbs' second generation catalyst^64^ were prepared using literature procedures. ^1^H NMR and ^13^C NMR spectra were recorded on a Varian Mercury Plus 300 or a Varian Unity Inova 500 spectrometer. For ^1^H NMR spectra, tetramethylsilane (TMS, *δ* = 0.00) or the residual protic solvent peak (for CD_2_Cl_2_, *δ* = 5.32, for CDCl_3_, *δ* = 7.26, and for DMSO-*d*_6_, *δ* = 2.50) served as a shift reference. Coupling constants, *J*, are reported in hertz (Hz). For ^13^C NMR spectra which were obtained with ^1^H decoupling, CDCl_3_ (*δ* = 77.16), CD_2_Cl_2_ (*δ* = 53.84) and (CD_3_)_2_SO (*δ* = 39.52) were used as a shift reference. HMQC spectra were recorded on a Varian Unity Inova 500 spectrometer. High-resolution ESI mass spectrometry was performed on an Exactive-Orbitrap mass spectrometer at Texas Tech University (Lubbock, TX), and a Micromass Q-Tof Ultima spectrometer in the University of Illinois Mass Spectrometry Laboratories (Urbana-Champaign, IL). Elemental analyses were performed on a PerkinElmer 2400 Series II Elemental Analyzer at the Texas Tech University (Lubbock, TX), and by Columbia Analytical Service, Inc. (Tucson, AZ). Analytical size-exclusion chromatography (SEC) was performed on a system which consisted of a Dionex P680 pump and a Dionex UVD 170U/340U UV-Vis detector. Two Waters Styragel HR 4E (7.8 × 300 mm) columns were used in series and maintained at 24 °C. Chloroform (CHCl_3_) was used as the mobile phase at a flow rate of 1.0 mL min^−1^. Detection was performed at a wavelength of 254 or 313 nm. Molecular weights were estimated (by SEC) by calibration with monodisperse (PDI < 1.1) polystyrene standards, retention time (min)/*M*_w_: 18.3/6.0 × 10^2^, 18.0/1.0 × 10^3^, 16.2/4.0 × 10^3^, 14.2/2.0 × 10^4^, 13.4/5.0 × 10^4^, 12.6/1.0 × 10^5^ (Polysciences, Inc.). Preparative SEC was performed using Bio-Beads^®^ S-X1 Beads (200–400 mesh) with DCM (CH_2_Cl_2_) as eluent. Flash chromatography was performed using Silicycle UltraPure Flash Silica Gel (60 Å, 40–63 µm). Thin layer chromatography (TLC) was performed using EMD HPTLC plates, silica gel 60, F_254_. All reaction vessels were flame-dried under vacuum and filled with nitrogen prior to use. All reactions were performed under a nitrogen atmosphere as a routine practice, not as an essential requirement.

### Synthetic procedures and structural determination data

The detailed synthetic procedures and structural characterization data for the intermediates and desired compounds are presented in the SI. Some important details and structural determination data for the intermediates and desired product(s) are presented below.

#### Dimethylated macrocycle 1

Obtained the dimethylated macrocycle 1 (12.5 g, 82%) as a bright yellow solid, m.p. 153.8–155.1 °C; ^1^H NMR (500 MHz, CDCl_3_) *δ* 8.37 (dd, 2.0, 2.0 Hz, 2H), 8.26–8.24 (m, 4H), 8.07 (d, *J* = 8.0 Hz, 2H), 7.74 (s, 2H), 7.15 (d, *J* = 8.5 Hz, 2H), 4.34 (t, *J* = 5.0 Hz, 5.5 Hz, 4H), 3.84 (t, *J* = 5.5 Hz, 5.0 Hz, 4H), 3.75–3.69 (m, 12H), 2.42 (s, 6H); ^13^C NMR (125 MHz, CDCl_3_) 158.37, 156.40, 145.88, 136.53, 132.35, 130.14, 127.64, 127.27, 126.51, 125.41, 119.04, 112.95, 71.02, 70.66, 70.56, 69.52, 68.28, 16.69; HRMS (ESI) calcd for C_36_H_39_N_2_O_6_ [M + H]^+^*m*/*z* 595.2803, found *m*/*z* 595.2800; anal. calcd for C_36_H_38_N_2_O_6_: C, 72.71; H, 6.44; N, 4.71; found: C, 72.33; H, 6.51; N, 4.66.

#### Mono-stoppered olefin 2

Obtained the mono-stoppered olefin 2 (8.81 g, 79%) as a white solid, m.p. 151.4–152.3 °C; ^1^H NMR (500 MHz, CDCl_3_) *δ* 7.23 (d, *J* = 8.5 Hz, 6H), 7.07 (d, *J* = 8.5 Hz, 8H), 6.78 (d, *J* = 9.0 Hz, 2H), 5.85–5.76 (m, 1H), 5.03–4.93 (m, 2H), 4.11 (t, *J* = 4.5 Hz, 5.5 Hz, 2H), 3.85 (t, *J* = 5.0 Hz, 2H), 3.72–3.70 (m, 2H), 3.64–3.60 (m, 2H), 3.48 (t, *J* = 6.5 Hz, 7.0 Hz, 2H), 2.13–2.08 (m, 2H), 1.71–1.66 (m, 2H), 1.30 (s, 27H); ^13^C NMR (125 MHz, CDCl_3_) *δ* 156.56, 148.27, 144.12, 139.72, 138.27, 132.20, 130.71, 124.01, 114.69, 113.06, 70.82, 70.76, 70.15, 69.78, 67.22, 63.03, 34.28, 31.37, 30.22, 28.76; HRMS (ESI) calcd for C_46_H_61_O_3_ [M + H]^+^*m*/*z* 661.4615, found *m*/*z* 661.4618; anal. calcd for C_46_H_60_O_3_: C, 83.59; H, 9.15; found: C, 83.61; H, 8.85.

#### Di-stoppered olefin 3

Obtained the di-stoppered olefin 3 (mixture of *cis*- and *trans*-) as a white solid (121.5 mg, 78%), m.p. 165.9–168.8 °C; ^1^H NMR (500 MHz, CDCl_3_) *δ* 7.23 (d, *J* = 8.5 Hz, 12H), 7.08 (d, *J* = 8.5 Hz, 16H), 6.77 (d, *J* = 9.0 Hz, 4H), 5.50–5.35 (m, 2H), 4.11 (t, *J* = 5.0 Hz, 4H), 3.85 (t, *J* = 5.0 Hz, 4H), 3.72–3.70 (m, 4H), 3.62–3.59 (m, 4H), 3.49–3.44 (m, 4H), 2.11–2.02 (m, 4H), 1.67–1.61 (m, 4H), 1.30 (s, 54H); ^13^C NMR (125 MHz, CDCl_3_) *δ* 156.56, 148.27, 144.12, 139.70, 132.19, 130.71(*trans*), 130.01(*cis*), 124.01, 114.69, 113.05, 70.85, 70.80, 70.13, 69.79, 67.20, 63.03, 34.27, 31.37, 29.41, 28.99; HRMS (ESI) calcd for C_90_H_116_NaO_6_ [M + Na]^+^*m*/*z* 1315.8664, found *m*/*z* 1315.8628; anal. calcd for C_90_H_116_O_6_: C, 83.54; H, 9.04; found: C, 83.32; H, 9.09.

#### Metalated [2]pseudorotaxane 4

Obtained the metalated [2]pseudorotaxane 4 as a red glassy solid (647 mg, 98%). ^1^H NMR (500 MHz, CDCl_3_) *δ* 8.63 (d, *J* = 8.5 Hz, 2H), 8.47 (d, *J* = 8.5 Hz, 2H), 8.20 (s, 2H), 8.02 (s, 2H), 7.86 (d, *J* = 8.5 Hz, 2H), 7.78 (d, *J* = 8.5 Hz, 2H), 7.47 (d, *J* = 8.0 Hz, 4H), 7.15 (d, *J* = 8.0 Hz, 2H), 6.95 (s, 2H), 6.08 (d, *J* = 8.5 Hz, 4H), 5.81 (d, *J* = 8.5 Hz, 2H), 5.79–5.72 (m, 2H), 4.96–4.87 (m, 4H), 3.86 (s, 4H), 3.78 (s, 8H), 3.74–3.70 (m, 8H), 3.65–3.62 (m, 12H), 3.59–3.57 (m, 4H), 3.48 (t, *J* = 7.0 Hz, 4H), 2.01–1.97 (m, 4H), 1.62–1.57 (m, 4H), 1.48 (s, 6H), 1.38–1.21 (m, 20H); ^13^C NMR (125 MHz, CDCl_3_) *δ* 159.26, 156.98, 156.70, 155.46, 143.20, 143.18, 139.05, 137.58, 136.76, 132.00, 131.06, 130.04, 128.96, 127.91, 127.65, 126.94, 126.30, 126.04, 125.62, 123.90, 123.84, 114.02, 112.90, 109.18, 71.46, 71.02, 70.77, 70.77, 70.69, 69.99, 69.33, 69.25, 67.35, 67.26, 33.65, 29.56, 29.31, 29.31, 28.94, 28.77, 25.98, 15.59; HRMS (ESI) calcd for C_88_H_106_CuN_4_O_12_ [M − PF_6_]^+^*m*/*z* 1473.7098, found *m*/*z* 1473.7084; anal. calcd for C_88_H_106_CuF_6_N_4_O_12_P: C, 65.23; H, 6.59; N, 3.46; found: C, 64.86; H, 6.36; N, 3.38.

#### Metalated [2]catenane 5

Obtained the metalated [2]catenane 5 as a red glassy solid (358.7 mg, 73%). ^1^H NMR (500 MHz, CDCl_3_) *δ* 8.64 (d, *J* = 8.5 Hz, 2H), 8.48 (d, *J* = 8.0 Hz, 2H), 8.22 (s, 2H), 8.05 (s, 2H), 7.88 (d, *J* = 8.0 Hz, 2H), 7.80 (d, *J* = 8.0 Hz, 2H), 7.48 (d, *J* = 8.0 Hz, 4H), 7.18 (d, *J* = 8.0 Hz, 2H), 6.97 (s, 2H), 6.09 (d, *J* = 8.5 Hz, 4H), 5.83 (d, *J* = 8.5 Hz, 2H), 5.37–5.30 (m, 2H) (*trans*- and *cis*-), 3.88 (s, 4H), 3.78 (s, 8H), 3.75–3.73 (m, 8H), 3.71–3.69 (m, 4H), 3.66–3.65 (m, 8H), 3.61–3.59 (m, 4H), 3.54 (t, 4H), 1.98–1.90 (m, 4H), 1.67–1.62 (m, 4H), 1.50 (s, 6H), 1.38–1.25 (m, 20H); ^13^C NMR (125 MHz, CDCl_3_) *δ* 159.47, 157.24, 156.96, 155.77, 143.49, 143.47, 137.85, 137.04, 132.26, 131.39, 130.57 (*trans*), 130.33, 130.06 (*cis*), 129.25, 128.18, 127.95, 127.20, 126.61, 126.41, 125.87, 124.18, 124.12, 113.12, 109.43, 71.77, 71.31, 71.08, 71.05, 71.05, 70.43, 69.65, 69.54, 67.57, 67.57, 32.46, 29.80, 29.41, 29.40, 29.38, 28.71, 26.19, 15.88; HRMS (ESI) calcd for C_86_H_102_CuN_4_O_12_ [M − PF_6_]^+^*m*/*z* 1445.6785, found *m*/*z* 1445.6782; anal. calcd for C_86_H_102_CuF_6_N_4_O_12_P: C, 64.87; H, 6.46; N, 3.52; found: C, 64.63; H, 6.17; N, 3.38.

#### Metalated [2]rotaxane 6

Obtained the metalated [2]rotaxane 6 as a dark brown solid (48.1 mg, 88%). 1H NMR (400 MHz, CDCl_3_) *δ* 8.64 (d, *J* = 8.2 Hz, 2H), 8.47 (d, *J* = 8.2 Hz, 2H), 8.22 (s, 2H), 8.02 (s, 2H), 7.88 (d, *J* = 8.2 Hz, 2H), 7.80 (d, *J* = 8.2 Hz, 2H), 7.48 (d, *J* = 8.7 Hz, 4H), 7.23–7.15 (m, 14H), 7.07 (d, *J* = 8.7 Hz, 16H), 6.96 (s, 2H), 6.77 (d, *J* = 8.7 Hz, 4H), 6.10 (d, *J* = 8.7 Hz, 4H), 5.82 (d, *J* = 8.2 Hz, 2H), 5.46–5.31 (m, 4H), 4.10 (t, *J* = 4.6 Hz, 4H), 3.88–3.43 (m, 56H), 2.04–1.74 (m, 8H), 1.69–1.59 (m, 8H), 1.50 (s, 6H), 1.38–1.26 (m, 74H); 13C NMR (100 MHz, CDCl_3_) *δ* 159.49, 157.18, 156.96, 156.68, 155.74, 148.41, 144.26, 143.46, 139.85, 137.77, 137.01, 132.34, 132.34, 132.27, 131.30, 131.05, 131.00, 130.90, 130.84, 130.84 (*trans*), 130.30 (*cis*), 129.55, 129.20, 128.14, 127.88, 127.15, 126.58, 126.28, 125.82, 124.16, 124.16, 113.18, 109.30, 71.78, 71.29, 71.04, 71.04, 70.94, 70.94, 70.22, 70.21, 69.91, 69.57, 69.56, 67.51, 67.50, 67.32, 63.16, 34.43, 32.72, 31.52, 31.52, 29.81, 29.80, 29.73, 29.62, 29.61, 29.29, 29.15, 26.25, 15.89; HRMS (MALDI) calcd for C_176_H_219_CuN_4_O_18_ [M + H-PF6]^+^*m*/*z* 2739.5630, found *m*/*z* 2739.5598; anal. calcd for C_176_H_218_CuF_6_N_4_O_18_P: C, 73.24; H, 7.61; N, 1.94; found: C, 73.57; H, 7.24; N, 1.99.

#### Core complex 7

Obtained the core complex 7 (400 mg, 98%) as a red glassy solid, m.p. 252.4–253.0 °C; ^1^H NMR (500 MHz, CDCl_3_) *δ* 8.64 (d, *J* = 8.5 Hz, 2H), 8.46 (d, *J* = 8.0 Hz, 2H), 8.22 (s, 2H), 8.00 (s, 2H), 7.89 (d, *J* = 8.0 Hz, 2H), 7.80 (d, *J* = 8.0 Hz, 2H), 7.51 (d, *J* = 8.5 Hz, 4H), 7.18 (d, *J* = 8.5 Hz, 2H), 6.95 (s, 2H), 6.08 (d, *J* = 8.5 Hz, 4H), 5.81 (d, *J* = 8.0 Hz, 2H), 3.88 (s, 4H), 3.76–3.74 (m, 4H), 3.67–3.64 (m, 8H), 3.61–3.59 (m, 4H), 3.52 (s, 6H), 1.51 (s, 6H); ^13^C NMR (125 MHz, CDCl_3_) *δ* 160.28, 157.24, 157.06, 155.74, 143.46, 137.81, 136.86, 132.22, 131.20, 130.30, 129.28, 129.28, 128.16, 127.84, 127.20, 126.56, 126.09, 125.85, 124.19, 112.58, 112.58, 109.38, 71.31, 71.06, 71.06, 69.52, 67.52, 55.38, 15.88 ; HRMS (ESI) calcd for C_62_H_58_CuN_4_O_8_ [M − PF_6_]^+^*m*/*z* 1049.3545, found *m*/*z* 1049.3521; anal. calcd for C_62_H_58_CuF_6_N_4_O_8_P: C, 62.28; H, 4.89; N, 4.69; found: C, 62.35; H, 4.69; N, 4.62.

#### Demetalated [2]catenane 8

Obtained the demetalated [2]catenane 8 which was further analyzed by analytical SEC; ^1^H NMR (400 MHz, CDCl_3_) *δ* 8.97 (dd, *J* = 2.3 Hz, 2H), 8.42 (d, *J* = 8.7 Hz, 4H), 8.24 (d, *J* = 8.3 Hz, 2H), 8.19 (d, *J* = 8.7 Hz, 2H), 8.08 (s, 2H), 8.06 (s, 2H), 7.77 (s, 2H), 7.71 (d, *J* = 10.1 Hz, 4H), 7.25–7.17 (m, 6H), 5.41–5.34 (m, 2H) (*trans*- and *cis*-), 4.28–4.26 (m, 4H), 4.20–4.17 (m, 4H), 3.97–3.90 (m, 4H), 3.77–3.67 (m, 20H), 3.59–3.53 (m, 4H), 3.39–3.34 (m, 4H), 2.27 (s, 6H), 2.01–1.96 (m, 4H), 1.51–1.48 (m, 4H), 1.31–1.23 (m, 20H); ^13^C NMR (100 MHz, CDCl_3_) *δ* 160.27, 158.21, 156.47, 156.47, 146.30, 146.10, 136.70, 136.50, 132.10, 132.03, 130.53 (*trans*), 130.05 (*cis*), 129.10, 129.10, 128.77, 128.28, 127.53, 127.37, 126.02, 125.60, 125.46, 119.44, 119.06, 115.25, 112.95, 71.58, 71.12, 71.00, 70.89, 70.63, 70.30, 70.06, 68.50, 67.72, 66.22, 32.61, 29.71, 29.55, 29.44, 29.32, 28.97, 26.18, 16.63; HRMS (ESI) calcd for C_86_H_102_N_4_O_12_ [M + H]^+^*m*/*z* 1382.7489, found *m*/*z* 1382.7393.

#### Demetalated [2]rotaxane 9

Obtained the demetalated [2]rotaxane 9 which was further analyzed by analytical SEC; ^1^H NMR (400 MHz, CDCl_3_) *δ* 8.92 (d, *J* = 8.7 Hz, 2H), 8.40 (d, *J* = 8.2 Hz, 4H), 8.21 (d, *J* = 8.2 Hz, 4H), 8.07–8.03 (m, 4H), 7.75 (s, 2H), 7.70 (s, 4H), 7.23–7.15 (m, 18H), 7.06 (d, *J* = 8.7 Hz, 16H), 6.83–6.75 (m, 4H), 5.38–5.25 (m, 4H), 4.27–3.35 (m, 60H), 2.27 (s, 6H), 2.04–1.89 (m, 8H), 1.68–1.44 (m, 8H), 1.37–1.25 (m, 74H); ^13^C NMR (100 MHz, CDCl_3_) *δ* 160.27, 158.22, 156.73, 156.50, 148.35, 148.35, 146.15, 144.31, 139.74, 136.82, 136.50, 132.33, 130.85, 130.85 (*trans*), 129.56, 129.11 (*cis*), 128.78, 128.77, 128.75, 128.24, 128.21, 128.19, 127.62, 127.40, 125.67, 125.49, 124.15, 124.15, 119.49, 115.16, 113.38, 113.16, 112.96, 71.72, 71.02, 71.02, 70.90, 70.82, 70.58, 70.24, 70.24, 69.98, 69.96, 68.48, 67.69, 67.39, 66.27, 63.18, 34.42, 34.41, 32.75, 32.07, 31.51, 29.85, 29.80, 29.63, 29.76, 26.24, 22.85, 16.67, 14.28; HRMS (MALDI) calcd for C_176_H_219_N_4_O_18_ [M + H]^+^*m*/*z* 2376.6339, found *m*/*z* 2376.3344.

## Author contributions

This manuscript and its associated materials were prepared as follows: experimental design was conducted by M. M. C., M. F. M., and A. M.; chemical synthesis was performed by M. M. C.; manuscript drafting was carried out by M. M. C. and M. F. M.; spectral data analysis was performed by M. M. C. and M. F. M.; crystallographic studies and structural analysis were undertaken by D. B. C. and M. M. C.; reviewing and editing were completed by M. M. C., M. F. M., D. B. C., Z. Y. and V. G. F.; visual content creation (images, figures, and graphical abstract) was handled by V. G. F. and M. M. C.; additional supporting details were prepared by M. M. C. All data were generated in-house, and no paper mill was used. All authors agree to be accountable for all aspects of work ensuring integrity and accuracy.

## Conflicts of interest

The authors declare no competing financial interest or personal relationships that may have affected their work.

## Supplementary Material

RA-015-D5RA07142A-s001

RA-015-D5RA07142A-s002

## Data Availability

CCDC 967317 and 2469972 (polymorphs (1 and 2) of the core complex 7) contain the supplementary crystallographic data for this paper.^99*a*,*b*^ The data supporting this article have been included as part of the supplementary information (SI). Supplementary information: synthetic procedures and structural characterization data for the intermediates and desired compounds. See DOI: https://doi.org/10.1039/d5ra07142a.
