# Supplementary material for: Rotaxane synthesis via a dynamic [2]catenane-ring-opening, axle-cleaving double cross metathesis
Source: RSC Adv. 2025 Dec 1;15(55):47117–27. doi: 10.1039/d5ra07142a (PMC12666508; doi:10.1039/d5ra07142a)
Supplement: RA-015-D5RA07142A-s001 [file RA-015-D5RA07142A-s001.pdf]

Supplementary Information (SI) for RSC Advances.
This journal is © The Royal Society of Chemistry 2025

Supporting Information
for

Rotaxane Synthesis *via* a Dynamic [2]Catenane-Ring-Opening, Axle-Cleaving Double Cross Metathesis

M. Mustafa Cetin[*a,f,g], Arindam Mazumdar[b], David B. Cordes[c], Vahap Gazi Fidan[d], Zhen Yang[e] and Michael F. Mayer[*b]

[a]*Department of Chemistry, İstinye University, Sarıyer, İstanbul 34396, TÜRKİYE*
[b]*Department of Chemistry and Biochemistry, Texas Tech University, MS-41062, Lubbock, TX 79409, USA*
[c]*EaStCHEM School of Chemistry, University of St. Andrews, St. Andrews, KY16 9ST, UK*
[d]*Department of Chemical and Biological Engineering, Koç University, Sarıyer, İstanbul 34450, TÜRKİYE*
[e]*Strait Institute of Flexible Electronics (SIFE, Future Technologies), Fujian Key Laboratory of Flexible Electronics, Fujian Normal University and Strait Laboratory of Flexible Electronics (SLoFE), Fuzhou, 350117, CHINA*
[f]*Synthetic Organic and Polymer Research (SOPR) Laboratory, İstinye University, Sarıyer, İstanbul 34396, TÜRKİYE*
[g]*Clean Energy Research Center (TEAM), İstinye University, Sarıyer, İstanbul 34396, TÜRKİYE*

*E-mail: mf.mayer@ttu.edu and mustafa.cetin@istinye.edu.tr

Table of Contents

**Section A.**	**Materials / General Methods / Instrumentation**	**S2**
**Section B.**	**Synthetic Protocols**	**S4**
**Section C.**	**NMR Spectroscopy**	**S15**
**Section D.**	**Crystallographic Characterization**	**S38**
**Section E.**	**Size-Exclusion Chromatograms (SECs)**	**S43**
**Section F.**	**Additional Notes**	**S46**
**Section G.**	**References**	**S48**

Section A. Materials / General Methods / Instrumentation

Reagents:

Anhydrous dichloromethane (DCM) and acetonitrile were separately distilled over CaH_2 under nitrogen. Diethyl ether and tetrahydrofuran (THF) were separately distilled over Na/benzophenone under nitrogen. *N,N*-Dimethylformamide (DMF) was passed through FC 15 packed columns within a manual solvent purification system from MBraun. 2,9-di(4-hydroxyphenyl)-1,10-phenanthroline and 2,9-di(4-methoxyphenyl)-1,10-phenanthroline,[1] dialcohol,[2] α,ω-diolefin,[3] 2,9-di(4-methoxy-3-methylphenyl)-1,10-phenanthroline and 2,9-di(4-hydroxy-3-methylphenyl)-1,10-phenanthroline,[4] tris(*p-tert*-butylphenyl)methanol, 4-tris(*p-tert*-butylphenyl)phenol and mono-stoppered linear alcohol,[5, 6] 10-bromo-1-decene,[7, 8] 2-methylanisole,[9] 4-bromo-2-methylanisole,[10] Grubbs' first generation catalyst for the synthesis of II,[11, 12] and Grubbs' second generation catalyst[13] were prepared using literature procedures.

Methods: ^{1}H NMR and ^{13}C NMR spectra were recorded on a Varian Mercury Plus 300 or a Varian Unity Inova 500 spectrometer. For ^{1}H NMR spectra, tetramethylsilane (TMS, δ = 0.00) or the residual protic solvent peak (for CD_2Cl_2, δ = 5.32, for $CDCl_3$, δ = 7.26, and for DMSO-d_6, δ = 2.50) served as a shift reference. Coupling constants, *J*, are reported in hertz. For ^{13}C NMR spectra which were obtained with ^{1}H decoupling, $CDCl_3$ (δ = 77.16), CD_2Cl_2 (δ = 53.84) and $(CD_3)_2SO$ (δ = 39.52) were used as a shift reference. HMQC spectra were recorded on a Varian Unity Inova 500 spectrometer.

High-resolution ESI mass spectrometry was performed on an Exactive-Orbitrap mass spectrometer at Texas Tech University (Lubbock, TX), and a Micromass Q-Tof Ultima spectrometer in the University of Illinois Mass Spectrometry Laboratories (Urbana-Champaign, IL).

Elemental analyses were performed on a PerkinElmer 2400 Series II Elemental Analyzer at the Texas Tech University (Lubbock, TX), and by Columbia Analytical Service, Inc. (Tucson, AZ).

Analytical size-exclusion chromatography (SEC) was performed on a system which consisted of a Dionex P680 pump and a Dionex UVD 170U/340U UV-Vis detector. Two Waters Styragel HR 4E (7.8 x 300 mm) columns were used in series and maintained at 24 °C. Chloroform ($CHCl_3$) was used as the mobile phase at a flow rate of 1.0 mL/min. Detection was performed at a wavelength of 254 or 313 nm. Molecular weights were estimated (by SEC) by calibration with monodisperse (PDI < 1.1) polystyrene standards, retention time (min)/M_w: 18.3/6.0 × 10^2, 18.0/1.0 × 10^3, 16.2/4.0 × 10^3, 14.2/2.0 × 10^4, 13.4/5.0 × 10^4, 12.6/1.0 × 10^5 (Polysciences, Inc.).

Preparative SEC was performed using Bio-Beads® S-X1 Beads (200-400 Mesh) with DCM (CH_2Cl_2) as eluent.

Flash chromatography was performed using Silicycle UltraPure Flash Silica Gel (60 Å, 40-63 μm). Thin layer chromatography (TLC) was performed using EMD HPTLC plates, silica gel 60, F_{254}.

All reaction vessels were flame-dried under vacuum and filled with nitrogen prior to use. All reactions were performed under a nitrogen atmosphere as a routine practice, not as an essential requirement.

Section B. Synthetic Protocols

The detailed synthetic procedures and the structural characterization data for the intermediates and target compounds are presented below.

Synthesis of dimethylated macrocycle 1

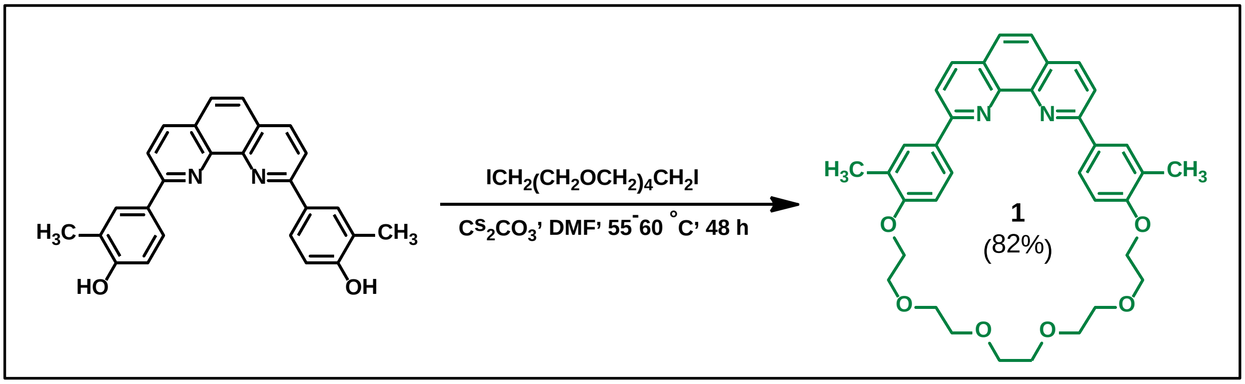

Scheme S1. Synthesis of compound **1**.

In a flame-dried round-bottom flask, a mixture of the dimethylated diphenol phenanthroline precursor (10.0 g, 25.4 mmol) and 1,14-diiodo-3,6,9,12-tetraoxatetradecane (12.4 g, 27.0 mmol) in DMF (400 mL) was added drop wise within 24 hours under efficient stirring to a nitrogen flushed suspension of Cs_2CO_3 (25.6 g, 72.6 mmol) in DMF (150 mL) kept at 55-60 °C. At the end of the addition, stirring was continued for another 48 hours at the same temperature. DMF was removed under reduced pressure with a rotary evaporator. The yellowish residue was dissolved in 150 mL of DCM, washed with saturated aq. NH_4Cl (3 x 100 mL), dried over anhydrous Na_2SO_4 and filtered. The solvent was evaporated under reduced pressure by a rotary evaporator to leave a yellow solid that was purified on silica gel by flash column chromatography using DCM-methanol (99.5:0.5) to provide dimethylated macrocycle **1** (12.5 g, 82%) as a bright yellow solid, m.p. 153.8-155.1 °C; ^{1}H NMR (500 MHz, $CDCl_3$) δ 8.37 (dd, 2.0, 2.0 Hz, 2H), 8.26-8.24 (m, 4H), 8.07 (d, *J* = 8.0 Hz, 2H), 7.74 (s, 2H), 7.15 (d, *J* = 8.5 Hz, 2H), 4.34 (t, *J* = 5.0 Hz, 5.5 Hz, 4H), 3.84 (t, *J* = 5.5 Hz, 5.0 Hz, 4H), 3.75-3.69 (m, 12H), 2.42 (s, 6H); ^{13}C NMR (125 MHz, $CDCl_3$) 158.37, 156.40, 145.88, 136.53, 132.35, 130.14, 127.64, 127.27, 126.51, 125.41, 119.04, 112.95, 71.02, 70.66, 70.56, 69.52, 68.28, 16.69; HRMS (ESI) calcd for $C_{36}H_{39}N_2O_6$ $[M+H]^+$ m/z 595.2803, found m/z 595.2800; Anal. calcd. for $C_{36}H_{38}N_2O_6$: C, 72.71; H, 6.44; N, 4.71; found: C, 72.33; H, 6.51; N, 4.66.

Synthesis of mono-stoppered olefin 2

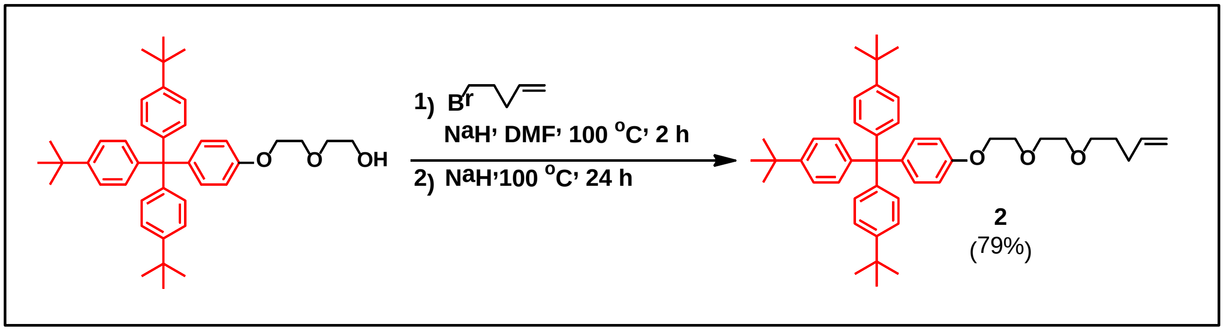

Scheme S2. Synthesis of compound **2**.

Sodium hydride (95%, 1.21 g, 50.6 mmol) was added to a solution of mono-stoppered linear alcohol (10.0 g, 16.9 mmol) and 5-bromo-1-pentene (4.02 g (V = 3.20 mL), 27.0 mmol) in 150 mL of dry DMF. This reaction mixture was stirred for 2 hours at 100 °C. A second portion of sodium hydride (95%, 1.21 g, 50.6 mmol) was added, and the reaction mixture was further stirred for 24 hours at 100 °C. The reaction mixture was concentrated under reduced pressure by a rotary evaporator. The yellowish white residue was dissolved in 50 mL DCM, washed with saturated aq. NH_4Cl (3 x 50 mL), dried over anhydrous Na_2SO_4 and filtered. The solvent was evaporated under reduced pressure by a rotary evaporator to leave a yellowish white solid which was purified on silica gel by flash column chromatography using hexanes-ethyl acetate (95:5) to provide the mono-stoppered olefin **2** (8.81 g, 79%) as a white solid, m.p. 151.4-152.3 °C; ^{1}H NMR (500 MHz, $CDCl_3$) δ 7.23 (d, J = 8.5 Hz, 6H), 7.07 (d, J = 8.5 Hz, 8H), 6.78 (d, J = 9.0 Hz, 2H), 5.85-5.76 (m, 1H), 5.03-4.93 (m, 2H), 4.11 (t, J = 4.5 Hz, 5.5 Hz, 2H), 3.85 (t, J = 5.0 Hz, 2H), 3.72-3.70 (m, 2H), 3.64-3.60 (m, 2H), 3.48 (t, J = 6.5 Hz, 7.0 Hz, 2H), 2.13-2.08 (m, 2H), 1.71-1.66 (m, 2H), 1.30 (s, 27H); ^{13}C NMR (125 MHz, $CDCl_3$) δ 156.56, 148.27, 144.12, 139.72, 138.27, 132.20, 130.71, 124.01, 114.69, 113.06, 70.82, 70.76, 70.15, 69.78, 67.22, 63.03, 34.28, 31.37, 30.22, 28.76; HRMS (ESI) calcd for $C_{46}H_{61}O_3$ $[M+H]^+$ m/z 661.4615, found m/z 661.4618; Anal. calcd. for $C_{46}H_{60}O_3$: C, 83.59; H, 9.15; found: C, 83.61; H, 8.85.

Synthesis of di-stoppered olefin 3

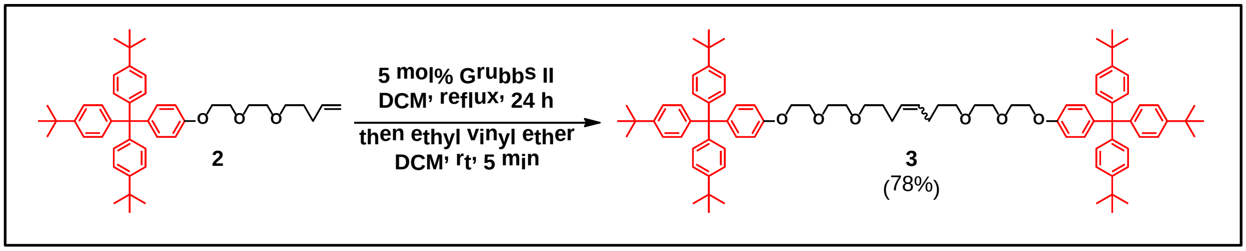

Scheme S3. Synthesis of compound **3**.

To a 45 mM DCM solution (5.3 mL) of mono-stoppered olefin **2** (160 mg, 0.242 mmol) under nitrogen was added Grubbs' second generation catalyst[12] (10.3 mg, 5 mol%). The reaction mixture was stirred at reflux for 24 hours. Excess ethyl vinyl ether (~0.5 mL) was added to quench the reaction. The solvent was evaporated under reduced pressure by a rotary evaporator to provide a brown solid that was purified on silica gel by flash column chromatography using DCM as eluent to provide di-stoppered olefin **3** (mixture of *cis*- and *trans*-) as a white solid (121.5 mg, 78%), m.p. 165.9-168.8 °C; ^{1}H NMR (500 MHz, $CDCl_3$) δ 7.23 (d, J = 8.5 Hz, 12H), 7.08 (d, J = 8.5 Hz, 16H), 6.77 (d, J = 9.0 Hz, 4H), 5.50-5.35 (m, 2H), 4.11 (t, J = 5.0 Hz, 4H), 3.85 (t, J = 5.0 Hz, 4H), 3.72-3.70 (m, 4H), 3.62-3.59 (m, 4H), 3.49-3.44 (m, 4H), 2.11-2.02 (m, 4H), 1.67-1.61 (m, 4H), 1.30 (s, 54H); ^{13}C NMR (125 MHz, $CDCl_3$) δ 156.56, 148.27, 144.12, 139.70, 132.19, 130.71(*trans*), 130.01(*cis*), 124.01, 114.69, 113.05, 70.85, 70.80, 70.13, 69.79, 67.20, 63.03, 34.27, 31.37, 29.41, 28.99; HRMS (ESI) calcd for $C_{90}H_{116}NaO_6$ [M+Na]$^+$ *m/z* 1315.8664, found *m/z* 1315.8628; Anal. calcd. for $C_{90}H_{116}O_6$: C, 83.54; H, 9.04; found: C, 83.32; H, 9.09.

Synthesis of metalated [2]pseudorotaxane 4

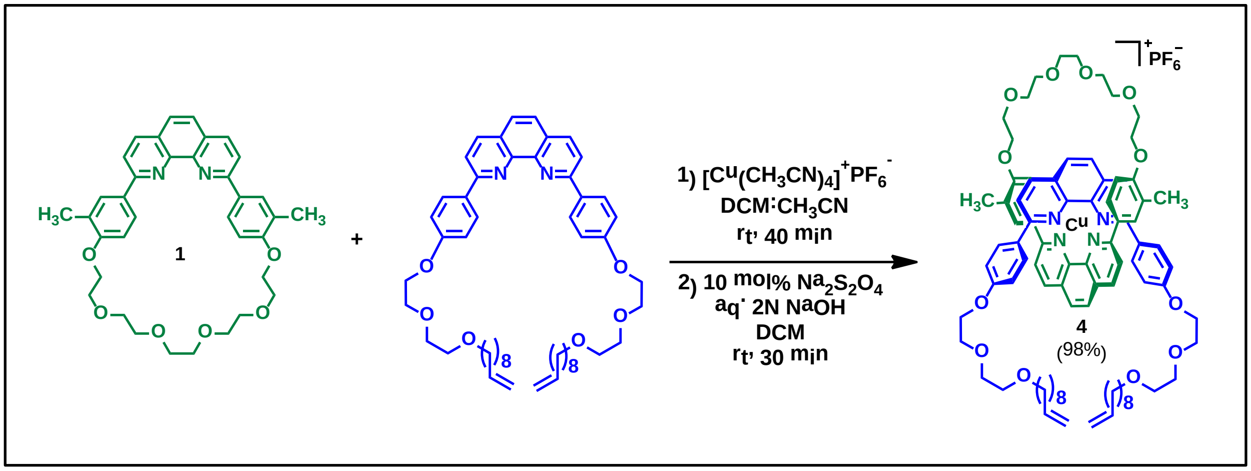

Scheme S4. Synthesis of compound **4**.

To a solution of dimethylated macrocycle **1** (240 mg, 0.404 mmol) in dichloromethane (20 mL) and acetonitrile (20 mL) at room temperature under nitrogen was added tetrakis(acetonitrile)copper(I) hexafluorophosphate (150 mg, 0.404 mmol) and stirred for 20 min. A DCM solution (20 mL) of the diolefin precursor[3] (330 mg, 0.404 mmol) was added from another Schlenk flask under nitrogen via cannula. The reaction mixture was stirred for 40 min at room temperature under nitrogen followed by concentration of the mixture under reduced pressure by a rotary evaporator. Purification on silica gel by flash column chromatography using DCM-methanol (98:2) as eluent afforded the partially oxidized metalated [2]pseudorotaxane **4**. The partially oxidized product was dissolved in 10 mL DCM, and 5 mg sodium dithionite (90%), and five drops of 2N aqueous sodium hydroxide were added to the solution and the mixture was stirred for 30 min at room temperature. After 30 min, the solution was filtered through a fritted-funnel which was filled with 1 cm height of Celite 545 and 1 cm height of anhydrous Na_2SO_4 to provide reduced and pure metalated [2]pseudorotaxane **4** as a red glassy solid (647 mg, 98%). ^1H NMR (500 MHz, $CDCl_3$) δ 8.63 (d, *J* = 8.5 Hz, 2H), 8.47 (d, *J* = 8.5 Hz, 2H), 8.20 (s, 2H), 8.02 (s, 2H), 7.86 (d, *J* = 8.5 Hz, 2H), 7.78 (d, *J* = 8.5 Hz, 2H), 7.47 (d, *J* = 8.0 Hz, 4H), 7.15 (d, J = 8.0 Hz, 2H), 6.95 (s, 2H), 6.08 (d, *J* = 8.5 Hz, 4H), 5.81 (d, *J* = 8.5 Hz, 2H), 5.79-5.72 (m, 2H), 4.96-4.87 (m, 4H), 3.86 (s, 4H), 3.78 (s, 8H), 3.74-3.70 (m, 8H), 3.65-3.62 (m, 12H), 3.59-3.57 (m, 4H), 3.48 (t, *J* = 7.0 Hz, 4H), 2.01-1.97 (m, 4H), 1.62-1.57 (m, 4H), 1.48 (s, 6H), 1.38-1.21 (m, 20H); ^{13}C NMR (125 MHz, $CDCl_3$) δ 159.26, 156.98, 156.70, 155.46, 143.20, 143.18,

139.05, 137.58, 136.76, 132.00, 131.06, 130.04, 128.96, 127.91, 127.65, 126.94, 126.30, 126.04, 125.62, 123.90, 123.84, 114.02, 112.90, 109.18, 71.46, 71.02, 70.77, 70.77, 70.69, 69.99, 69.33, 69.25, 67.35, 67.26, 33.65, 29.56, 29.31, 29.31, 28.94, 28.77, 25.98, 15.59; HRMS (ESI) calcd for $C_{88}H_{106}CuN_4O_{12}$ $[M-PF_6]^+$ m/z 1473.7098, found m/z 1473.7084; Anal. calcd. for $C_{88}H_{106}CuF_6N_4O_{12}P$: C, 65.23; H, 6.59; N, 3.46; found: C, 64.86; H, 6.36; N, 3.38.

Synthesis of metalated [2]catenane 5

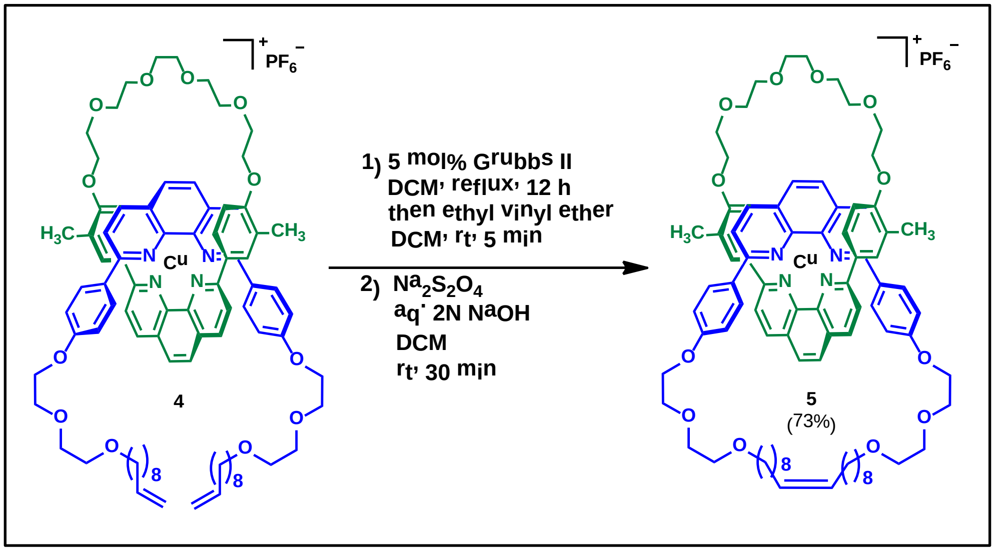

Scheme S5. Synthesis of compound **5**.

Grubbs' second generation catalyst[12] (13.1 mg, 5 mol%) was added to a 3.20 mM solution of metalated [2]pseudorotaxane **4** (500 mg, 0.309 mmol) in DCM (96 mL) under nitrogen at reflux. After the reaction mixture was stirred for 6 h at 40 °C, additional catalyst (13.1 mg, 5 mol%) was added and stirring was continued for another 6 h. Excess ethyl vinyl ether (1 mL) was then added into the mixture to quench the reaction. The solvent was evaporated under reduced pressure by a rotary evaporator to leave a dark red glassy solid that was purified on silica gel by flash column chromatography using DCM-methanol (99:1) as eluent afforded the partially oxidized metalated [2]catenane **5**. The partially oxidized product was dissolved in 10 mL DCM, and 5 mg sodium dithionite (90%), and five drops of 2N aqueous sodium hydroxide were added to the solution and the mixture was stirred for 30 min at room temperature. After 30 min, the solution was filtered through a fritted-funnel which was filled with 1 cm height of Celite 545 and 1 cm height of

anhydrous Na_2SO_4 to provide reduced and pure metalated [2]catenane **5** as a red glassy solid (358.7 mg, 73%). ^1H NMR (500 MHz, $CDCl_3$) δ 8.64 (d, J = 8.5 Hz, 2H), 8.48 (d, J = 8.0 Hz, 2H), 8.22 (s, 2H), 8.05 (s, 2H), 7.88 (d, J = 8.0 Hz, 2H), 7.80 (d, J = 8.0 Hz, 2H), 7.48 (d, J = 8.0 Hz, 4H), 7.18 (d, J = 8.0 Hz, 2H), 6.97 (s, 2H), 6.09 (d, J = 8.5 Hz, 4H), 5.83 (d, J = 8.5 Hz, 2H), 5.37-5.30 (m, 2H) (*trans*- and *cis*-), 3.88 (s, 4H), 3.78 (s, 8H), 3.75-3.73 (m, 8H), 3.71-3.69 (m, 4H), 3.66-3.65 (m, 8H), 3.61-3.59 (m, 4H), 3.54 (t, 4H), 1.98-1.90 (m, 4H), 1.67-1.62 (m, 4H), 1.50 (s, 6H), 1.38-1.25 (m, 20H); ^{13}C NMR (125 MHz, $CDCl_3$) δ 159.47, 157.24, 156.96, 155.77, 143.49, 143.47, 137.85, 137.04, 132.26, 131.39, 130.57 (*trans*), 130.33, 130.06 (*cis*), 129.25, 128.18, 127.95, 127.20, 126.61, 126.41, 125.87, 124.18, 124.12, 113.12, 109.43, 71.77, 71.31, 71.08, 71.05, 71.05, 70.43, 69.65, 69.54, 67.57, 67.57, 32.46, 29.80, 29.41, 29.40, 29.38, 28.71, 26.19, 15.88; HRMS (ESI) calcd for $C_{86}H_{102}CuN_4O_{12}$ $[M-PF_6]^+$ m/z 1445.6785, found m/z 1445.6782; Anal. calcd for $C_{86}H_{102}CuF_6N_4O_{12}P$: C, 64.87; H, 6.46; N, 3.52; found: C, 64.63; H, 6.17; N, 3.38.

Synthesis of metalated [2]rotaxane 6

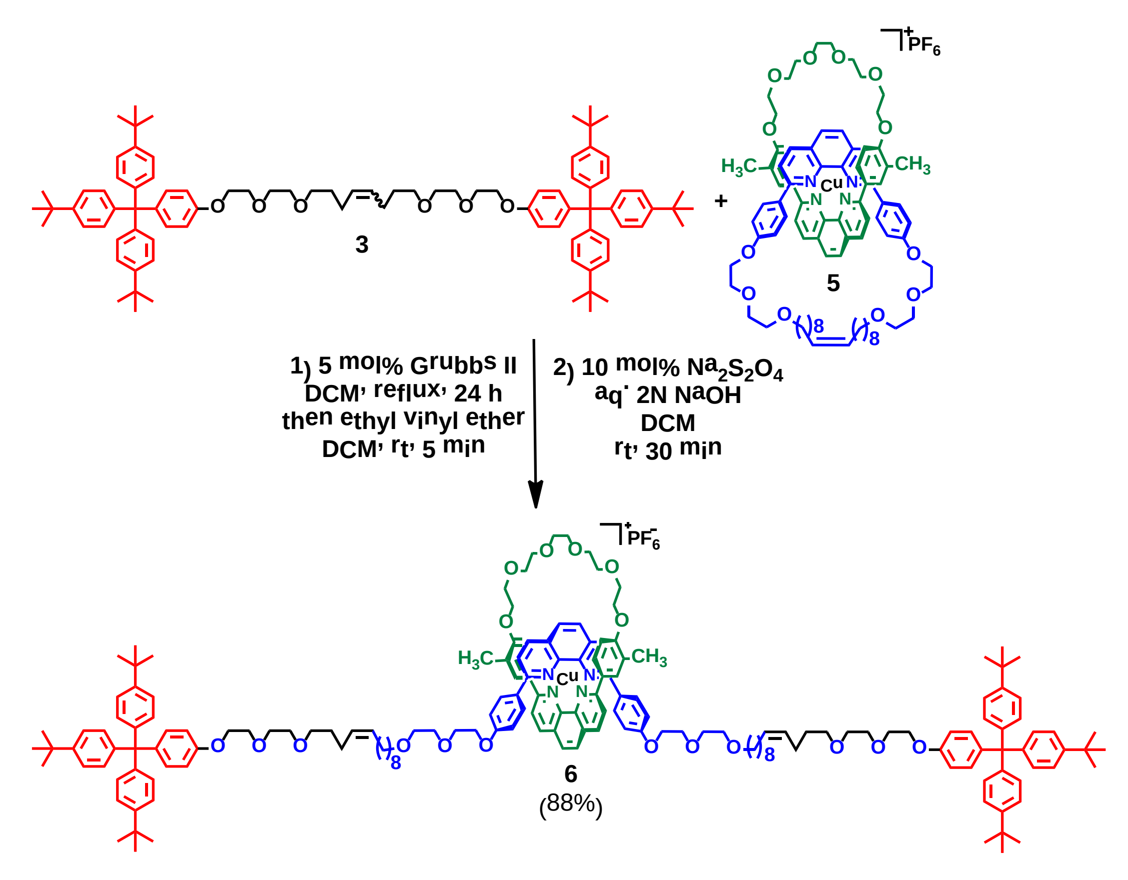

Scheme S6. Synthesis of compound **6**.

To a 10 mM solution of metalated [2]catenane **5** (30.0 mg, 0.0188 mmol) in DCM (1.88 mL) under nitrogen was added the di-stoppered olefin **3** (243.8 mg, 0.188 mmol). A DCM solution (1.0 mL) of Grubbs' second generation catalyst[12] (0.800 mg, 5 mol%) was added via syringe and the reaction mixture was stirred at reflux for 24 hours. An excess of ethyl vinyl ether (~0.3 mL) was added to quench the reaction. The solvent was removed under reduced pressure to afford a dark brown residue. Flash column chromatography on silica gel with DCM-methanol (95:5) as eluent afforded the partially oxidized metalated [2]rotaxane **6**. The partially oxidized product was dissolved in 10 mL DCM, and 5 mg sodium dithionite (90%), and five drops of 2N aqueous sodium hydroxide were added to the solution and the mixture was stirred for 30 min at room temperature. After 30 min, the solution was filtered through a fritted-funnel which was filled with 1 cm height of Celite 545 and 1 cm height of anhydrous Na_2SO_4 to provide reduced and pure metalated [2]rotaxane **6** as a dark brown solid (48.1 mg, 88%). ^{1}H NMR (400 MHz, $CDCl_3$) δ 8.64 (d, *J* = 8.2 Hz, 2H), 8.47 (d, *J* = 8.2 Hz, 2H), 8.22 (s, 2H), 8.02 (s, 2H), 7.88 (d, *J* = 8.2 Hz, 2H), 7.80 (d, *J* = 8.2 Hz, 2H), 7.48 (d, *J* = 8.7 Hz, 4H), 7.23-7.15 (m, 14H), 7.07 (d, *J* = 8.7 Hz, 16H), 6.96 (s, 2H), 6.77 (d, *J* = 8.7 Hz, 4H), 6.10 (d, *J* = 8.7 Hz, 4H), 5.82 (d, *J* = 8.2 Hz, 2H), 5.46-5.31 (m, 4H), 4.10 (t, *J* = 4.6 Hz, 4H), 3.88-3.43 (m, 56H), 2.04-1.74 (m, 8H), 1.69-1.59 (m, 8H), 1.50 (s, 6H), 1.38-1.26 (m, 74H); ^{13}C NMR (100 MHz, $CDCl_3$) δ 159.49, 157.18, 156.96, 156.68, 155.74, 148.41, 144.26, 143.46, 139.85, 137.77, 137.01, 132.34, 132.34, 132.27, 131.30, 131.05, 131.00, 130.90, 130.84, 130.84 (*trans*), 130.30 (*cis*), 129.55, 129.20, 128.14, 127.88, 127.15, 126.58, 126.28, 125.82, 124.16, 124.16, 113.18, 109.30, 71.78, 71.29, 71.04, 71.04, 70.94, 70.94, 70.22, 70.21, 69.91, 69.57, 69.56, 67.51, 67.50, 67.32, 63.16, 34.43, 32.72, 31.52, 31.52, 29.81, 29.80, 29.73, 29.62, 29.61, 29.29, 29.15, 26.25, 15.89; HRMS (MALDI) calcd for $C_{176}H_{219}CuN_4O_{18}$ $[M+H-PF_6]^{++}$ m/z 2739.5630, found m/z 2739.5598; Anal. calcd for $C_{176}H_{218}CuF_6N_4O_{18}P$: C, 73.24; H, 7.61; N, 1.94; found: C, 73.57; H, 7.24; N, 1.99.

Synthesis of core complex 7

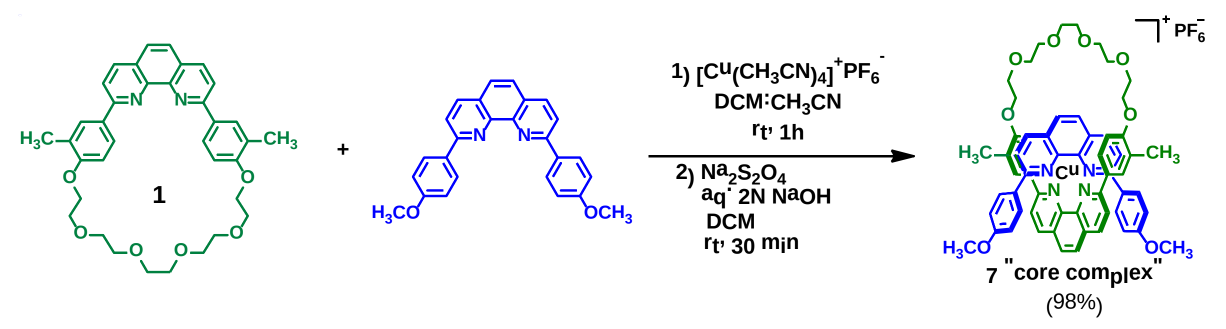

Scheme S7. Synthesis of compound **7**.

To a solution of dimethylated macrocycle **1** (205 mg, 0.362 mmol) in dichloromethane (10 mL) and acetonitrile (10 mL) at room temperature under nitrogen was added tetrakis(acetonitrile)copper(I) hexafluorophosphate (123 mg, 0.463 mmol) and stirred for 20 min. A dicholoromethane (5 mL) and acetonitrile (5 mL) solution of 2,9-di(4-methoxyphenyl)-1,10-phenanthroline[1] (142 mg, 0.362 mmol) was added from another Schlenk flask under nitrogen via cannula. The reaction mixture was stirred for two hours at room temperature under nitrogen followed by concentration of the mixture under reduced pressure by a rotary evaporator. Purification on SEC column chromatography using DCM as eluent afforded the partially oxidized core complex **7**. The partially oxidized product was dissolved in 10 mL DCM, and 5 mg sodium dithionite (90%), and five drops of 2N aqueous sodium hydroxide were added to the solution and the mixture was stirred for 30 min at room temperature. After 30 min, the solution was filtered through a fritted-funnel which was filled with 1 cm height of Celite 545 and 1 cm height of anhydrous Na_2SO_4 to provide reduced and pure core complex **7** (400 mg, 98%) as a red glassy solid, m.p. 252.4-253.0 °C; ^{1}H NMR (500 MHz, $CDCl_3$) δ 8.64 (d, J = 8.5 Hz, 2H), 8.46 (d, J = 8.0 Hz, 2H), 8.22 (s, 2H), 8.00 (s, 2H), 7.89 (d, J = 8.0 Hz, 2H), 7.80 (d, J = 8.0 Hz, 2H), 7.51 (d, J = 8.5 Hz, 4H), 7.18 (d, J = 8.5 Hz, 2H), 6.95 (s, 2H), 6.08 (d, J = 8.5 Hz, 4H), 5.81 (d, J = 8.0 Hz, 2H), 3.88 (s, 4H), 3.76-3.74 (m, 4H), 3.67-3.64 (m, 8H), 3.61-3.59 (m, 4H), 3.52 (s, 6H), 1.51 (s, 6H); ^{13}C NMR (125 MHz, $CDCl_3$) δ 160.28, 157.24, 157.06, 155.74, 143.46, 137.81, 136.86, 132.22, 131.20, 130.30, 129.28, 129.28, 128.16, 127.84, 127.20, 126.56, 126.09, 125.85, 124.19, 112.58, 112.58, 109.38, 71.31, 71.06, 71.06, 69.52, 67.52, 55.38, 15.88 ; HRMS (ESI) calcd for

$C_{62}H_{58}CuN_4O_8$ [M-PF6]+ m/z 1049.3545, found m/z 1049.3521; Anal. calcd. for $C_{62}H_{58}CuF_6N_4O_8P$: C, 62.28; H, 4.89; N, 4.69; found: C, 62.35; H, 4.69; N, 4.62.

Synthesis of demetalated [2]catenane 8

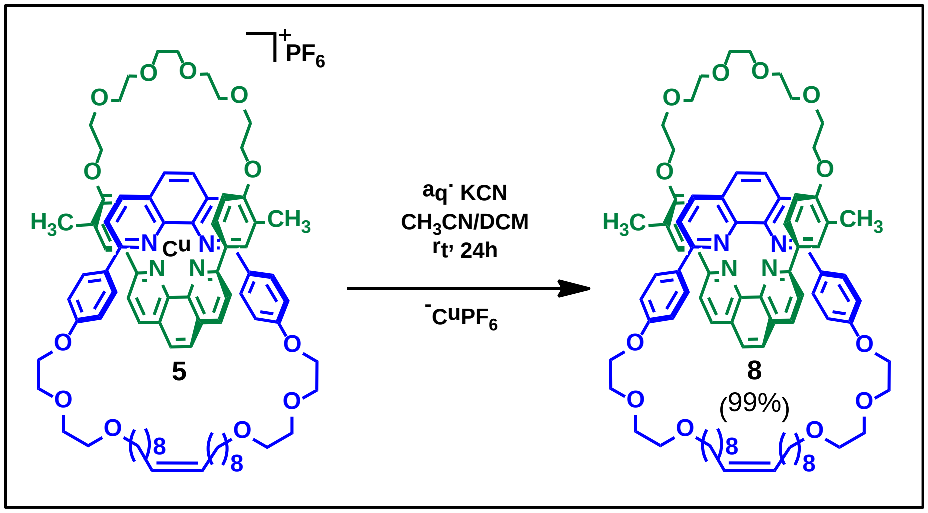

Scheme S8. Alternative synthesis of compound **8**.

The metalated [2]catenane **5** (112 mg, 0.0703 mmol) was dissolved in a solvent mixture containing dichloromethane (10 mL) and acetonitrile (10 mL) to give a dark brown solution, into which a solution of KCN (137 mg, 2.11 mmol) in H_2O (2 mL). The reaction mixture was stirred for 2 hours at room temperature, accompanied by a gradual disappearance of the brown color of the biphase mixture. After completion of a color change from dark red to near colorless, the volatile solvents were removed under reduced pressure. The aqueous residue was washed with dichloromethane (2 x 20 mL); the combined dichloromethane extracts were washed with saturated aqueous sodium chloride (50 mL), and dried over anhydrous Na_2SO_4 and filtered. The solvent was removed under reduced pressure to provide a crude off-white product that was purified by silicagel flash column chromatography eluted with methanol/dichloromethane (0.5:99.5 – 1:99) to give pure demetalated [2]catenane **8** which was further analyzed by analytical SEC; ^{1}H NMR (400 MHz, $CDCl_3$) δ 8.97 (dd, *J* = 2.3 Hz, 2H), 8.42 (d, *J* = 8.7 Hz, 4H), 8.24 (d, *J* = 8.3 Hz, 2H), 8.19 (d, *J* = 8.7 Hz, 2H), 8.08 (s, 2H), 8.06 (s, 2H), 7.77 (s, 2H), 7.71 (d, *J* = 10.1 Hz, 4H), 7.25-7.17 (m, 6H), 5.41-5.34 (m,

2H) (*trans*- and *cis*-), 4.28-4.26 (m, 4H), 4.20-4.17 (m, 4H), 3.97-3.90 (m, 4H), 3.77-3.67 (m, 20H), 3.59-3.53 (m, 4H), 3.39-3.34 (m, 4H), 2.27 (s, 6H), 2.01-1.96 (m, 4H), 1.51-1.48 (m, 4H), 1.31-1.23 (m, 20H); ^{13}C NMR (100 MHz, $CDCl_3$) δ 160.27, 158.21, 156.47, 156.47, 146.30, 146.10, 136.70, 136.50, 132.10, 132.03, 130.53 (*trans*), 130.05 (*cis*), 129.10, 129.10, 128.77, 128.28, 127.53, 127.37, 126.02, 125.60, 125.46, 119.44, 119.06, 115.25, 112.95, 71.58, 71.12, 71.00, 70.89, 70.63, 70.30, 70.06, 68.50, 67.72, 66.22, 32.61, 29.71, 29.55, 29.44, 29.32, 28.97, 26.18, 16.63; HRMS (ESI) calcd for $C_{86}H_{102}N_4O_{12}$ $[M+H]^+$ m/z 1382.7489, found m/z 1382.7393.

Synthesis of demetalated [2]rotaxane 9

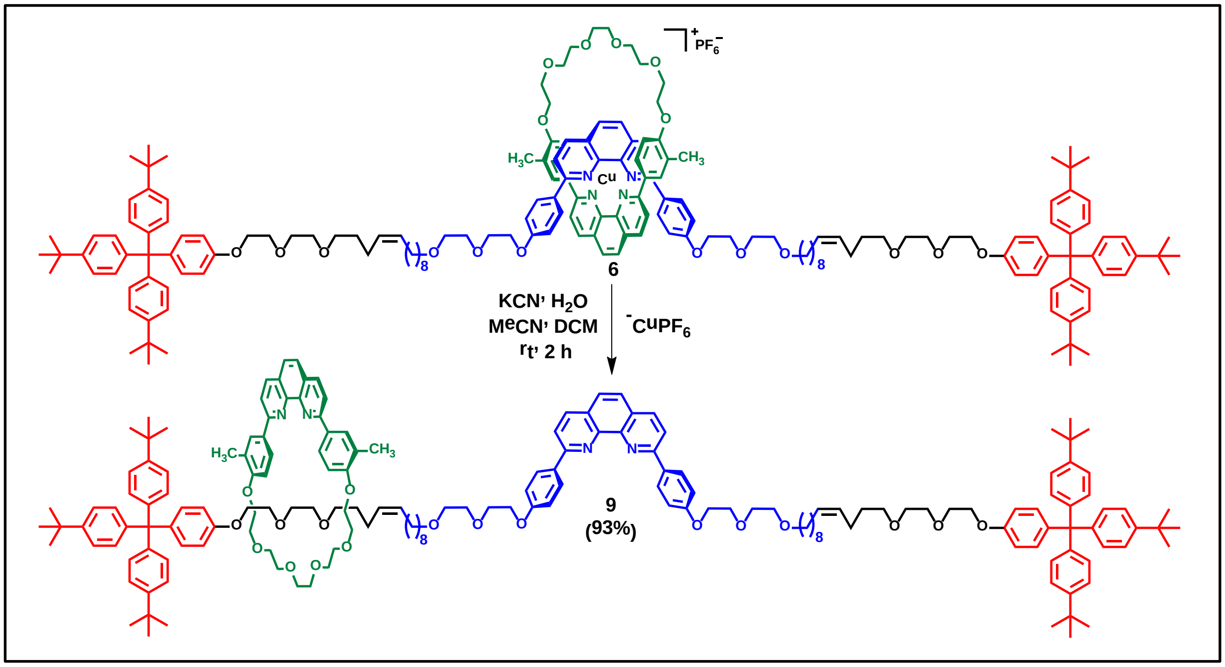

Scheme S9. Alternative synthesis of compound **9**.

The metalated [2]rotaxane **6** (15.0 mg, 5.20 μmol)) was dissolved in dichloromethane (5 mL) and acetonitrile (5 mL). To this solution was added an aqueous solution of excess KCN (10.2 mg, 0.156 mmol in 1 mL of water). The reaction mixture was stirred for 2 hours at room temperature. After completion of a color change from dark red to near colorless, the volatile solvents were removed under reduced pressure. The aqueous residue was washed with dichloromethane (2 x 10 mL); the combined dichloromethane extracts were washed with saturated aqueous sodium chloride (20 mL), and dried over anhydrous Na_2SO_4 and filtered.

The solvent was removed under reduced pressure to provide a crude off-white product that was purified by silicagel flash column chromatography eluted with methanol/dichloromethane (0.5:99.5 – 1:99) to give pure demetalated [2]rotaxane **9** which was further analyzed by analytical SEC; ^{1}H NMR (400 MHz, $CDCl_3$) δ 8.92 (d, *J* = 8.7 Hz, 2H), 8.40 (d, *J* = 8.2 Hz, 4H), 8.21 (d, *J* = 8.2 Hz, 4H), 8.07-8.03 (m, 4H), 7.75 (s, 2H), 7.70 (s, 4H), 7.23-7.15 (m, 18H), 7.06 (d, *J* = 8.7 Hz, 16H), 6.83-6.75 (m, 4H), 5.38-5.25 (m, 4H), 4.27-3.35 (m, 60H), 2.27 (s, 6H), 2.04-1.89 (m, 8H), 1.68-1.44 (m, 8H), 1.37-1.25 (m, 74H); ^{13}C NMR (100 MHz, $CDCl_3$) δ 160.27, 158.22, 156.73, 156.50, 148.35, 148.35, 146.15, 144.31, 139.74, 136.82, 136.50, 132.33, 130.85, 130.85 (*trans*), 129.56, 129.11 (*cis*), 128.78, 128.77, 128.75, 128.24, 128.21, 128.19, 127.62, 127.40, 125.67, 125.49, 124.15, 124.15, 119.49, 115.16, 113.38, 113.16, 112.96, 71.72, 71.02, 71.02, 70.90, 70.82, 70.58, 70.24, 70.24, 69.98, 69.96, 68.48, 67.69, 67.39, 66.27, 63.18, 34.42, 34.41, 32.75, 32.07, 31.51, 29.85, 29.80, 29.63, 29.76, 26.24, 22.85, 16.67, 14.28; HRMS (MALDI) calcd for $C_{176}H_{219}N_4O_{18}$ $[M+H]^+$ m/z 2376.6339, found m/z 2376.3344.

Section C. NMR Spectroscopy

^{1}H NMR Spectrum of Macrocycle 1

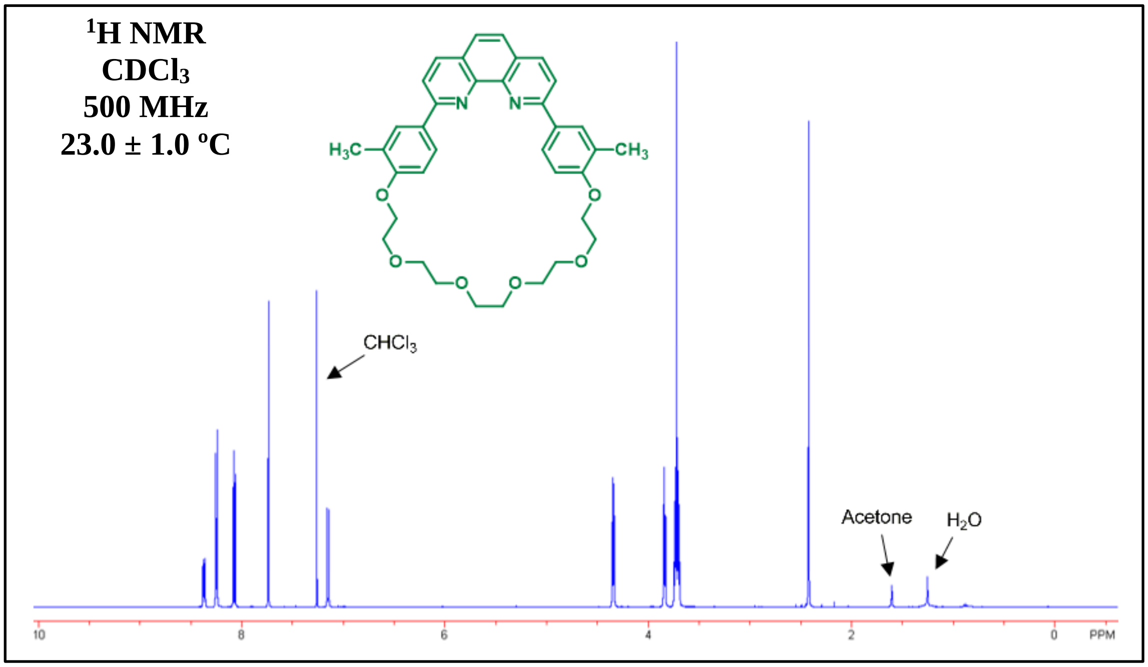

Figure S1. Annotated ^{1}H NMR spectrum (500 MHz, CDCl$_3$, 23 ºC) of **1**.

^{13}C NMR Spectrum of Macrocycle 1

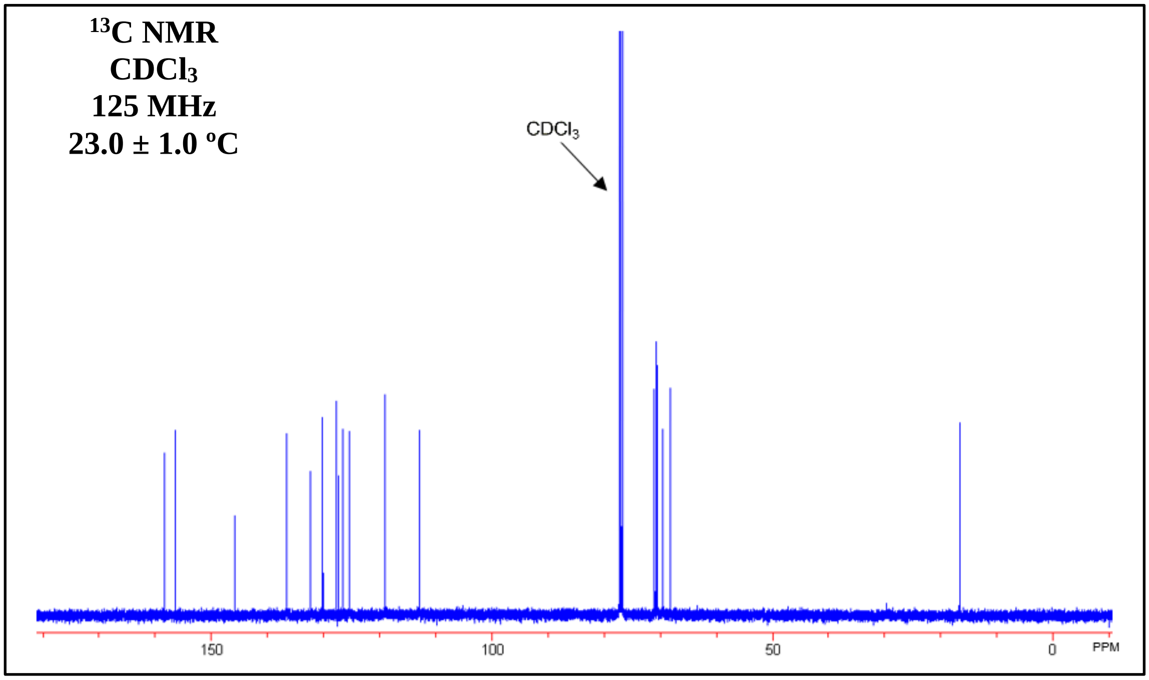

Figure S2. Annotated ^{13}C NMR spectrum (125 MHz, CDCl$_3$, 23 ºC) of **1**.

^{1}H NMR Spectrum of Mono-stoppered Olefin 2

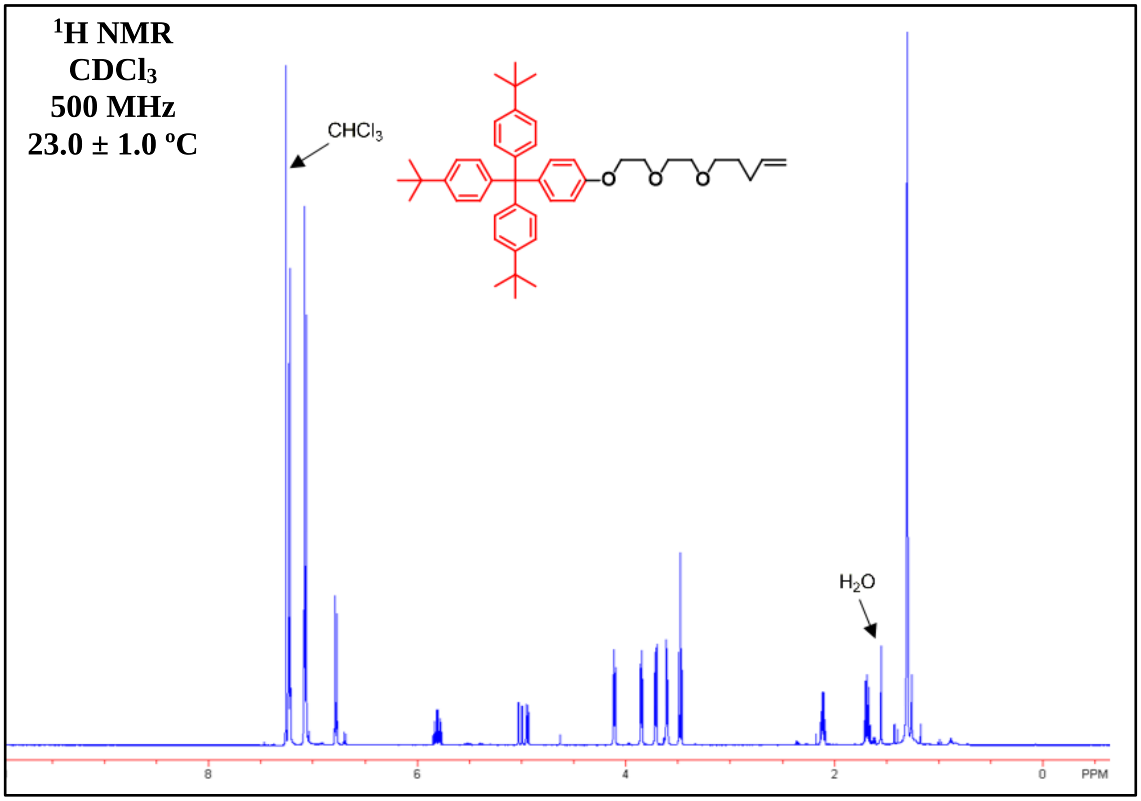

Figure S3. Annotated ^{1}H NMR spectrum (500 MHz, $CDCl_3$, 23 ºC) of **2**.

^{13}C NMR Spectrum of Mono-stoppered Olefin 2

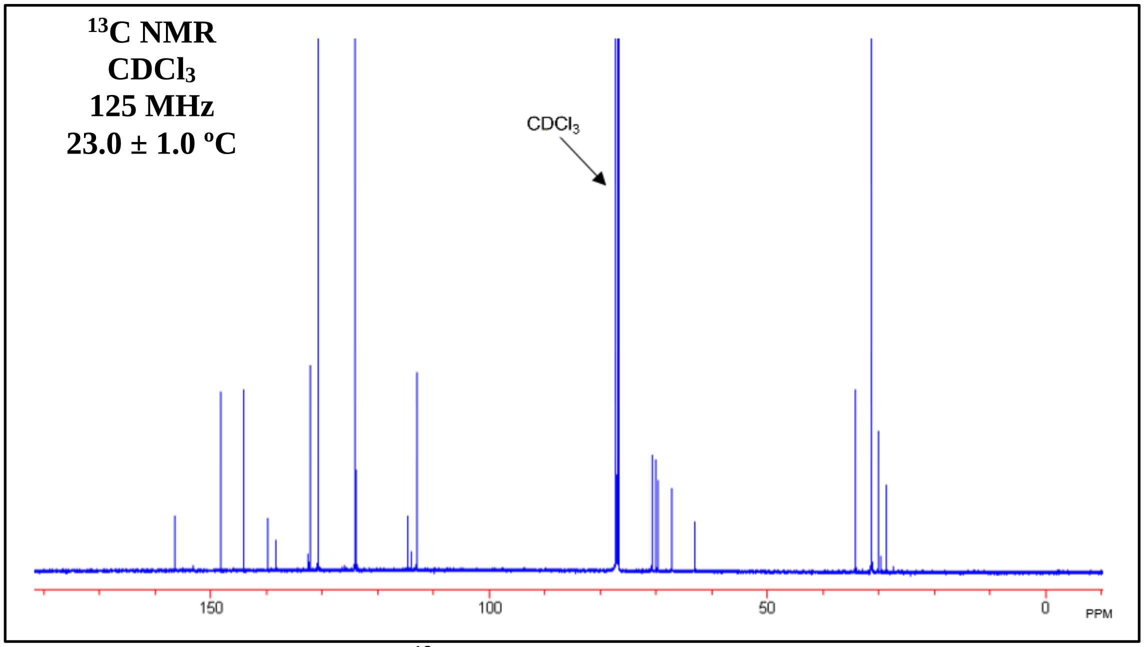

Figure S4. Annotated ^{13}C NMR spectrum (125 MHz, $CDCl_3$, 23 ºC) of **2**.

^{1}H NMR Spectrum of Di-stoppered Olefin 3

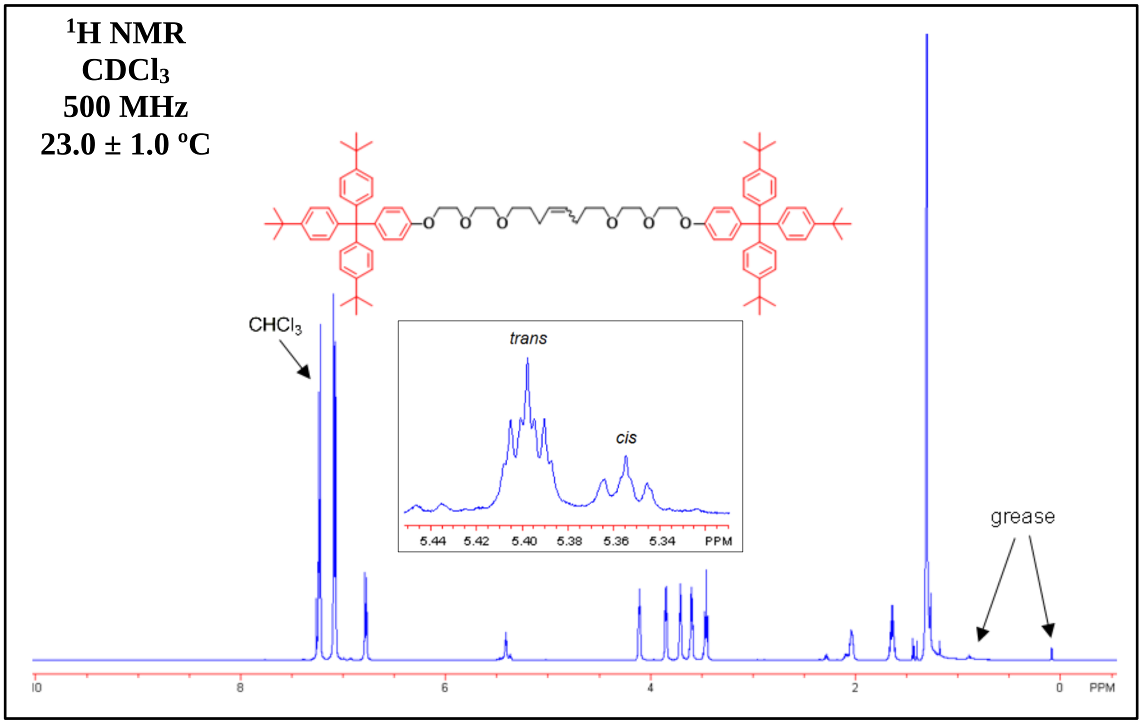

Figure S5. Annotated ^{1}H NMR spectrum (500 MHz, $CDCl_3$, 23 ºC) of **3**.

^{13}C NMR Spectrum of Di-stoppered Olefin 3

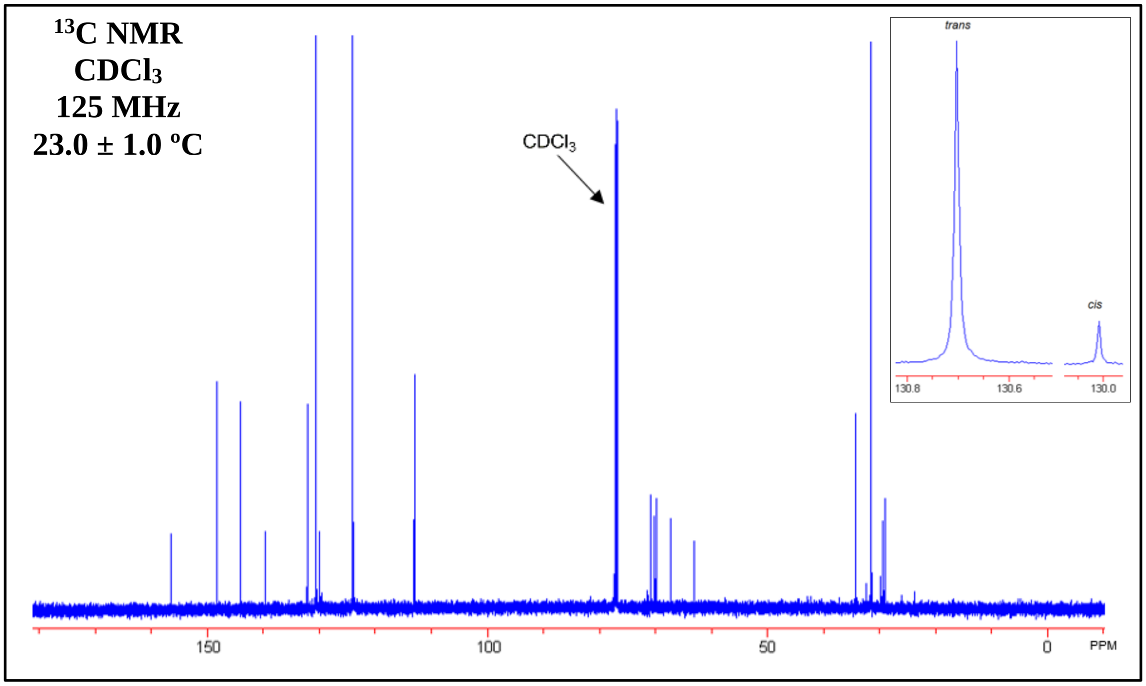

Figure S6. Annotated ^{13}C NMR spectrum (125 MHz, $CDCl_3$, 23 ºC) of **3**.

^{1}H NMR Spectrum of Metalated [2]Pseudorotaxane 4

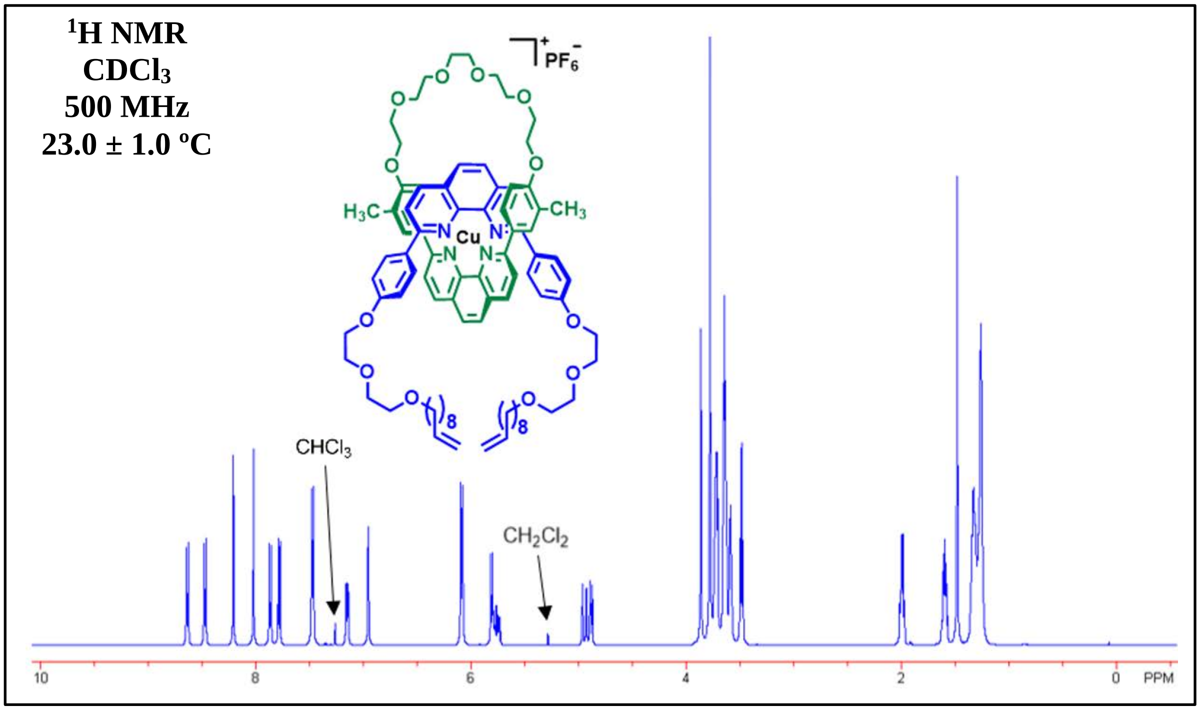

Figure S7. Annotated ^{1}H NMR spectrum (500 MHz, $CDCl_3$, 23 ºC) of **4**.

^{13}C NMR Spectrum of Metalated [2]Pseudorotaxane 4

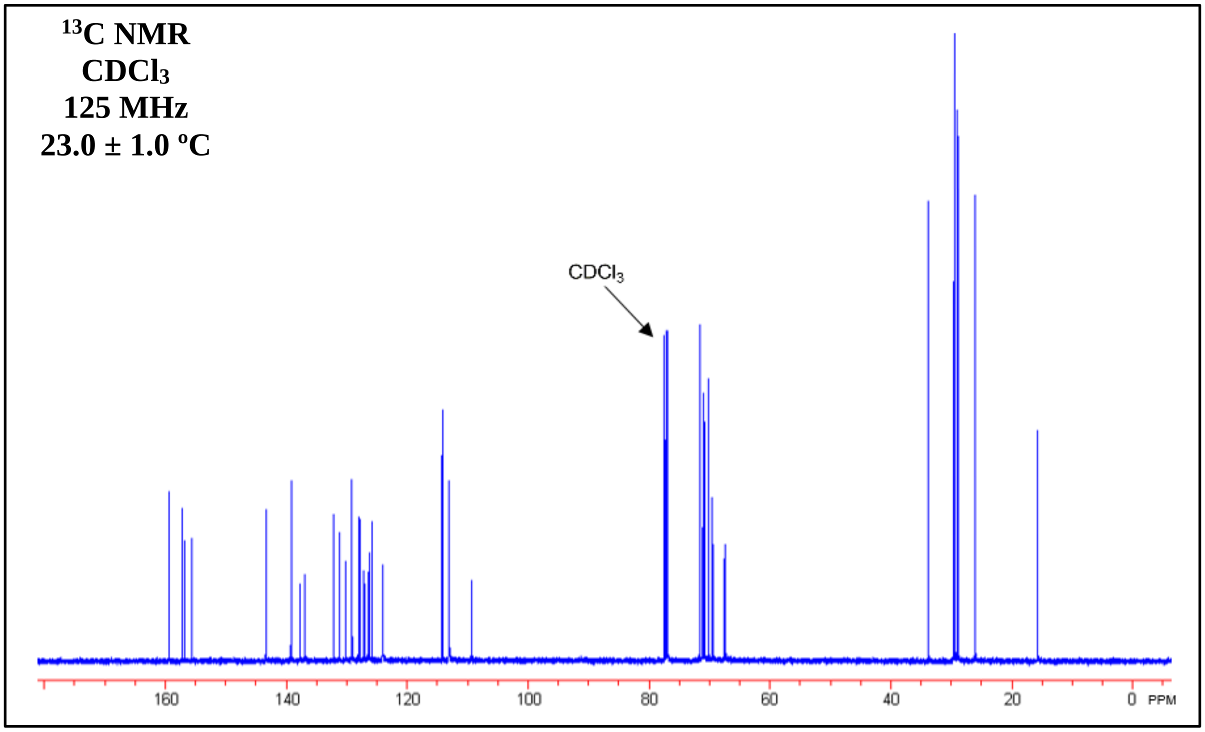

Figure S8. Annotated ^{13}C NMR spectrum (125 MHz, $CDCl_3$, 23 ºC) of **4**.

COSY NMR Spectrum of Metalated [2]Pseudorotaxane 4

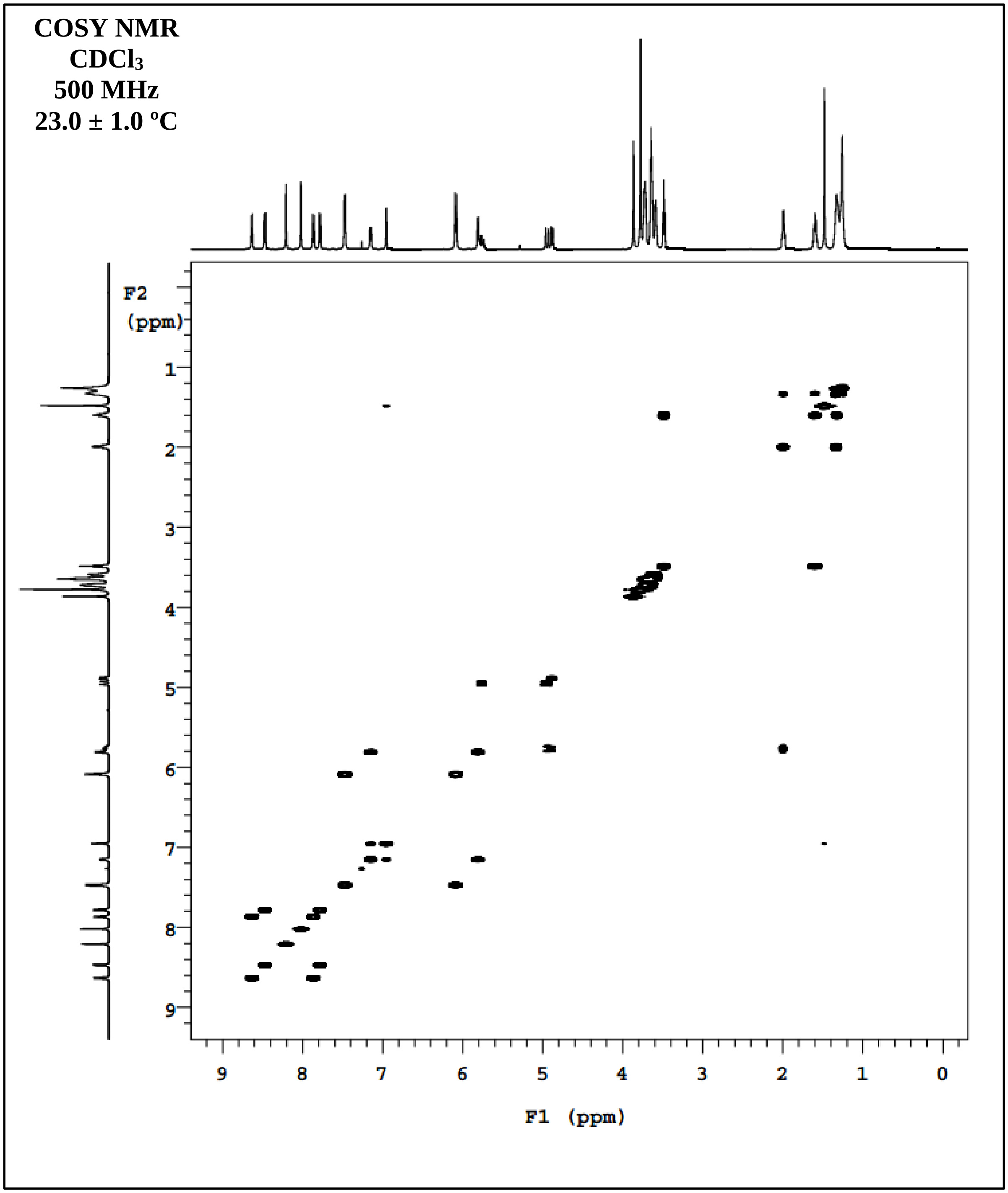

Figure S9. COSY NMR spectrum (500 MHz, $CDCl_3$, 23 ºC) of **4**.

COSY NMR Spectrum of Metalated [2]Pseudorotaxane 4 – Expansion #1

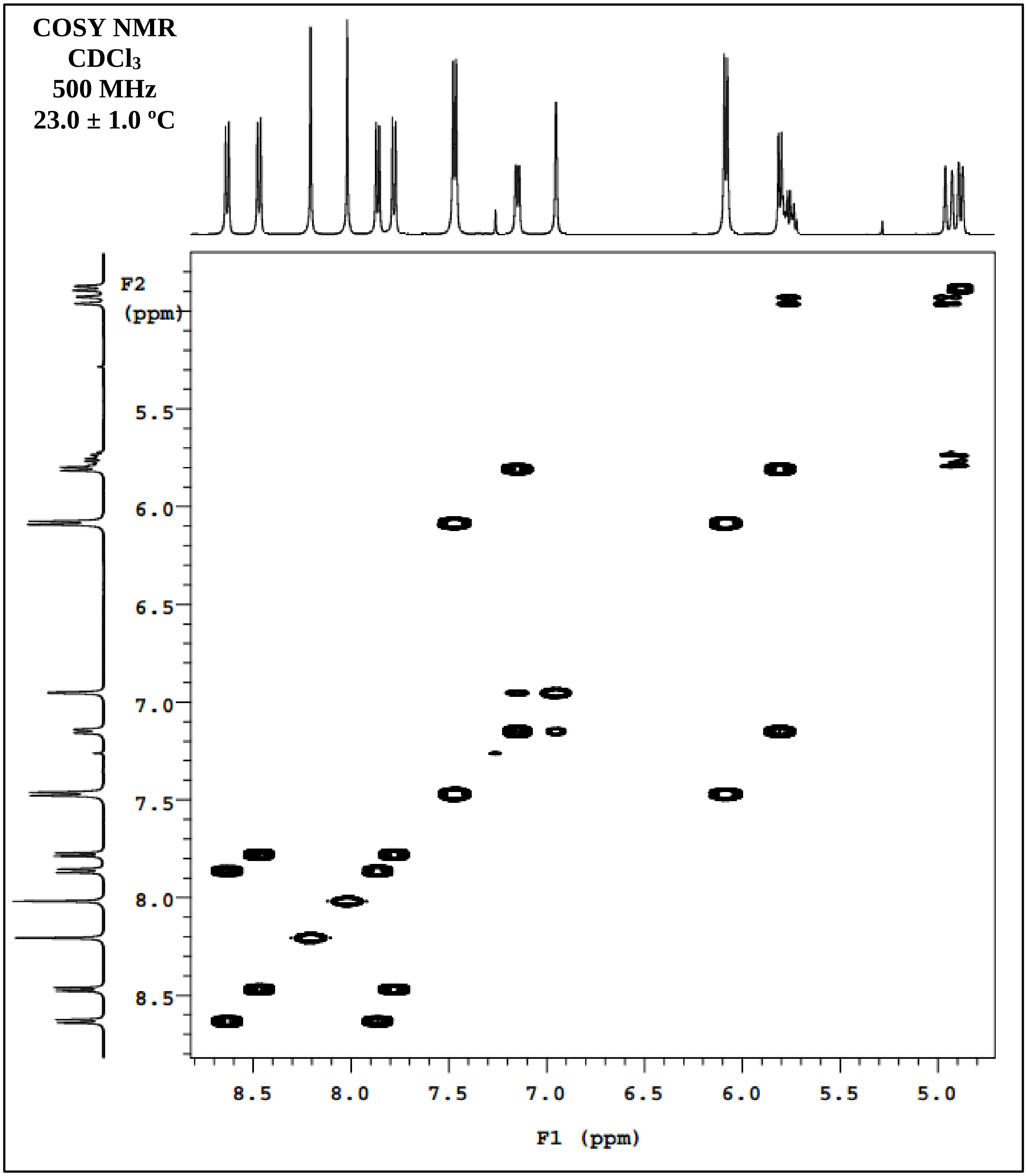

Figure S10. Expansion #1 of COSY NMR spectrum (500 MHz, $CDCl_3$, 23 ºC) of **4**.

COSY NMR Spectrum of Metalated [2]Pseudorotaxane 4 – Expansion #2

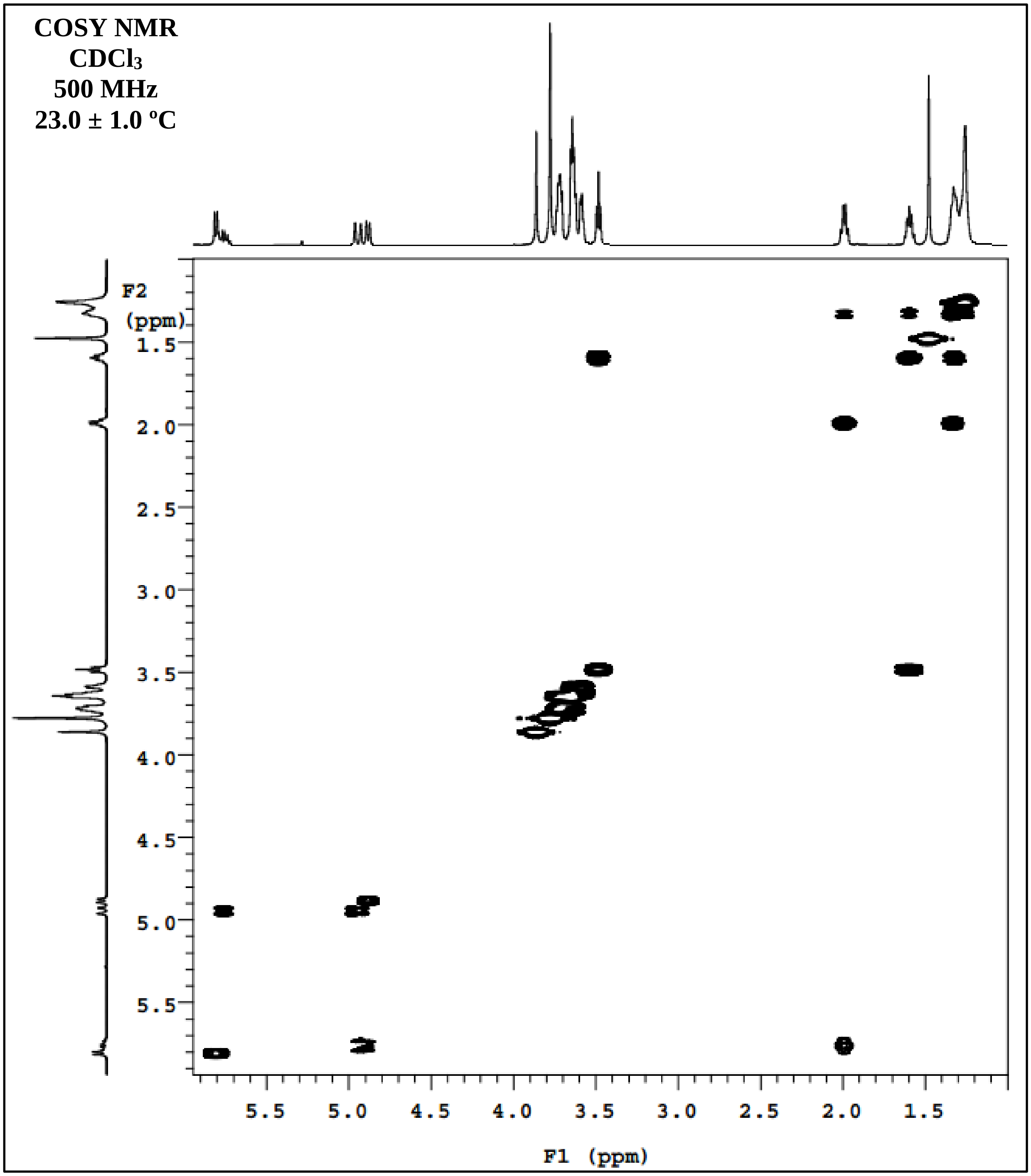

Figure S11. Expansion #2 of COSY NMR spectrum (500 MHz, $CDCl_3$, 23 ºC) of **4**.

NOESY NMR Spectrum of Metalated [2]Pseudorotaxane 4

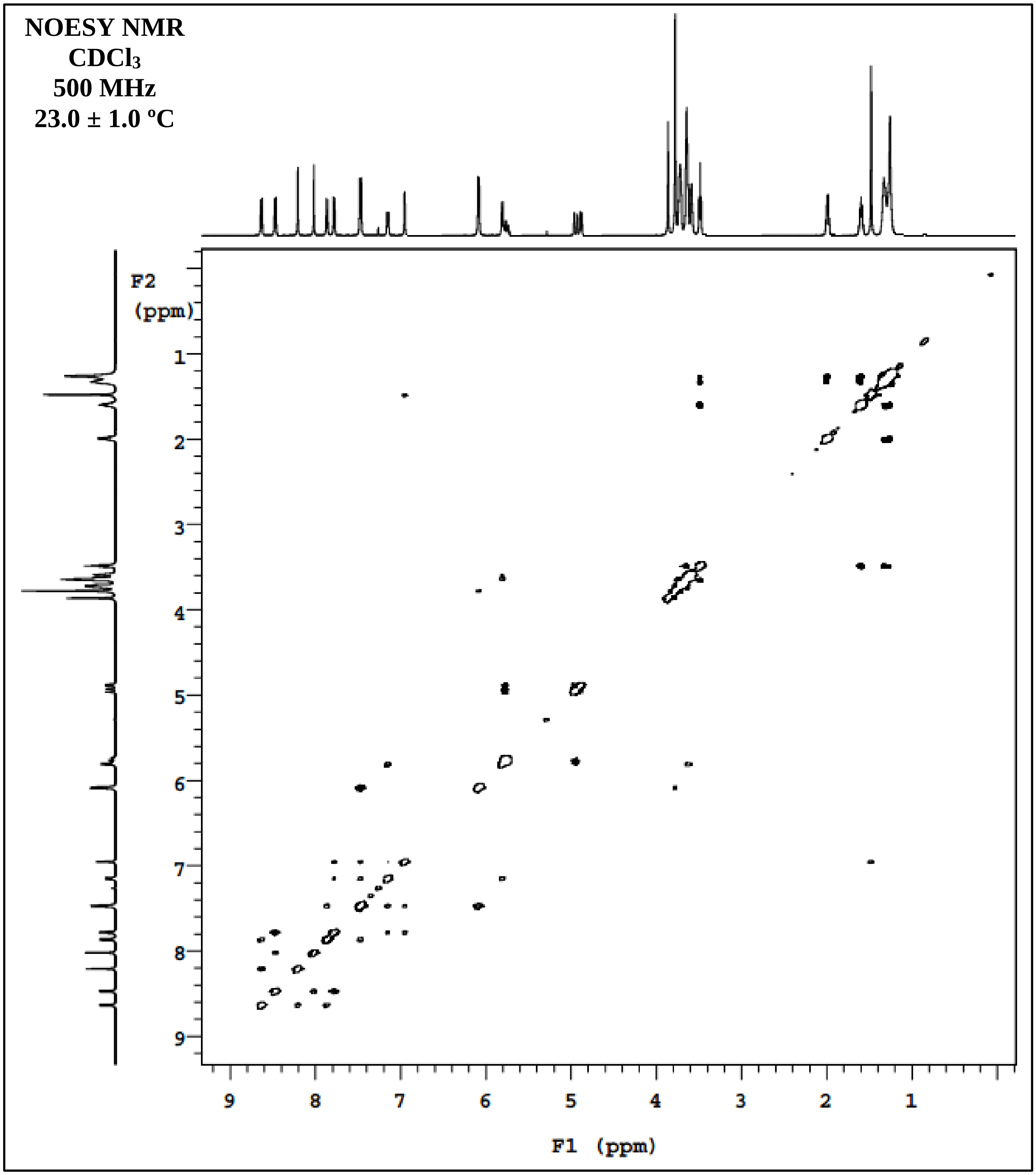

Figure S12. NOESY NMR spectrum (500 MHz, $CDCl_3$, 23 ºC) of **4**.

^{1}H NMR Spectrum of Metalated [2]Catenane 5

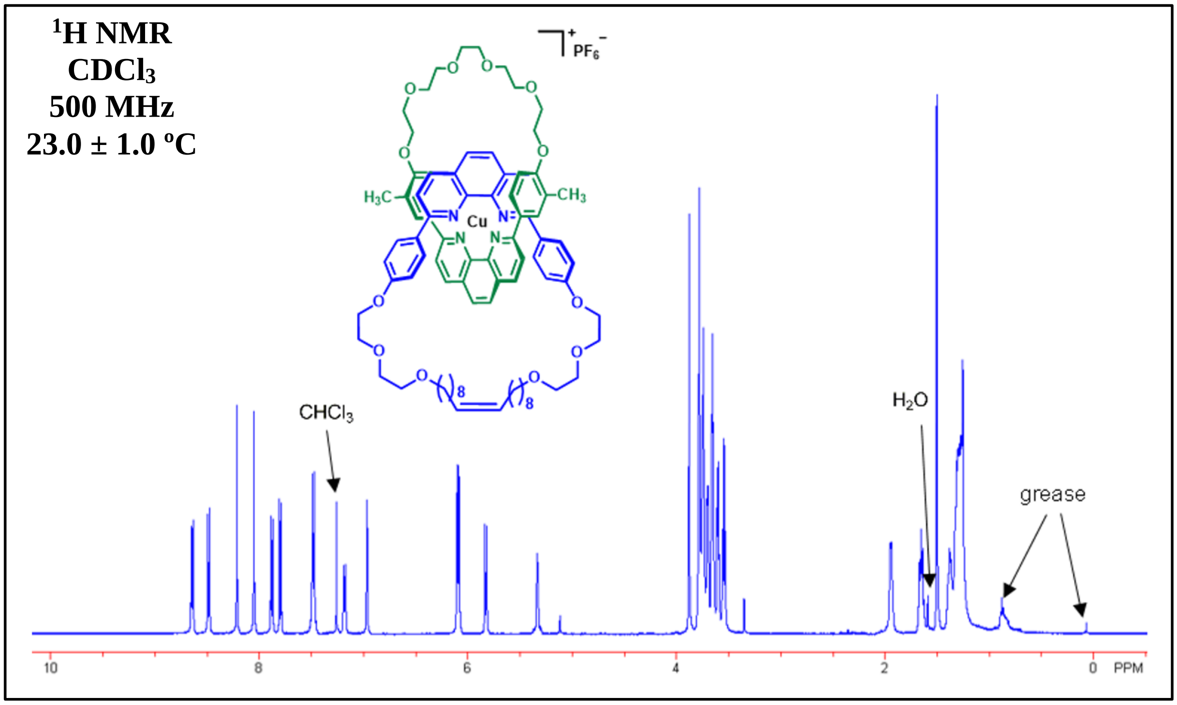

Figure S13. Annotated ^{1}H NMR spectrum (500 MHz, $CDCl_3$, 23 ºC) of **5**.

^{13}C NMR Spectrum of Metalated [2]Catenane 5

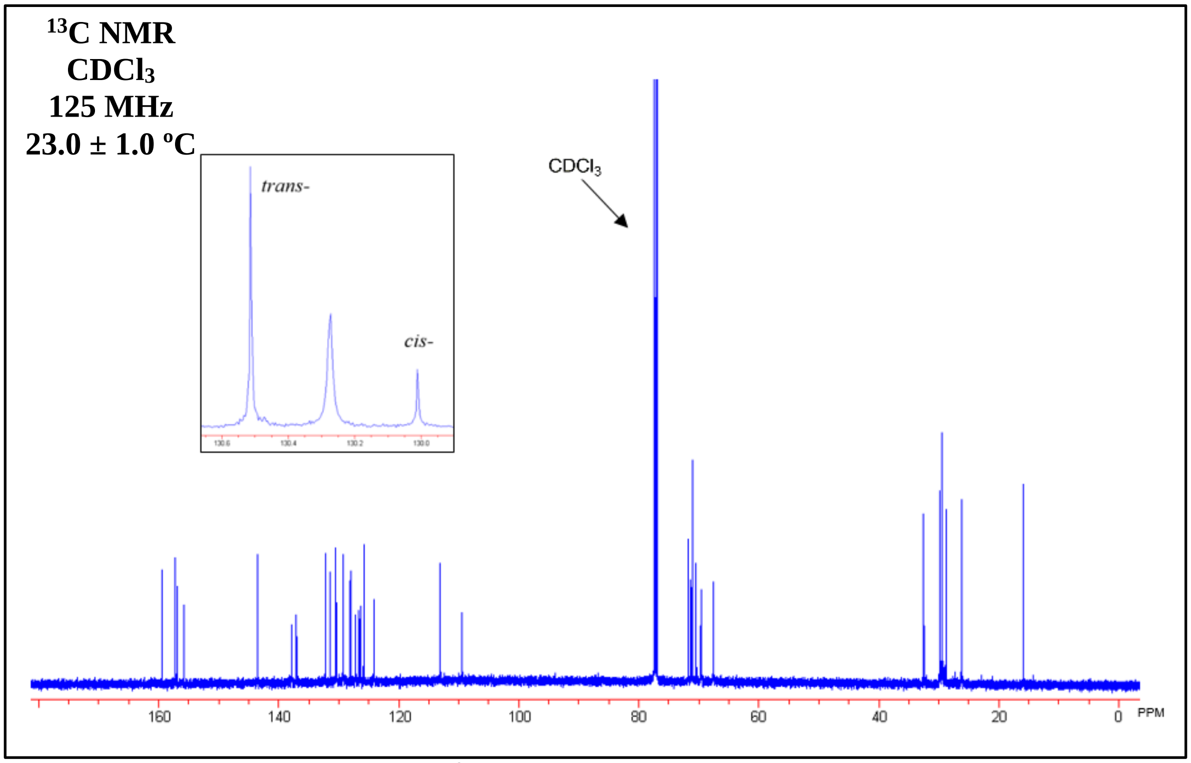

Figure S14. Annotated ^{13}C NMR spectrum (125 MHz, $CDCl_3$, 23 ºC) of **5**.

HMQC NMR Spectrum of Metalated [2]Catenane 5

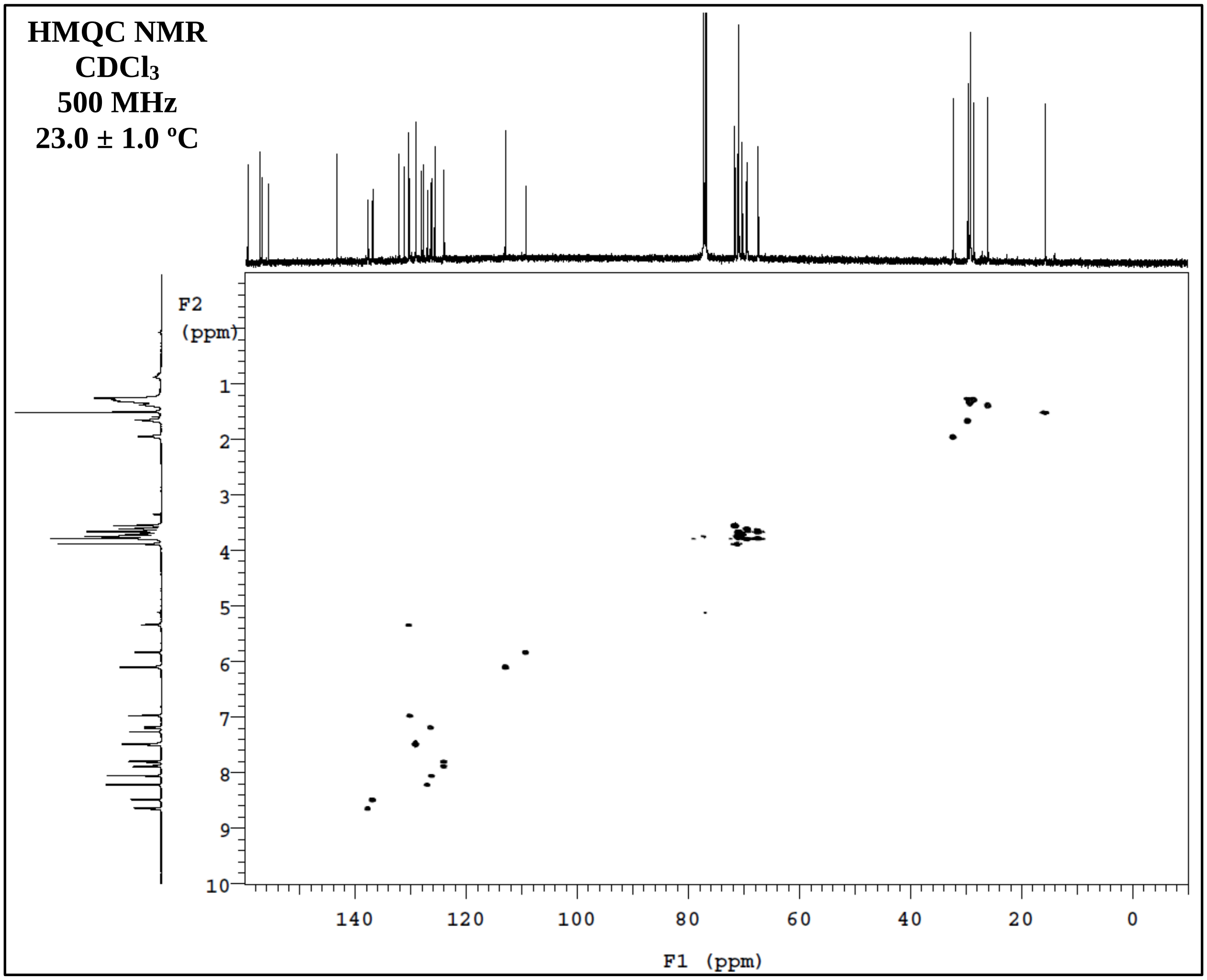

Figure S15. HMQC NMR spectrum (500 MHz, $CDCl_3$, 23 ºC) of **5**.

HMQC NMR Spectrum of Metalated [2]Catenane 5 – Expansion #1

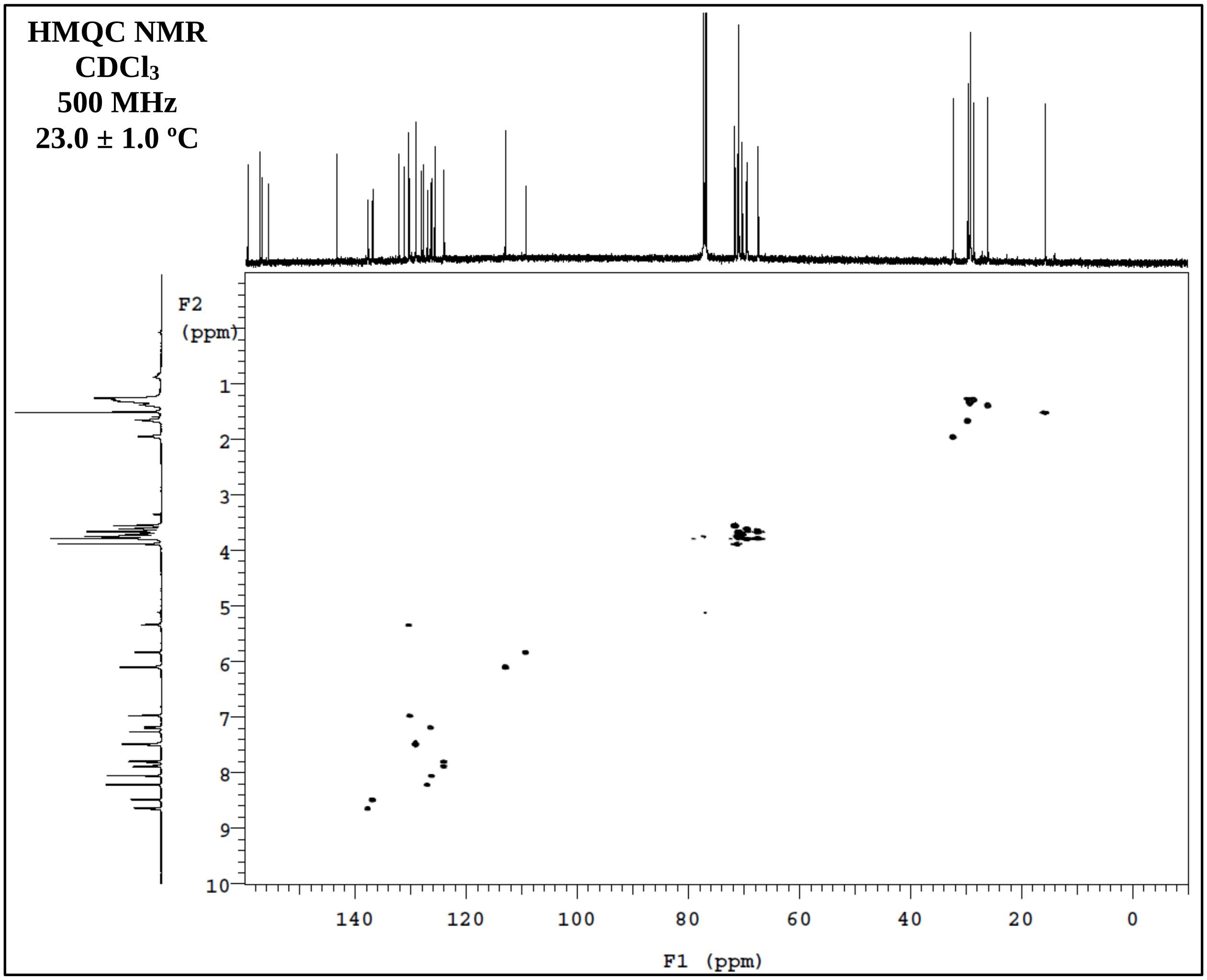

Figure S16. Expansion #1 of HMQC NMR spectrum (500 MHz, $CDCl_3$, 23 ºC) of **5**.

HMQC NMR Spectrum of Metalated [2]Catenane 5 – Expansion #2

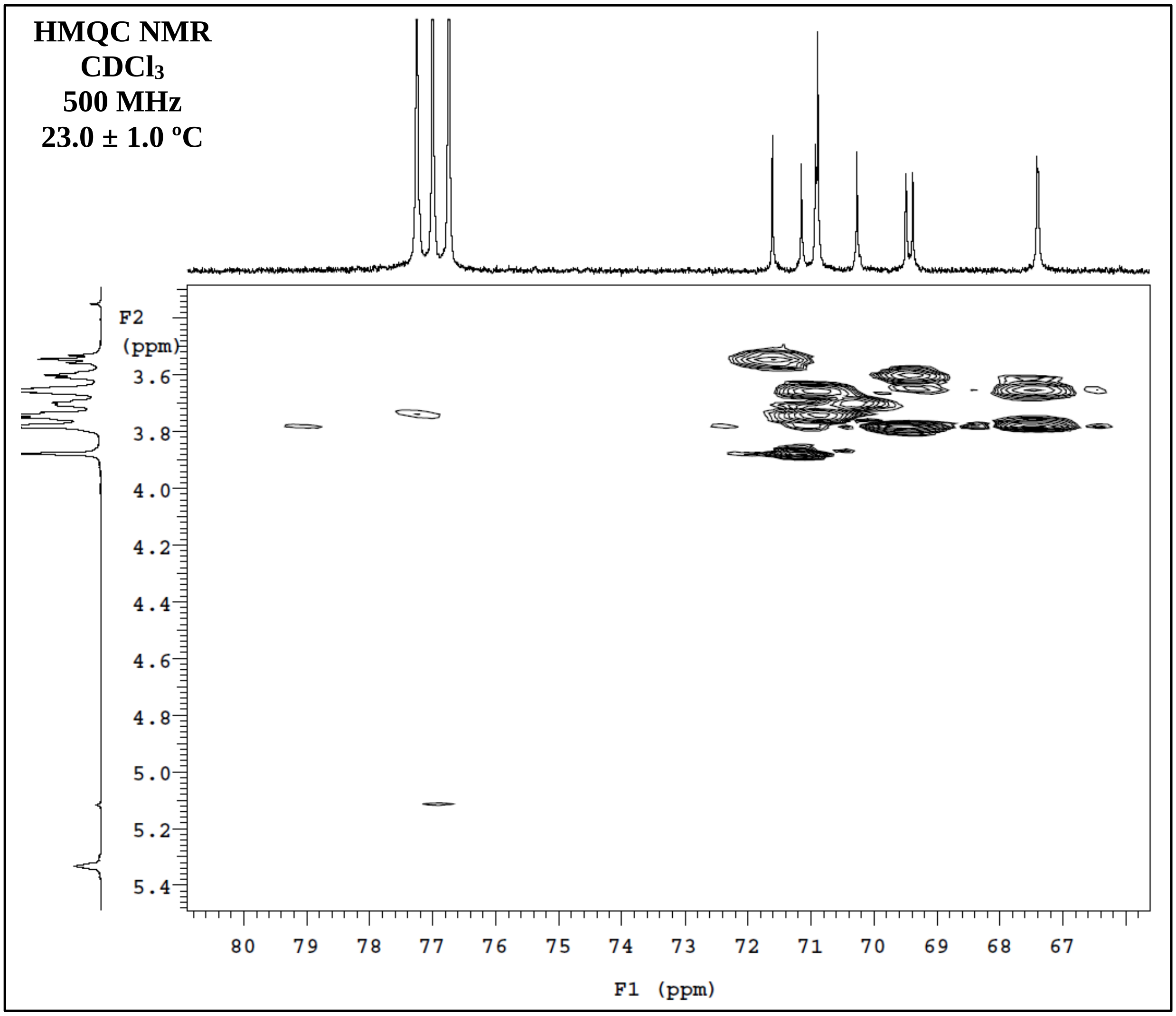

Figure S17. Expansion #2 of HMQC NMR spectrum (500 MHz, $CDCl_3$, 23 ºC) of **5**.

HMQC NMR Spectrum of Metalated [2]Catenane 5 – Expansion #3

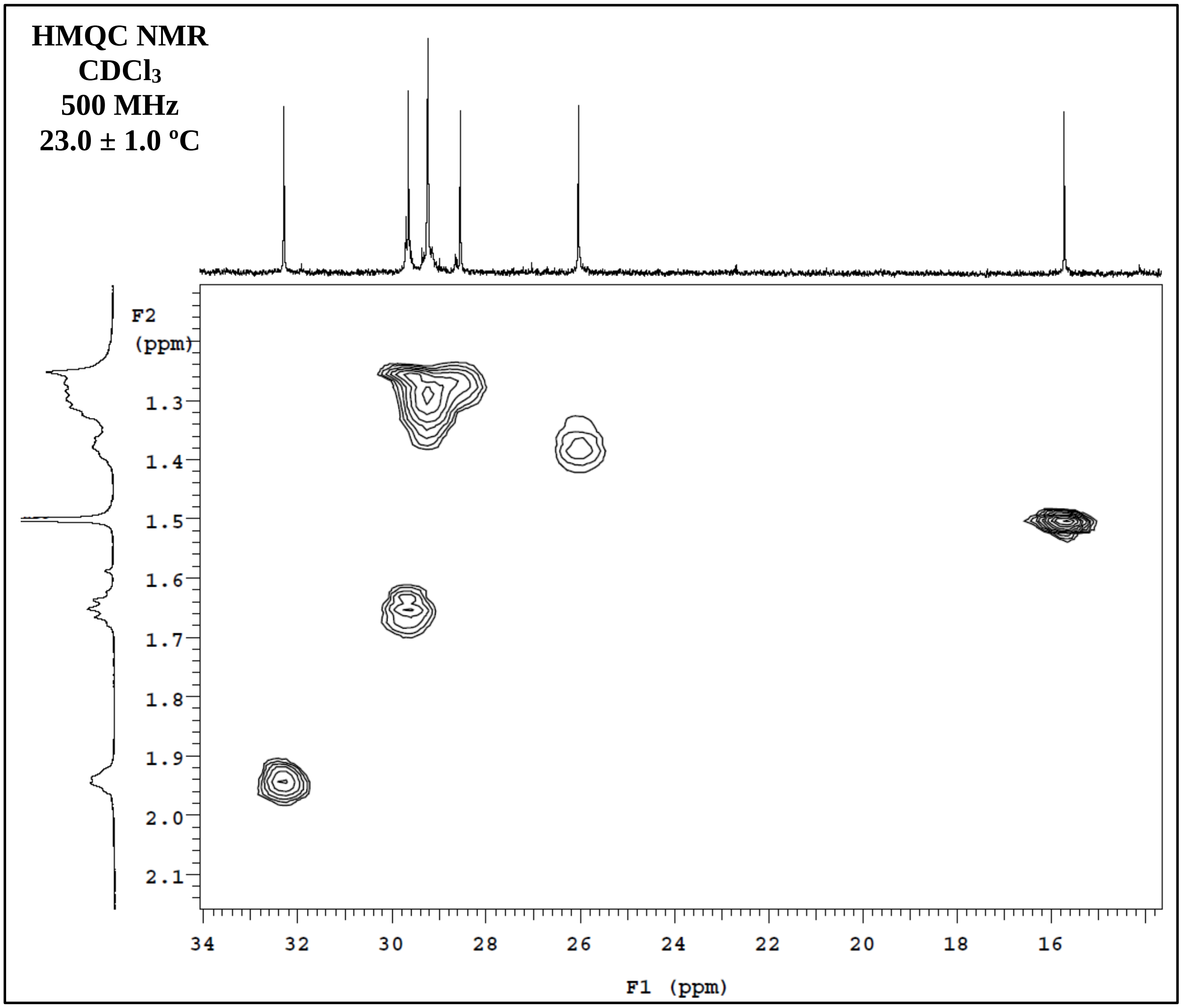

Figure S18. Expansion #3 of HMQC NMR spectrum (500 MHz, $CDCl_3$, 23 ºC) of **5**.

^{1}H NMR Spectrum of Metalated [2]Rotaxane 6

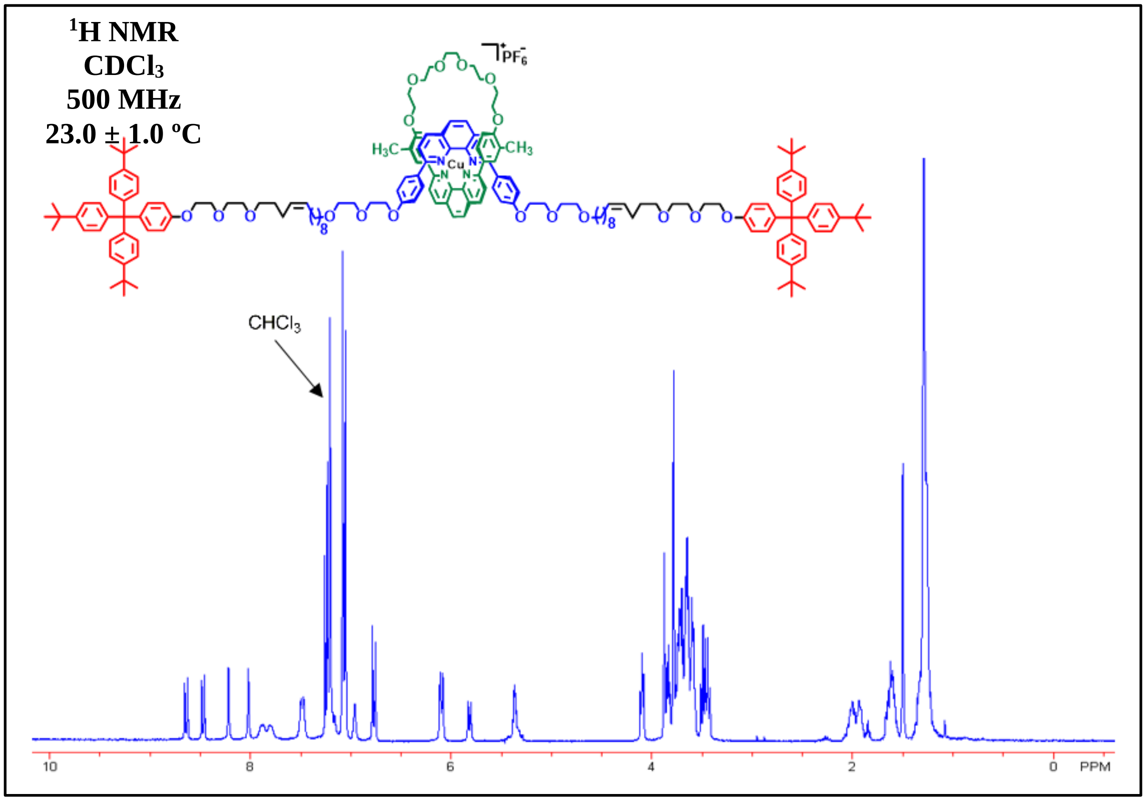

Figure S19. Annotated ^{1}H NMR spectrum (500 MHz, $CDCl_3$, 23 ºC) of **6**.

^{13}C NMR Spectrum of Metalated [2]Rotaxane 6

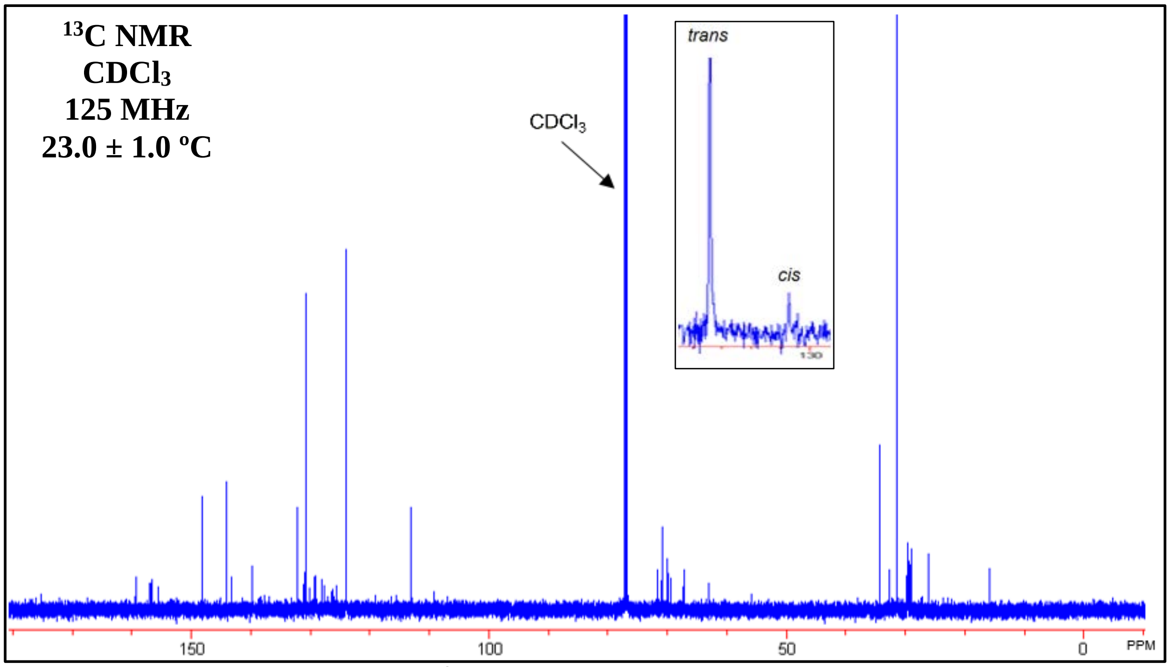

Figure S20. Annotated ^{13}C NMR spectrum (125 MHz, $CDCl_3$, 23 ºC) of **6**.

^{1}H NMR Spectrum of Core Complex 7

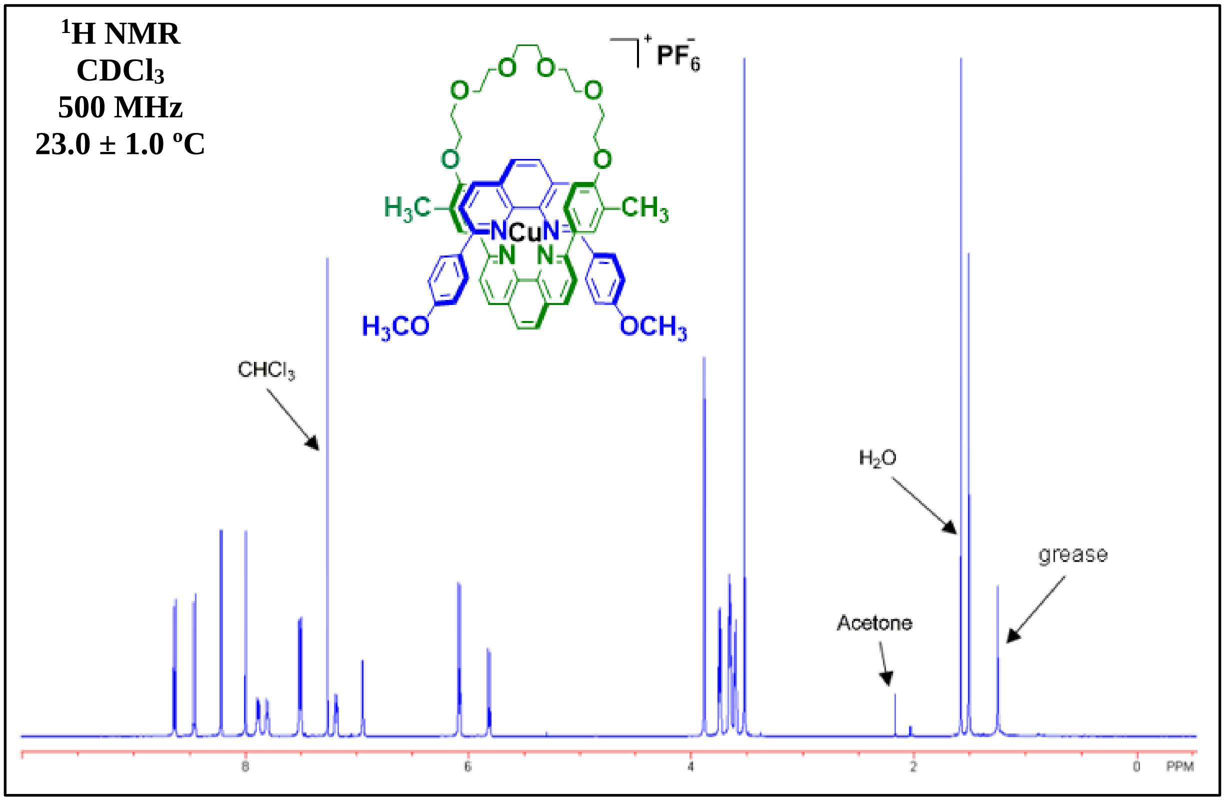

Figure S21. Annotated ^{1}H NMR spectrum (500 MHz, CDCl$_3$, 23 °C) of **7**.

^{13}C NMR Spectrum of Core Complex 7

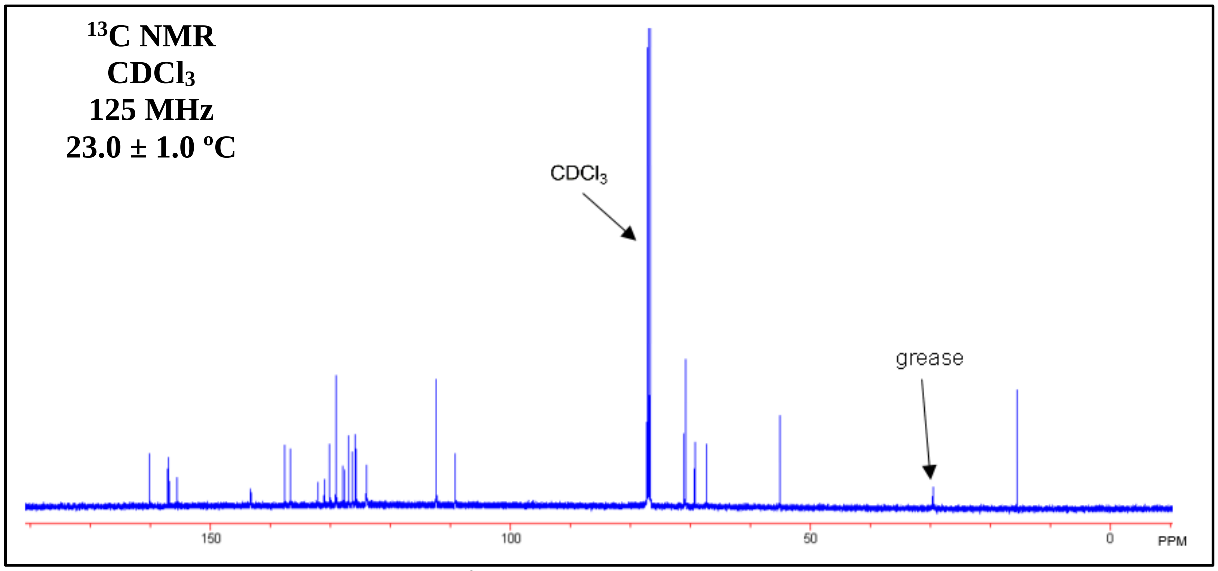

Figure S22. Annotated ^{13}C NMR spectrum (125 MHz, CDCl$_3$, 23 °C) of **7**.

Variable Temperature (VT) ^{1}H NMR Spectrum of Core Complex 7

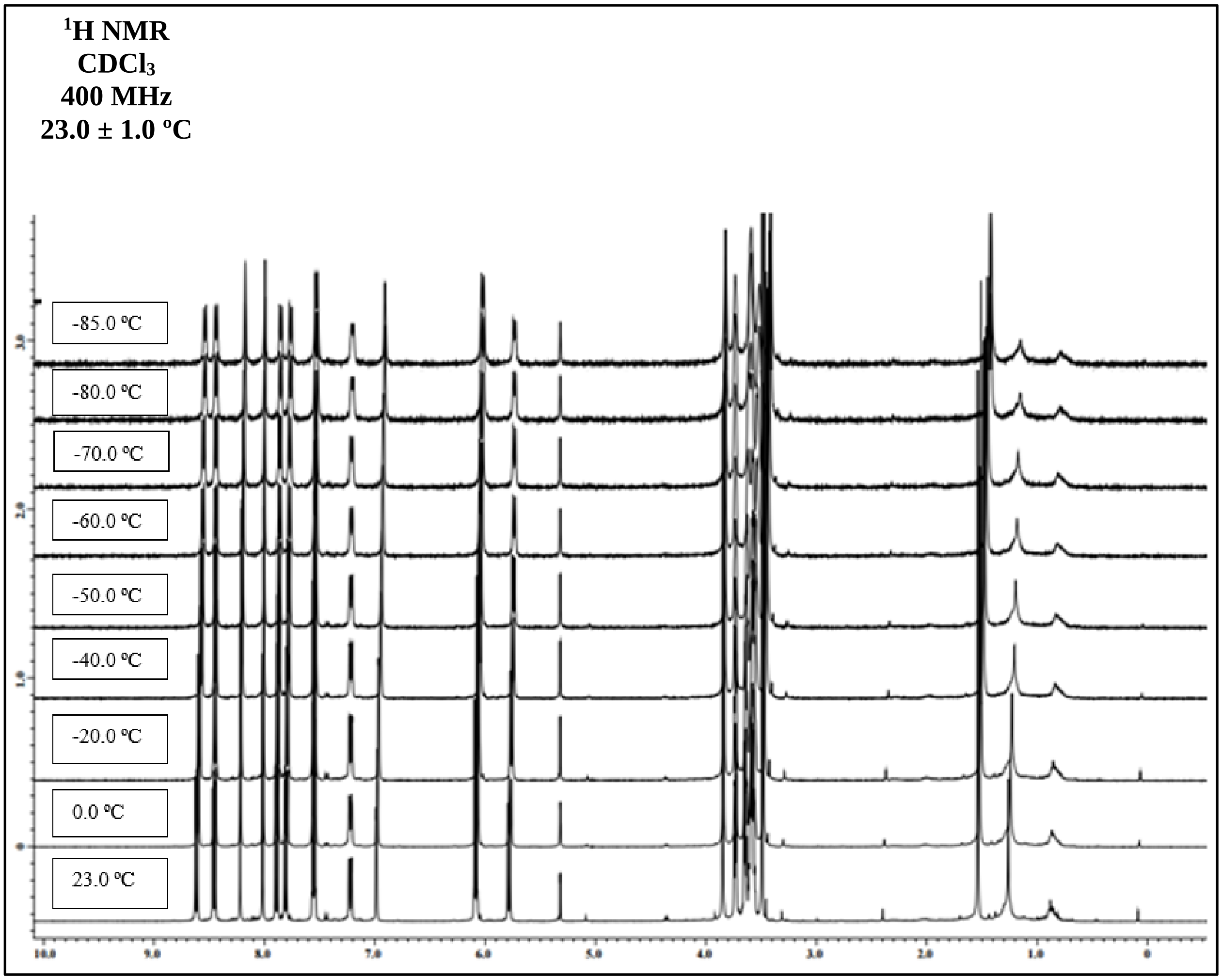

Figure S23. Stacked VT ^{1}H NMR spectrum (400 MHz, $CDCl_3$, 23 ºC) of **7**.

Variable Temperature (VT) ^{1}H NMR Spectrum of Core Complex 7 – Expansion #1

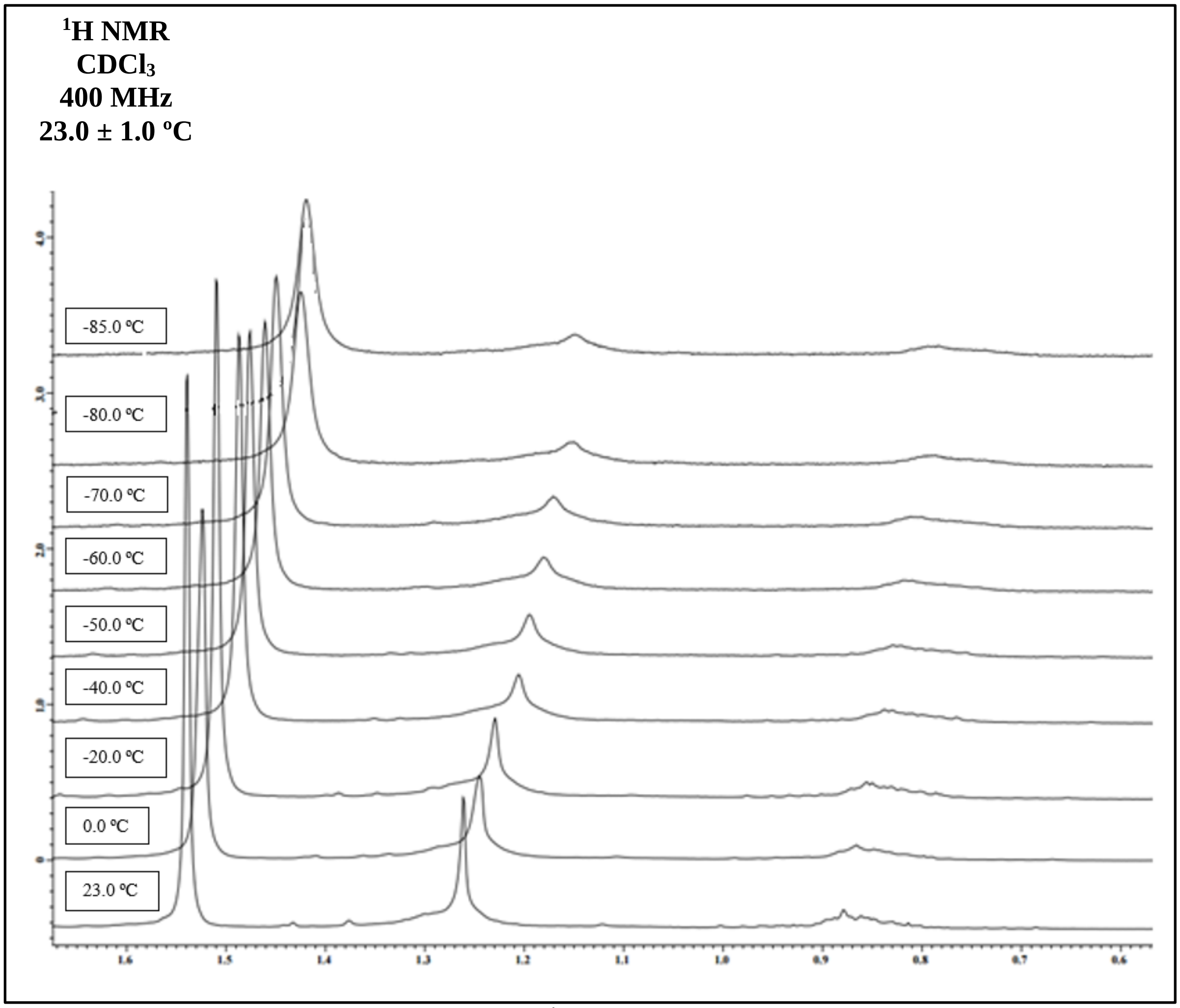

Figure S24. Expansion #1 of stacked VT ^{1}H NMR spectrum (400 MHz, $CDCl_3$, 23 ºC) of **7**.

HMQC NMR Spectrum of Core Complex 7

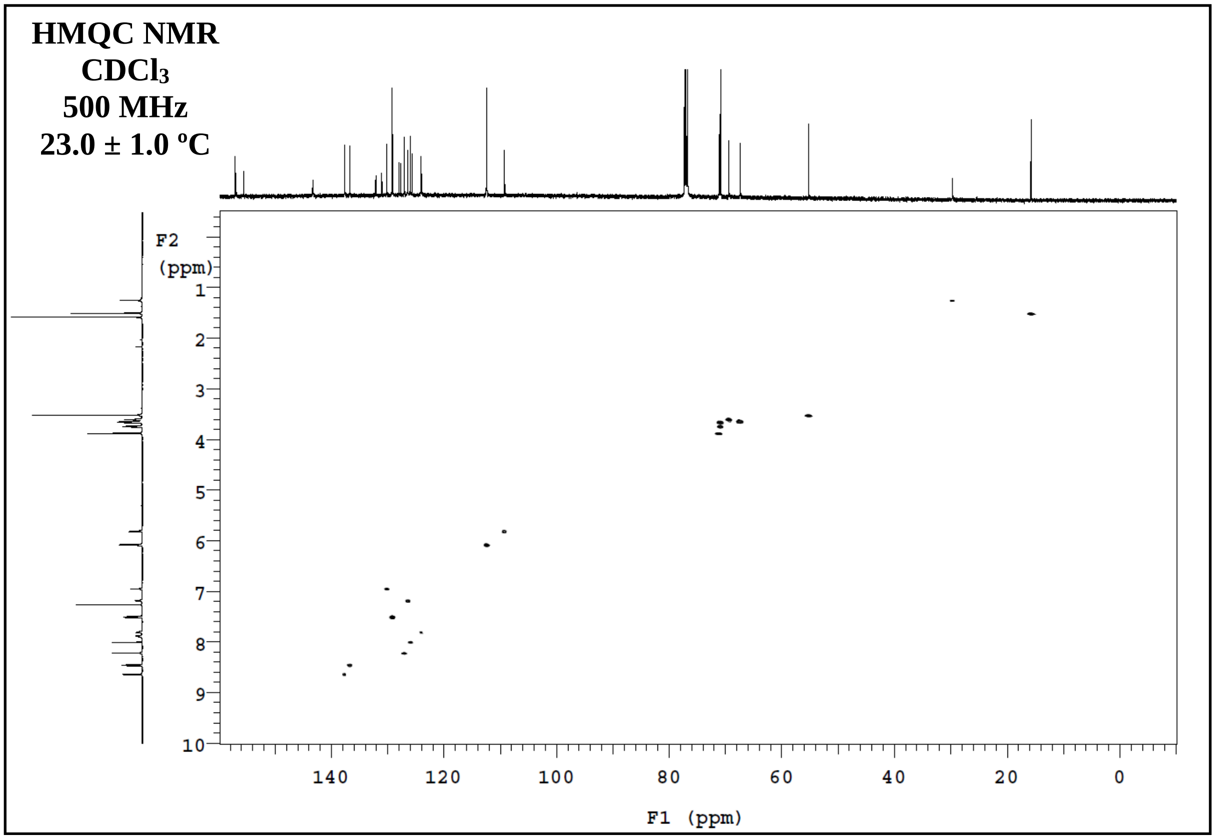

Figure S25. HMQC NMR spectrum (500 MHz, $CDCl_3$, 23 ºC) of **7**.

HMQC NMR Spectrum of Core Complex 7 – Expansion #1

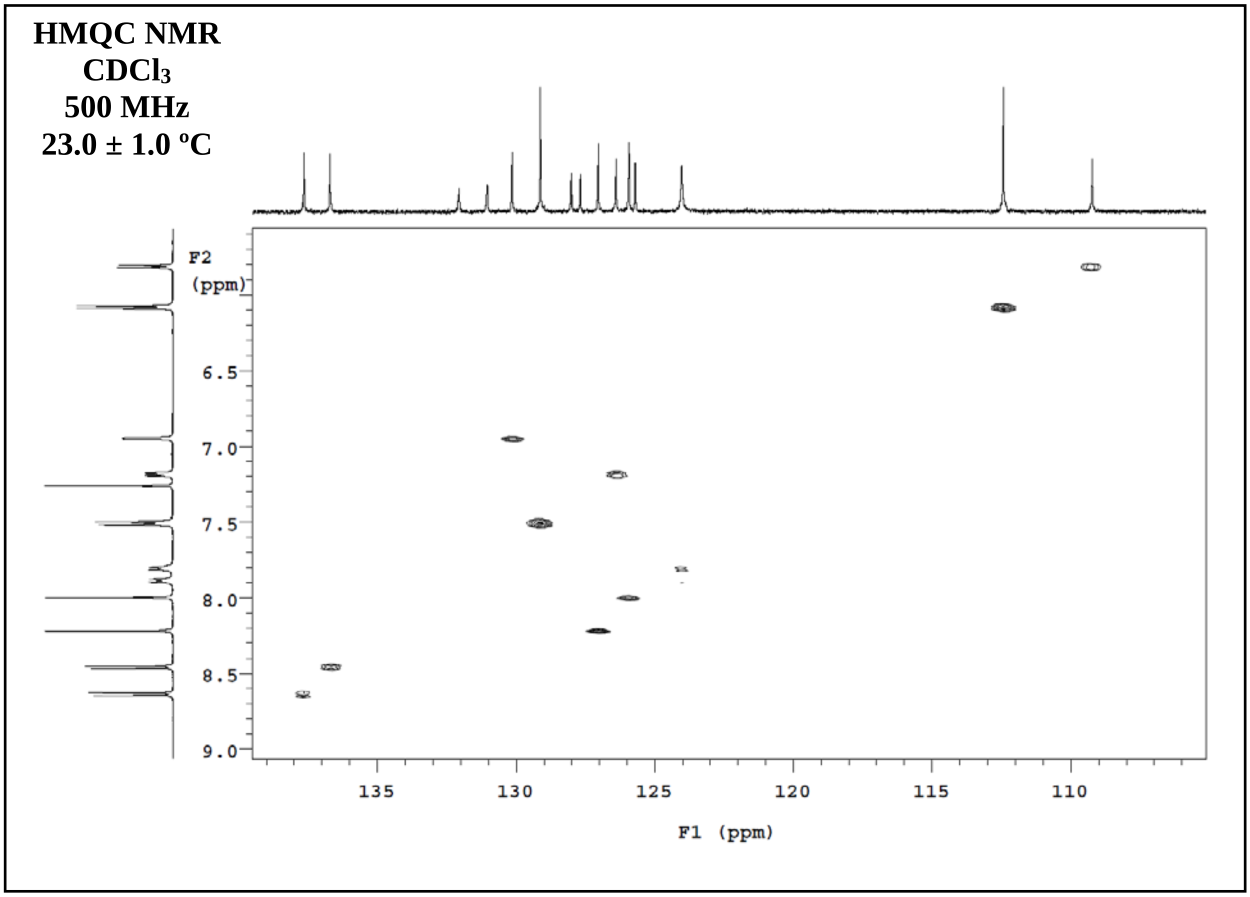

Figure S26. Expansion #1 of HMQC NMR spectrum (500 MHz, $CDCl_3$, 23 ºC) of **7**.

HMQC NMR Spectrum of Core Complex 7 – Expansion #2

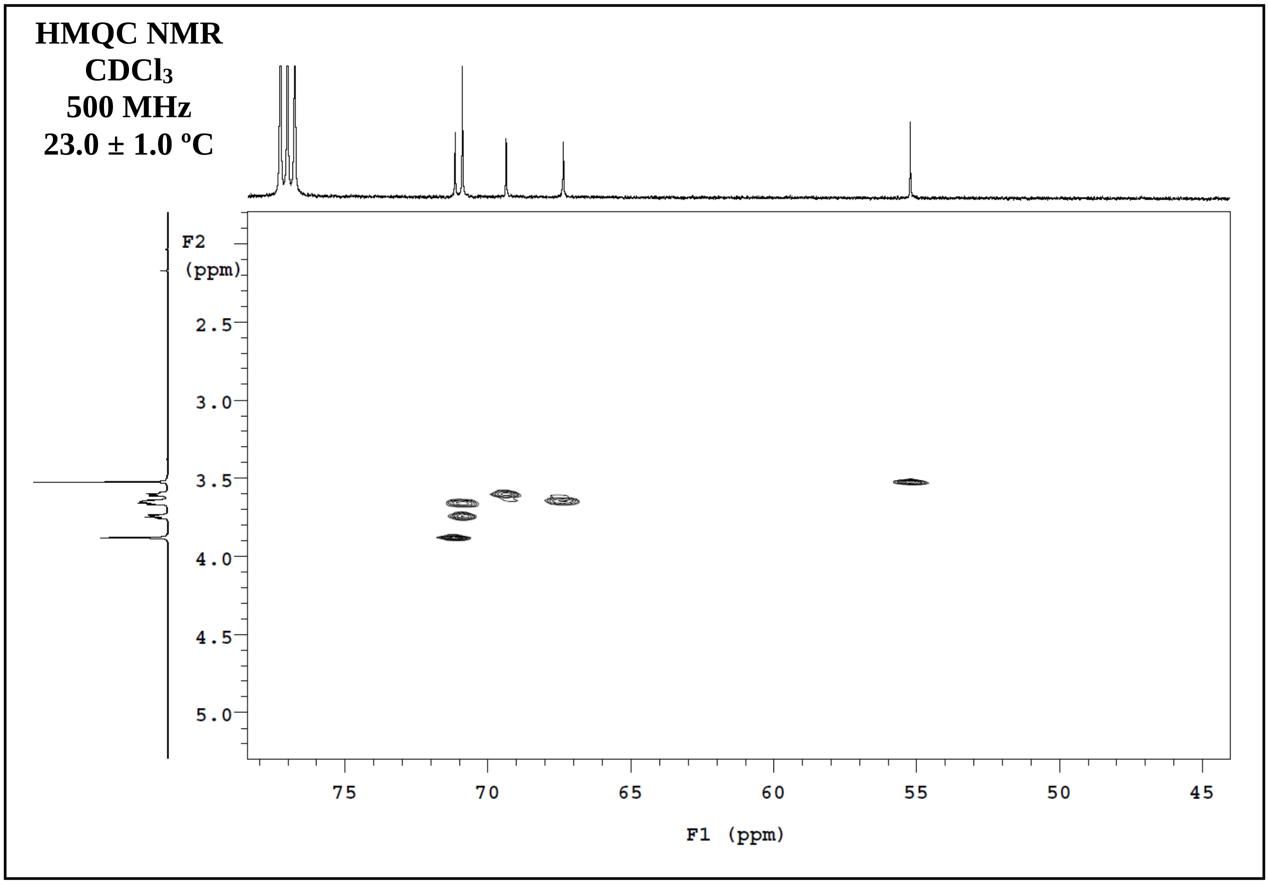

Figure S27. Expansion #2 of HMQC NMR spectrum (500 MHz, $CDCl_3$, 23 ºC) of **7**.

HMQC NMR Spectrum of Core Complex 7 – Expansion #3

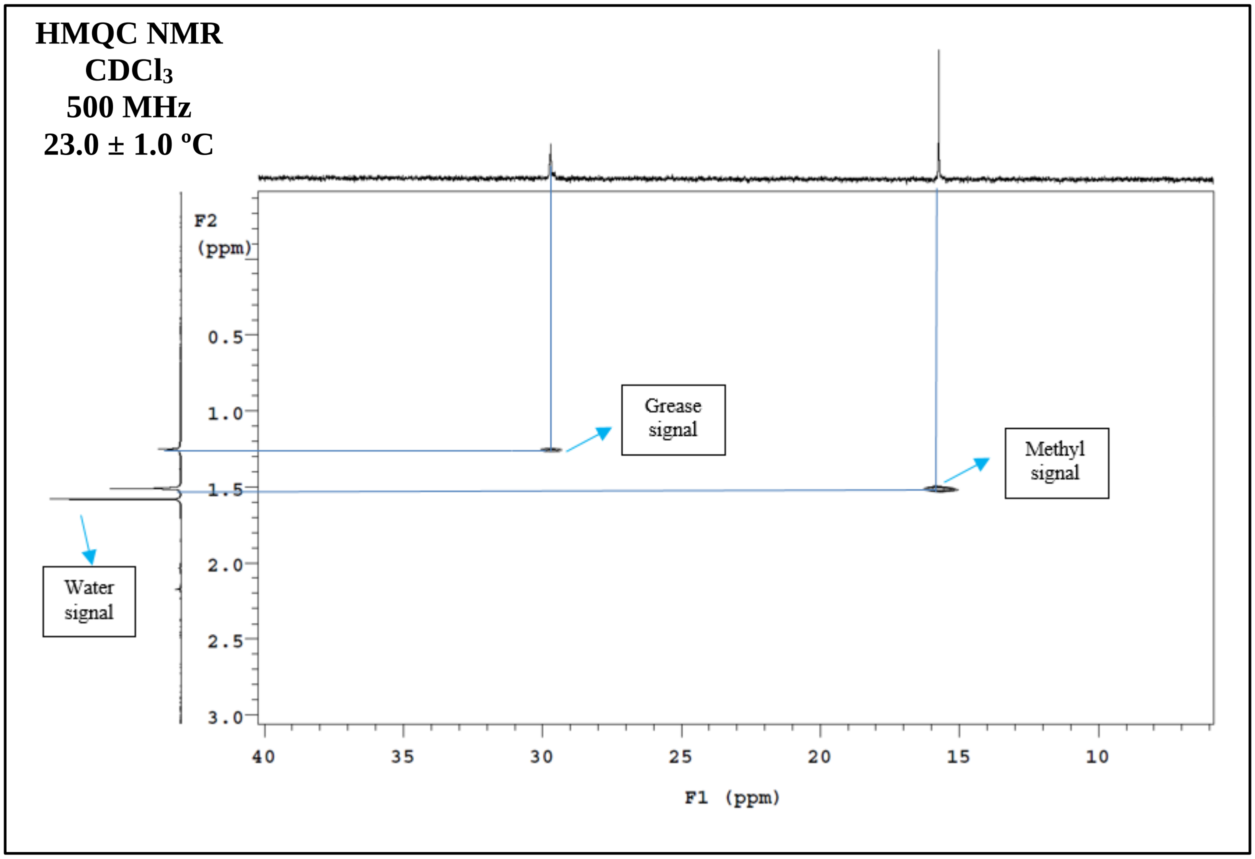

Figure S28. Expansion #3 of HMQC NMR spectrum (500 MHz, $CDCl_3$, 23 ºC) of **7**.

^{1}H NMR Spectrum of Demetalated [2]Catenane 8

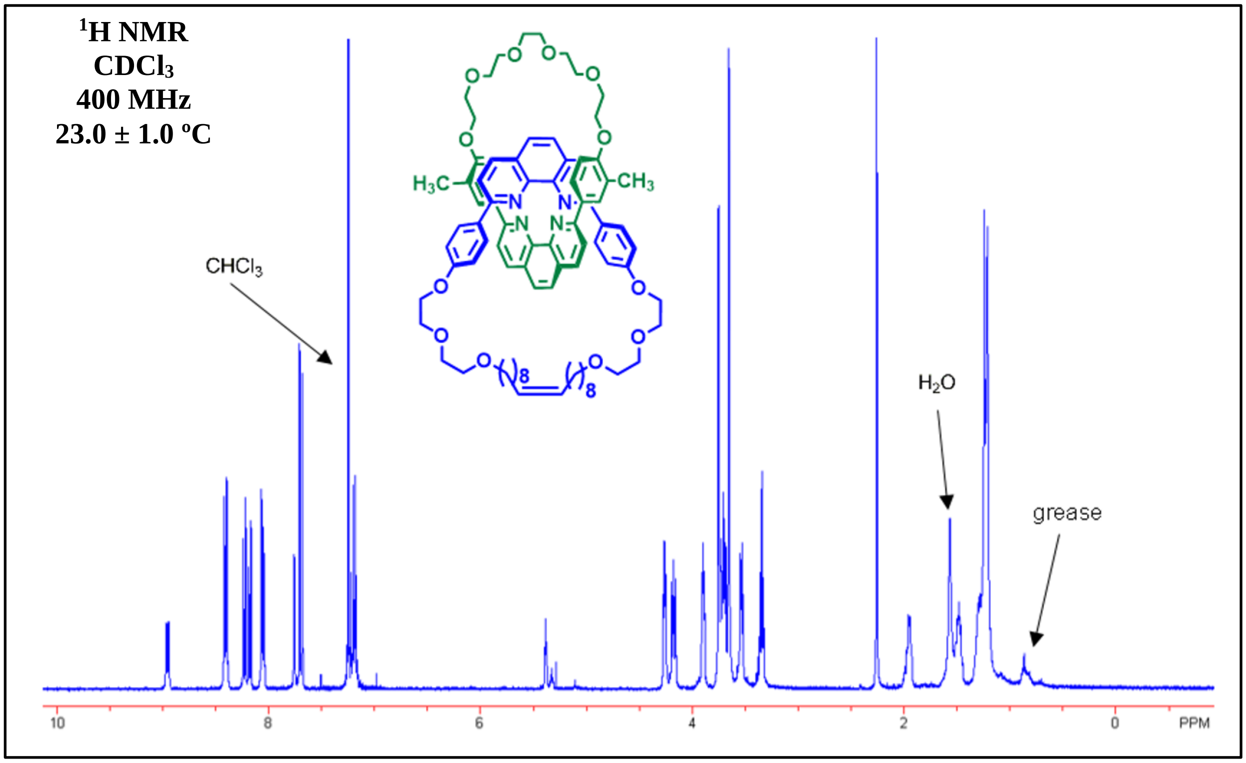

Figure S29. Annotated ^{1}H NMR spectrum (400 MHz, $CDCl_3$, 23 ºC) of **8**.

^{13}C NMR Spectrum of Demetalated [2]Catenane 8

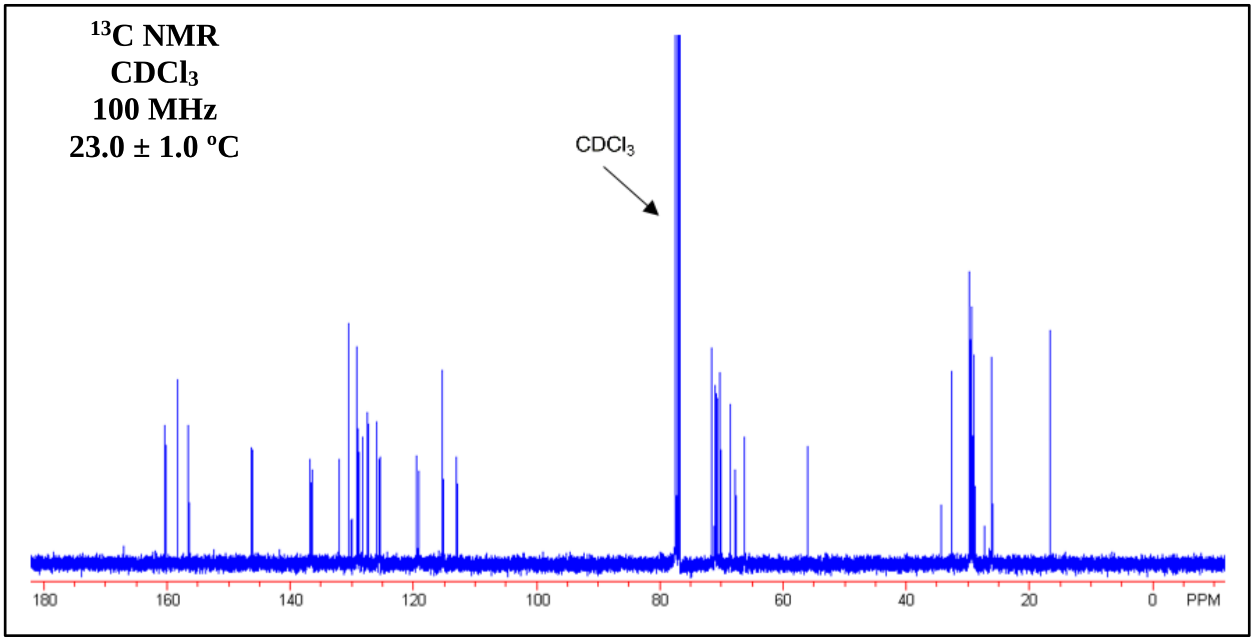

Figure S30. Annotated ^{13}C NMR spectrum (100 MHz, $CDCl_3$, 23 ºC) of **8**.

^{1}H NMR Spectrum of Demetalated [2]Rotaxane 9

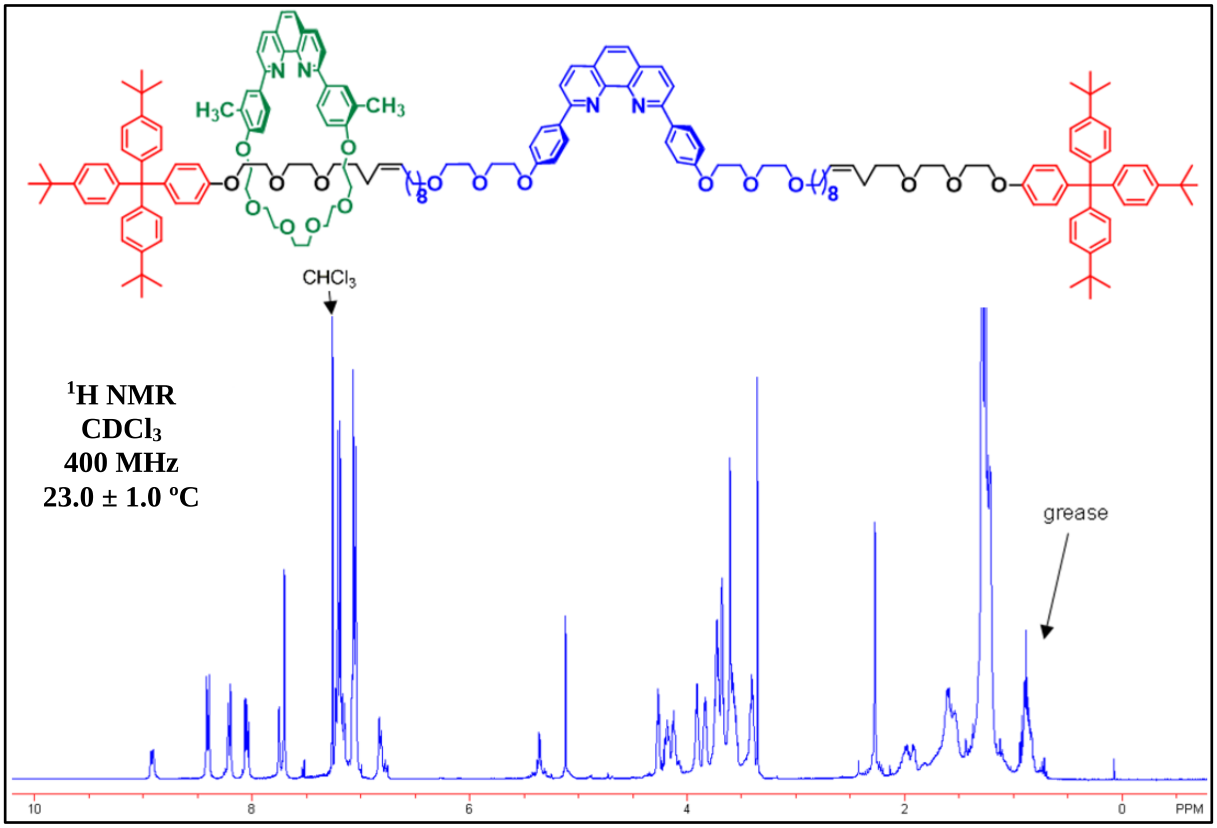

Figure S31. Annotated ^{1}H NMR spectrum (400 MHz, $CDCl_3$, 23 ºC) of **9**.

^{13}C NMR Spectrum of Demetalated [2]Rotaxane 9

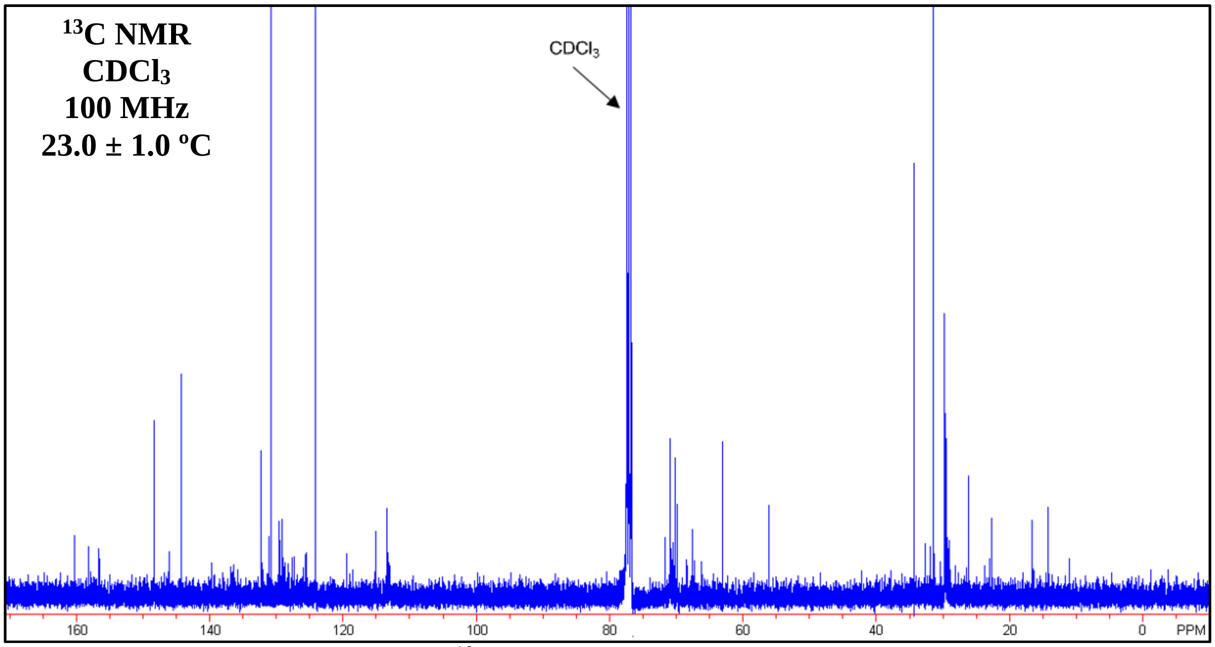

Figure S32. Annotated ^{13}C NMR spectrum (100 MHz, $CDCl_3$, 23 ºC) of **9**.

Section D. Crystallographic Characterization

X-ray diffraction data for both polymorphs of core complex **7** were collected at 173 K using a Rigaku SCXmini CCD diffractometer with a graphite monochromator [Mo Kα radiation (λ = 0.71073 Å)]. Data for both polymorphs were collected using CrystalClear[14] and processed (including correction for Lorentz, polarization and absorption) using CrysAlisPro.[15] The structures were solved by dual-space methods (SHELXT[16]) and refined by full-matrix least-squares against F^2 (SHELXL-2019/3).[17] Non-hydrogen atoms were refined anisotropically, and hydrogen atoms were refined using a riding model. The data from polymorph 2 of the complex suggested that it was twinned, and a variety of techniques were attempted during both processing and refinement to resolve the nature of this twinning and account for it appropriately in the refinement. While several potential twins could be identified, they either did not process to give data with high-quality metrics, did not refine stably, or did not show significant improvement in structure metrics. As such the data for this polymorph do still include unmodelled twinning, which leads to the elevated values of R_1 and wR_2 seen.

Due to the weak high angle diffraction both structures have their data truncated during refinement to 0.95 Å. Both structures showed disorder in some or all of the glycol chain, and rotational disorder about one F-P-F axis in the hexfluorophosphate anion. Atoms were modelled with two sites in both cases, with the minor component of the disorder isotropically refined. One *p*-methoxybenzyl group in the structure of polymorph 2 also showed disorder that could be modelled across two orientations, in this case with the minor component of the disorder anisotropically refined. All of the disorder in both structures was refined with geometric and thermal restraints applied. All calculations were performed using the Olex2[18] interface. Selected crystallographic data are presented in **Table S1**.

CCDC 967317 and 2469972 contains the supplementary crystallographic data for this paper. These data can be obtained free of charge from The Cambridge Crystallographic Data Centre *via* www.ccdc.cam.ac.uk/structures.

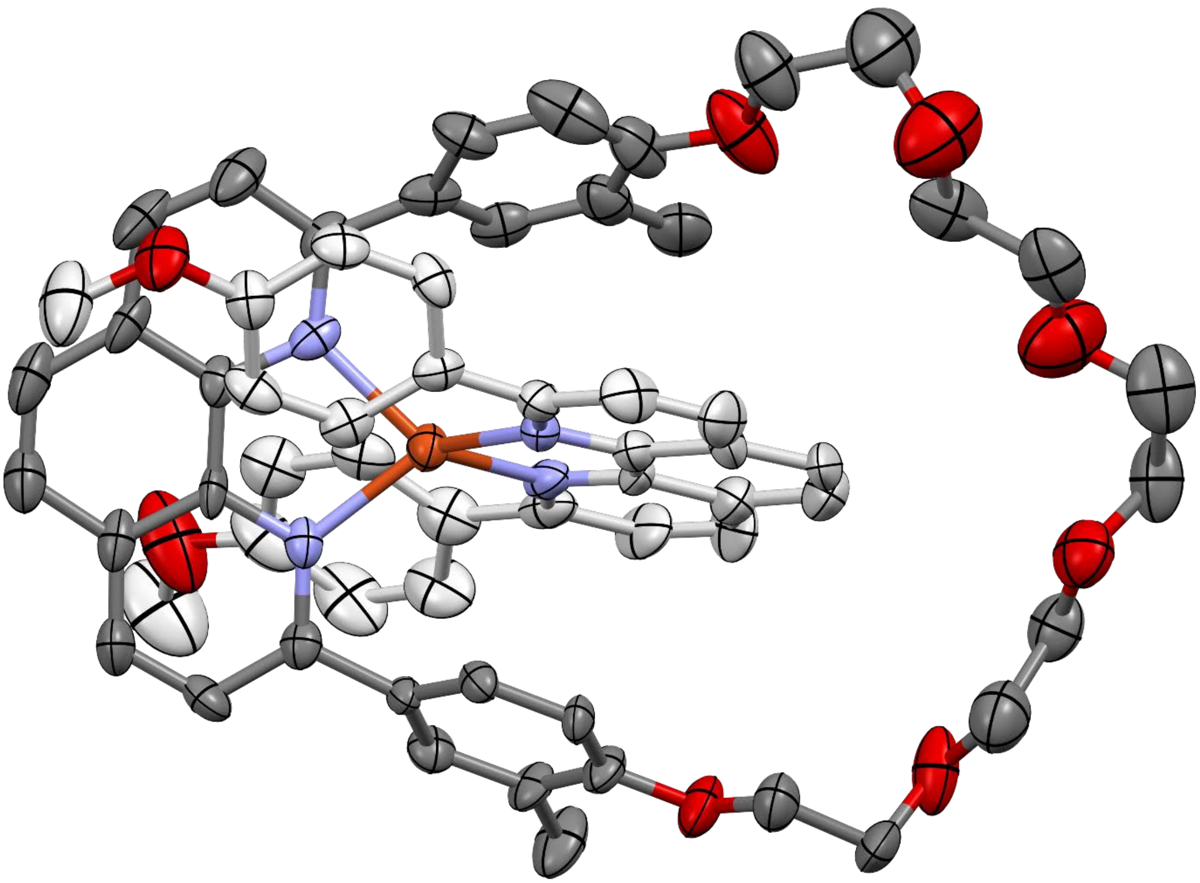

Figure S33. View of the solid state structure of the first polymorph (1) of core complex **7**, hydrogen atoms, anions, and the minor component of disorder were omitted for clarity, ellipsoids are drawn at the 50% probability level. (Cu: orange, O: red, N: blue, C: light or dark gray).

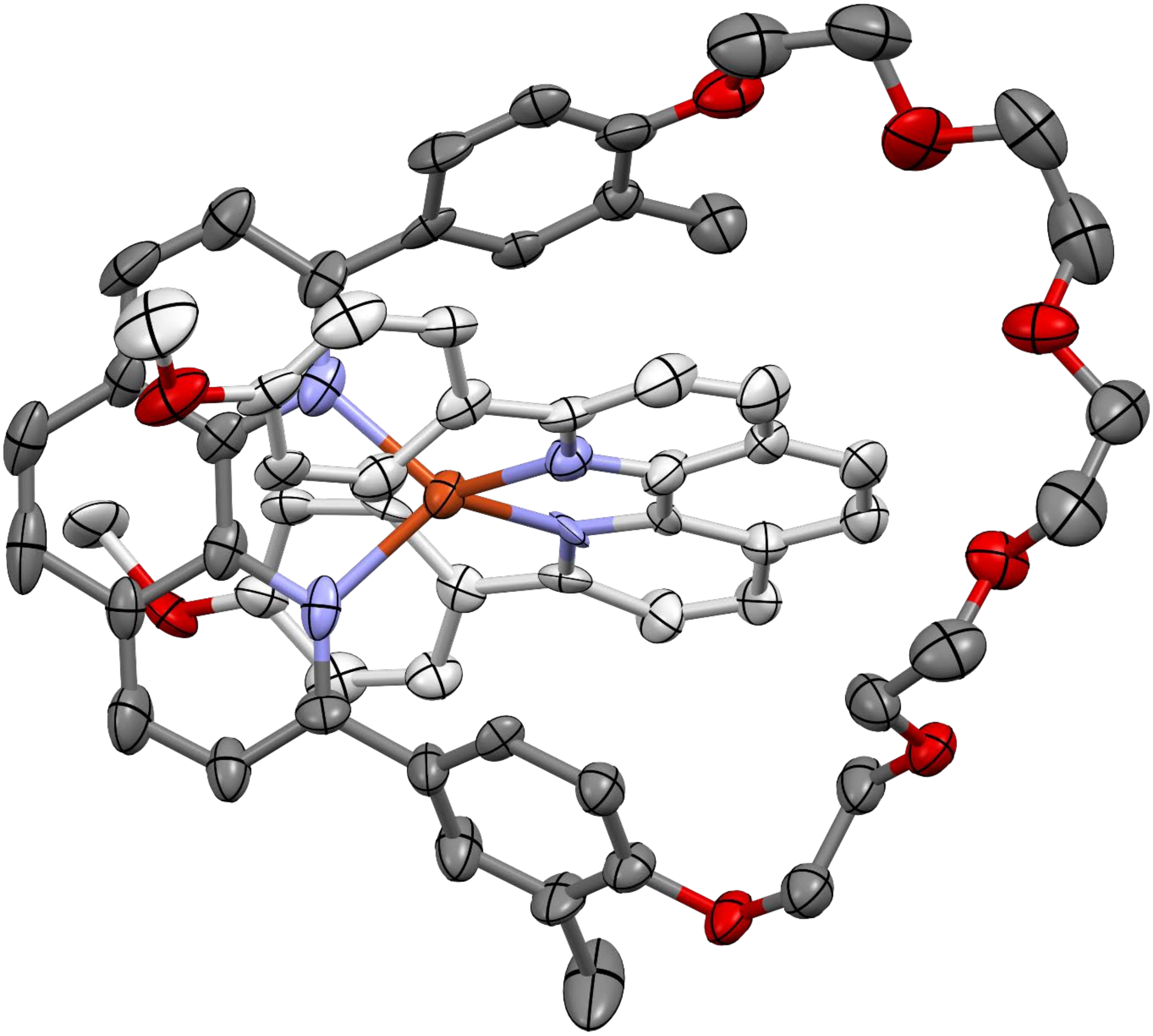

Figure S34. View of the solid state structure of the second polymorph (2) of the core complex **7**, hydrogen atoms, anions, and minor component of disorder were omitted for clarity, ellipsoids are drawn at the 50% probability level.

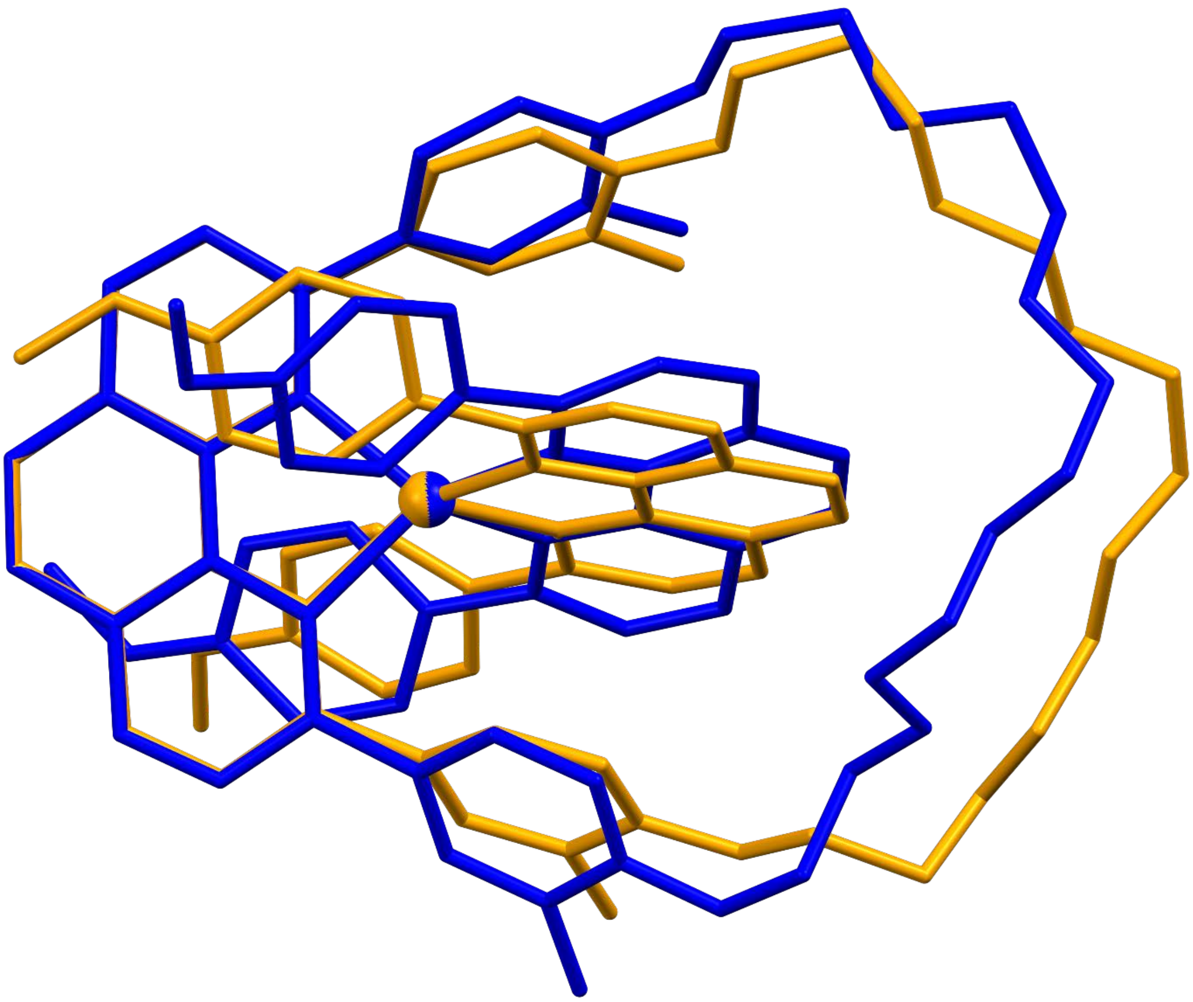

Figure S35. View showing the overlay of the solid state structures of the two polymorphs 1 (*gold*) and 2 (*blue*) of core complex **7**.

Table S1. Crystallographic data and structure refinement for compound **7**.

	Core Complex **7** polymorph 1 (CCDC 967317)	Core Complex **7** polymorph 2 (CCDC 2469972)
**Empirical Formula**	$C_{62}H_{58}CuF_6N_4O_8P$	$C_{62}H_{58}CuF_6N_4O_8P$
**Mol. Weight**	1195.63	1195.63
**Temperature / K**	173(2)	173(2)
**Crystal System**	triclinic	monoclinic
**Space Group**	$P\bar{1}$ (no. 2)	$P2_1/c$ (no. 14)
a **(Å)**	14.7809(14)	16.7265(8)
b **(Å)**	14.8375(16)	7.8498(4)
c **(Å)**	15.4905(18)	43.035(2)
α **(°)**	115.129(11)	
β **(°)**	114.546(10)	96.345(5)
γ **(°)**	92.324(8)	
Volume **(Å^3)**	2698.9(6)	5615.8(5)
Z	2	4
**ρ_{calc} / g cm^{-3}**	1.471	1.414
**μ / mm^{-1}**	0.518	0.498
**F(000)**	1240	2480
**Crystal Size / mm^3**	0.09 × 0.06 × 0.02	0.42×0.12×0.02
**Crystal description**	Red prism	Red plate
**Reflections Collected**	16972	33616
**Independent Reflections**	6604 [R(int) = 0.1299]	6867 [R(int) = 0.0900]
**Parameters, restraints**	801, 441	834, 420
**GoF on *F*^{2}**	1.004	1.259
**Final R Indexes [I>2σ (I)]**	R_1 = 0.0754, wR_2 = 0.1168	R_1 = 0.1472, wR_2 = 0.3117
**Final R Indexes (all data)**	R_1 = 0.1699, wR_2 = 0.1548	R_1 = 0.1679, wR_2 = 0.3205
**Largest difference peak and hole**	0.589, -0.460	0.753, -1.4188

Section E. Size-Exclusion Chromatograms (SECs)

SEC Chromatogram of Macrocycle 1

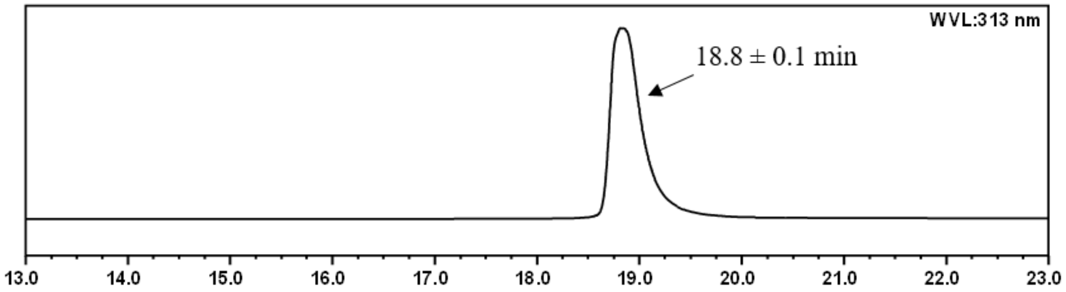

Figure S36. Size-Exclusion Chromatogram (SEC) of **1**.

SEC Chromatogram of Mono-stoppered Olefin 2

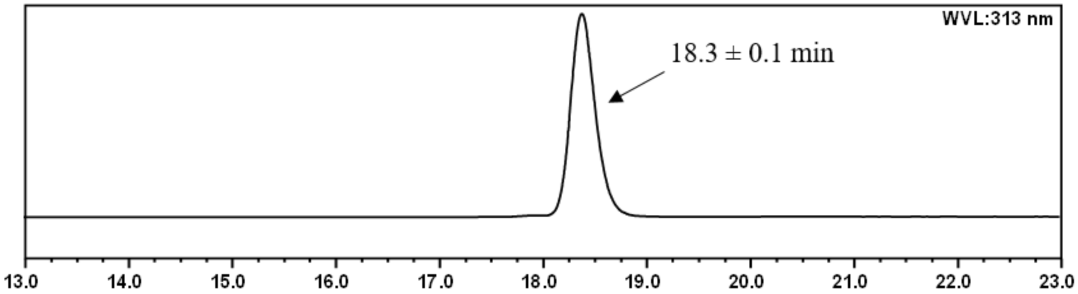

Figure S37. Size-Exclusion Chromatogram (SEC) of **2**.

SEC Chromatogram of Di-stoppered Olefin 3

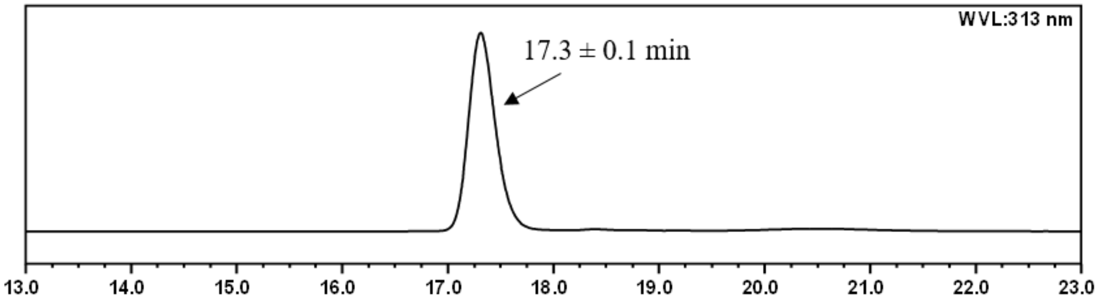

Figure S38. Size-Exclusion Chromatogram (SEC) of **3**.

SEC Chromatogram of Metalated [2]Pseudorotaxane 4

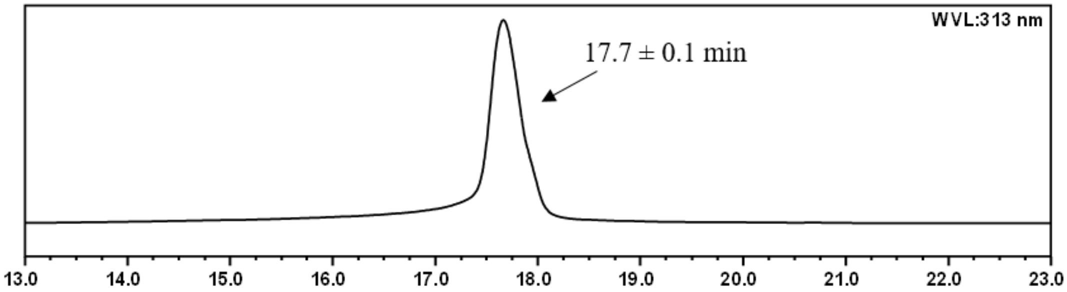

Figure S39. Size-Exclusion Chromatogram (SEC) of **4**.

SEC Chromatogram of Metalated [2]Catenane 5

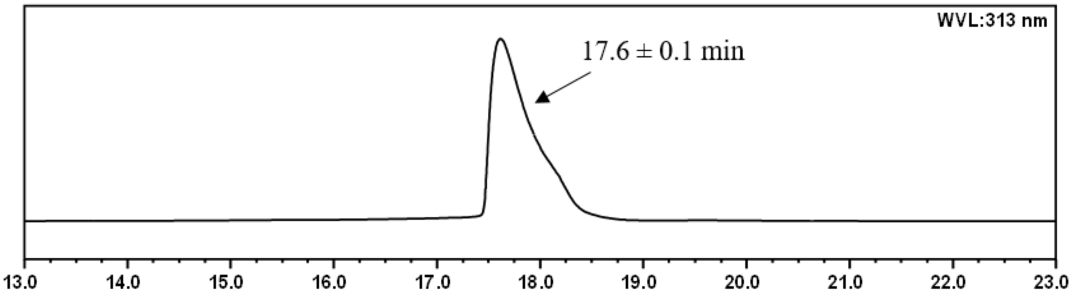

Figure S40. Size-Exclusion Chromatogram (SEC) of **5**.

SEC Chromatogram of Metalated [2]Rotaxane 6

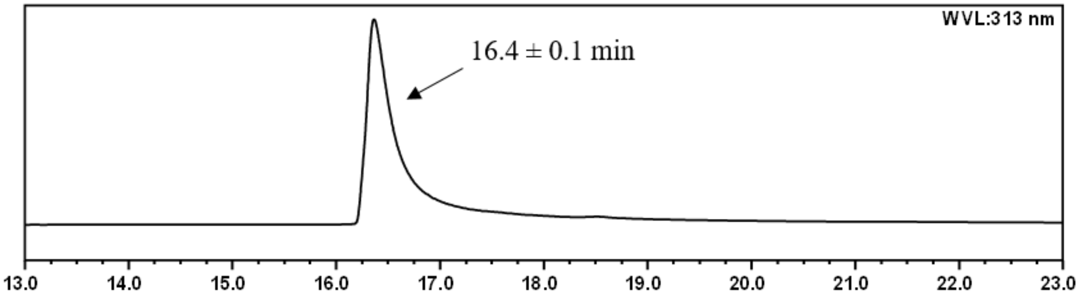

Figure S41. Size-Exclusion Chromatogram (SEC) of **6**.

SEC Chromatogram of Core Complex 7

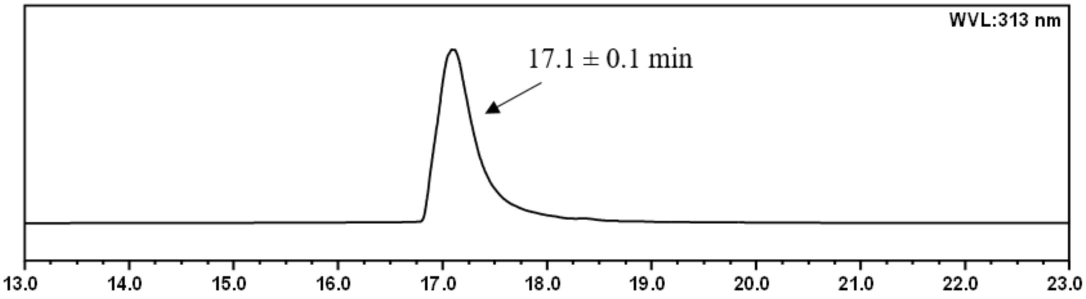

Figure S42. Size-Exclusion Chromatogram (SEC) of **7**.

SEC Chromatogram of Demetalated [2]Catenane 8

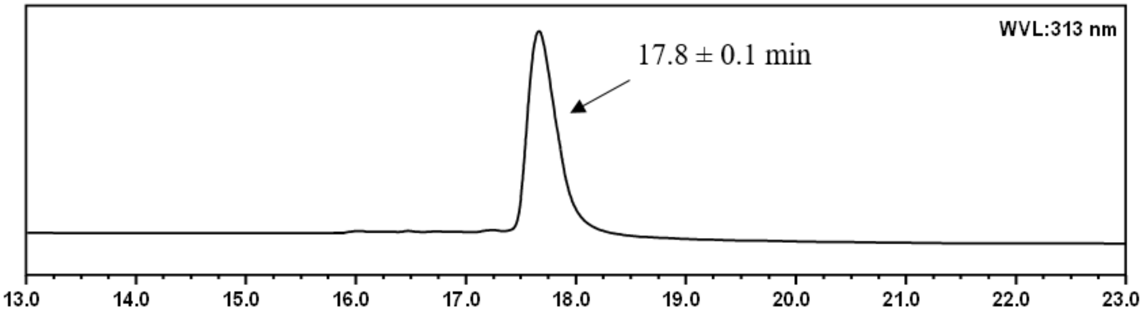

Figure S43. Size-Exclusion Chromatogram (SEC) of **8**.

SEC Chromatogram of Demetalated [2]Rotaxane 9

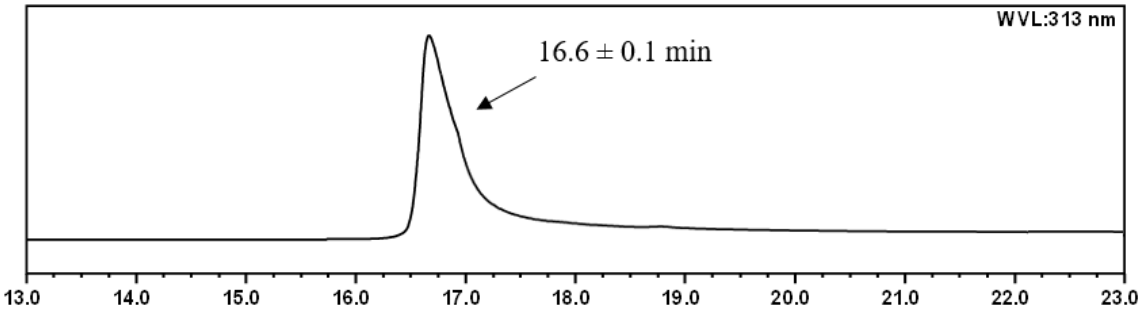

Figure S44. Size-Exclusion Chromatogram (SEC) of **9**.

Section F. Additional Notes

Upon the synthesis of copper (I) complexes of core complex, [2]pseudorotaxane, [2]catenane, and [2]rotaxane, copper (I) was oxidized to copper (II). In order to address this oxidation problem, sodium dithionite (90%) was used in the presence of 2N aqueous NaOH solution. Copper (II) was successfully reduced to copper (I) without causing any issues with the structures of these complexes. A representing examples of ^{1}H NMR spectra of oxidized (**Figure S45**) and reduced (**Figure S46**) copper complex of [2]catenane **5** was shown below.

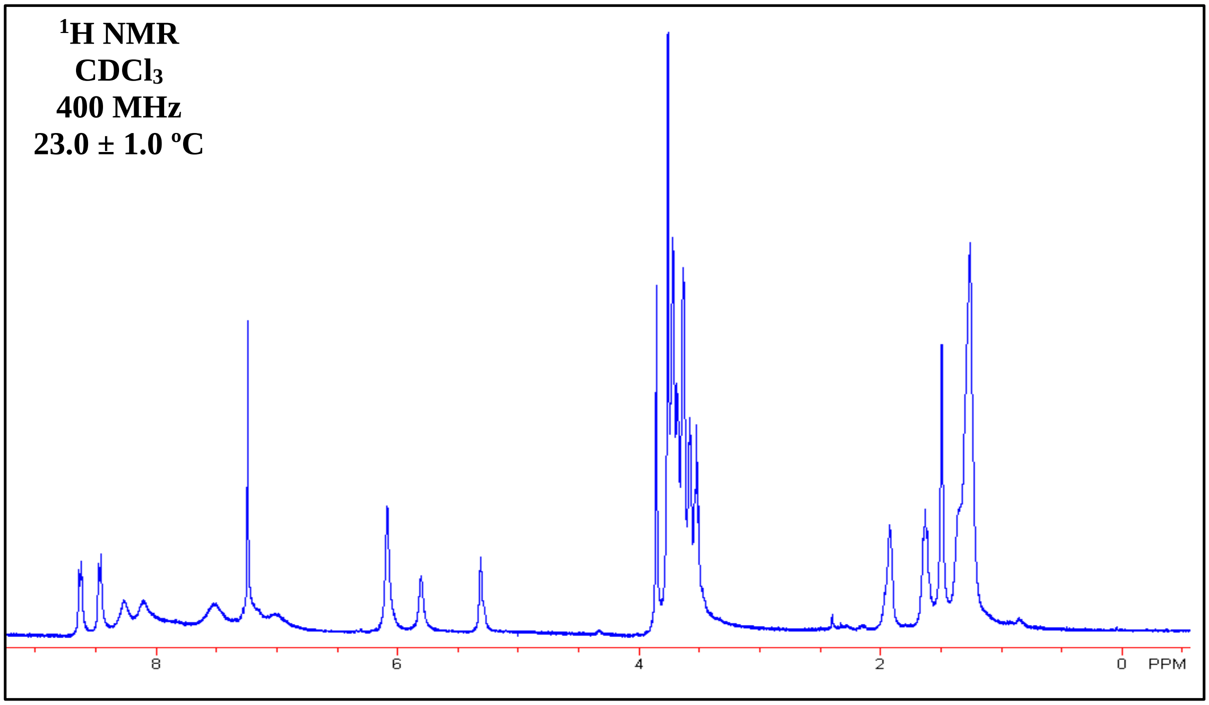

Figure S45. ^{1}H NMR spectrum (400 MHz, $CDCl_3$, 23 ºC) of oxidized metalated [2]catenane **5**.

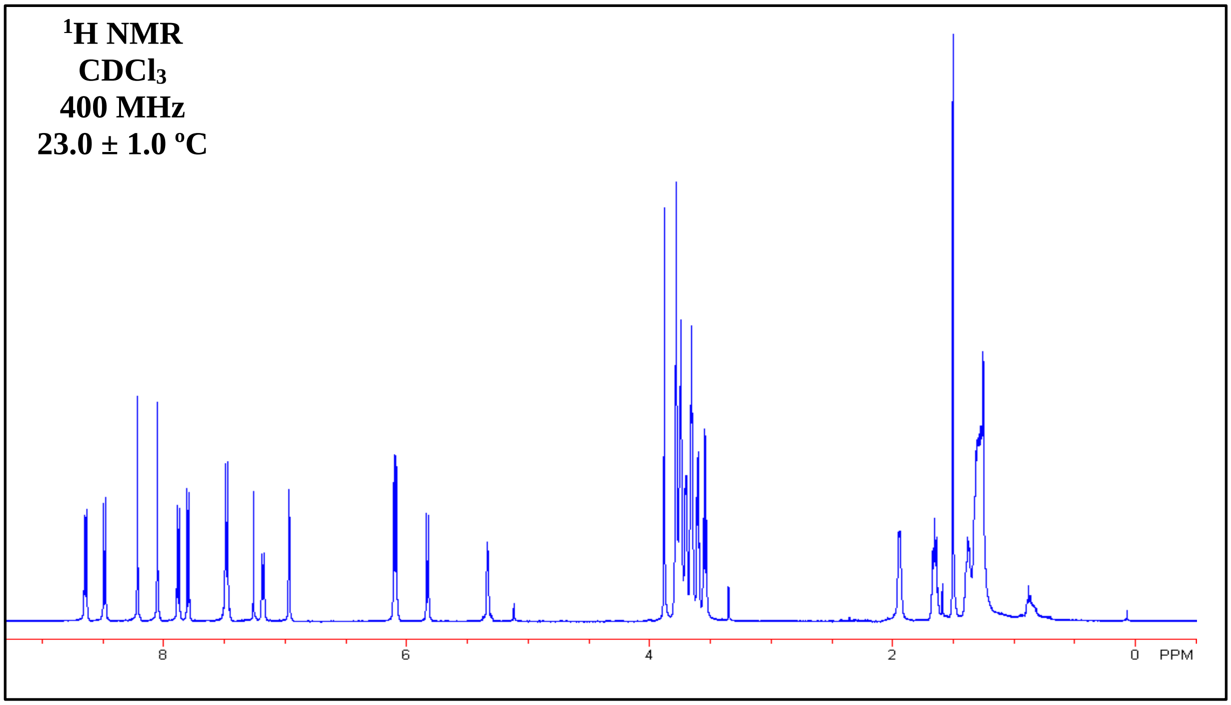

Figure S46. ^{1}H NMR spectrum (400 MHz, $CDCl_3$, 23 ºC) of reduced metalated [2]catenane **5**.

Section G. References

1. C. Dietrich-Buchecker and J.-P. Sauvage, *Tetrahedron*, 1990, **46**, 503-512.
2. M. Weck, B. Mohr, J.-P. Sauvage and R. H. Grubbs, *The Journal of Organic Chemistry*, 1999, **64**, 5463-5471.
3. S. Kang, B. M. Berkshire, Z. Xue, M. Gupta, C. Layode, P. A. May and M. F. Mayer, *Journal of the American Chemical Society*, 2008, **130**, 15246-15247.
4. S. Kang, M. M. Cetin, R. Jiang, E. S. Clevenger and M. F. Mayer, *Journal of the American Chemical Society*, 2014, **136**, 12588-12591.
5. H. W. Gibson, S. H. Lee, P. T. Engen, P. Lecavalier, J. Sze, Y. X. Shen and M. Bheda, *The Journal of Organic Chemistry*, 1993, **58**, 3748-3756.
6. P. R. Ashton, D. Philp, N. Spencer and J. F. Stoddart, *Journal of the Chemical Society, Chemical Communications*, 1992, DOI: 10.1039/C39920001124, 1124-1128.
7. Z. E. Wilson, A. M. Heapy and M. A. Brimble, *Tetrahedron*, 2007, **63**, 5379-5385.
8. Y. Kobayashi and H. Okui, *The Journal of Organic Chemistry*, 2000, **65**, 612-615.
9. W. Zhong, Y. Tang, G. Zampella, X. Wang, X. Yang, B. Hu, J. Wang, Z. Xiao, Z. Wei, H. Chen, L. De Gioia and X. Liu, *Inorganic Chemistry Communications*, 2010, **13**, 1089-1092.
10. M. Li and G. A. O'Doherty, *Organic Letters*, 2006, **8**, 3987-3990.
11. L. A. Ortiz-Frade, L. Ruiz-Ramírez, I. González, A. Marín-Becerra, M. Alcarazo, J. G. Alvarado-Rodriguez and R. Moreno-Esparza, *Inorganic Chemistry*, 2003, **42**, 1825-1834.
12. P. Schwab, R. H. Grubbs and J. W. Ziller, *Journal of the American Chemical Society*, 1996, **118**, 100-110.
13. M. Scholl, S. Ding, C. W. Lee and R. H. Grubbs, *Organic Letters*, 1999, **1**, 953-956.
14. A. Rigaku and C. Rigaku, *Journal*, 2010.
15. D. Rigaku Oxford, *Journal*, 2024.
16. G. Sheldrick, *Acta Crystallographica Section A*, 2015, **71**, 3-8.
17. G. Sheldrick, *Acta Crystallographica Section C*, 2015, **71**, 3-8.
18. O. V. Dolomanov, L. J. Bourhis, R. J. Gildea, J. A. K. Howard and H. Puschmann, *Journal of Applied Crystallography*, 2009, **42**, 339-341.
